# Percutaneous Intervention or Bypass Graft for Left Main Coronary Artery Disease? A Systematic Review and Meta-Analysis

**DOI:** 10.1155/2020/4081642

**Published:** 2020-07-26

**Authors:** Waqas Ullah, Yasar Sattar, Irfan Ullah, Ammu Susheela, Maryam Mukhtar, M. Chadi Alraies, Mamas A. Mamas, David L. Fischman

**Affiliations:** ^1^Abington Jefferson Health, Abington, PA, USA; ^2^Icahn School of Medicine, New York, NY, USA; ^3^Kabir Medical College, Peshawar, Pakistan; ^4^Loyola Medical Center, Hines, IL, USA; ^5^Rawalpindi Institute of Cardiology, Rawalpindi, Pakistan; ^6^Detroit Medical Center, Detroit, MI, USA; ^7^Keele Cardiovascular Research Group, Keele University, Keele, Royal Stoke Hospital, UK; ^8^Thomas Jefferson University, Philadelphia, PA, USA

## Abstract

**Background:**

The safety and efficacy of percutaneous coronary intervention (PCI) versus coronary artery bypass grafting (CABG) for stable left main coronary artery disease (LMCAD) remains controversial.

**Methods:**

Digital databases were searched to compare the major adverse cardiovascular and cerebrovascular events (MACCE) and its components. A random effect model was used to compute an unadjusted odds ratio (OR).

**Results:**

A total of 43 studies (37 observational and 6 RCTs) consisting of 29,187 patients (PCI 13,709 and CABG 15,478) were identified. The 30-day rate of MACCE (OR, 0.56; 95% CI, 0.42–0.76; *p* = 0.0002) and all-cause mortality (OR, 0.52; 95% CI, 0.30–0.91; *p* = 0.02) was significantly lower in the PCI group. There was no significant difference in the rate of myocardial infarction (MI) (*p* = 0.17) and revascularization (*p* = 0.12). At 5 years, CABG was favored due to a significantly lower rate of MACCE (OR, 1.67; 95% CI, 1.18–2.36; *p* = <0.04), MI (OR, 1.67; 95% CI, 1.35–2.06; *p* = <0.00001), and revascularization (OR, 2.80; 95% CI, 2.18–3.60; *p* = <0.00001), respectively. PCI was associated with a lower overall rate of a stroke, while the risk of all-cause mortality was not significantly different between the two groups at 1- (*p* = 0.75), 5- (*p* = 0.72), and 10-years (*p* = 0.20). The Kaplan–Meier curve reconstruction revealed substantial variations over time; the 5-year incidence of MACCE was 38% with CABG, significantly lower than 45% with PCI (*p* = <0.00001).

**Conclusion:**

PCI might offer early safety advantages, while CABG provides greater durability in terms of lower long-term risk of ischemic events. There appears to be an equivalent risk for all-cause mortality.

## 1. Introduction

The American College of Cardiology (ACC) and European Society of Cardiology (ESC) guidelines, updated in 2019, recommend CABG in patients with stable LMCAD with favorable coronary anatomy and low-predicted surgical mortality (class IB). The recommendations for PCI vary depending upon the anatomical complexity (low, intermediate, and high) of the unprotected LMCAD and patient complexity [1]. These guidelines were based on summated evidence from six major clinical trials. The 3-year EXCEL trial which found PCI to be noninferior to CABG was the cornerstone of these recommendations [2].

Recently reported EXCEL's 5-year results demonstrated continued noninferiority of PCI to CABG through 5 years for patients with left main CAD [3]. Of concern was the use of a new definition of MI, reportedly favoring the PCI arm, in contravention of the previous protocol, which used the Third Universal Definition (UD) of MI, developed collaboratively by the ESC and ACC. The European Association for Cardio-Thoracic Surgery (EACTS) officially withdrew their support for ESC guidelines that endorse the use of coronary stents in many patients with LMCAD.

This growing controversy and the fact that six previously conducted clinical trials have also demonstrated conflicting results regarding the management of LMCAD have prompted this meta-analysis in an attempt to provide clarity on this issue.

## 2. Methods

### 2.1. Search Strategy and Data Extraction

**Figure 1:**
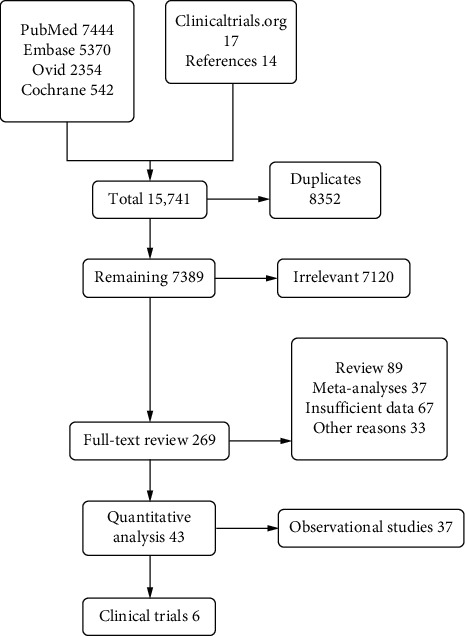
PRISMA flow diagram of the included studies.

The MEDLINE (PubMed and Ovid), Embase, Clinicaltrials.org, and Cochrane databases were queried till April 15, 2020, to identify relevant observational cohort studies (OCS) and randomized controlled trials (RCTs) (Figure 1). Studies comparing the safety and efficacy of PCI with CABG in LMCAD stenosis were included. The primary endpoint included MACCE. Secondary outcomes included components of MACCE (all-cause death, revascularization, stroke, and myocardial infarction (MI)). The detailed search strategy and map are given in Supplementary (S.). Appendix (available here).

### 2.2. Data and Quality Analysis

The statistical analysis was performed using the Cochran–Mantel–Haenszel test on a random effect model to calculate an unadjusted odds ratio (OR). Relative risk (RR) assessment for the dichotomous outcomes of RCTs was also performed. The probability value of *p* < 0.05 was considered statistically significant. The “test for overall effect” was reported as the *z* value corroborating the inference from the 95% confidence interval (CI). A stratified analysis based on the type of study (OCS vs. RCTs), angiographic SYNTAX (Synergy between Percutaneous Coronary Intervention with Taxus and Cardiac Surgery) score (<0–32 vs. >32), and different stent designs was also performed. Reconstruction of patient level data was made possible by extreme magnification and digitization of the Kaplan–Meier curves from individual RCTs. The Higgins *I*-squared (*I*^2^) statistical model was used to assess variations in outcomes of the included studies. Publication bias was illustrated graphically using a funnel plot and calculated quantitatively using Egger's Regression Equation (ERE). The methodological quality assessment of the included RCTs was performed using the Oxford scoring scale and the Cochrane collaboration tool for the systematic review and meta-analysis. The Newcastle–Ottawa Scale was used for the bias assessment of OCS. All statistical analyses were performed using the Digitize and the Cochrane Review Manager (RevMan) version 5.3.

### 2.3. Quality of the Included Studies

**Figure 2:**
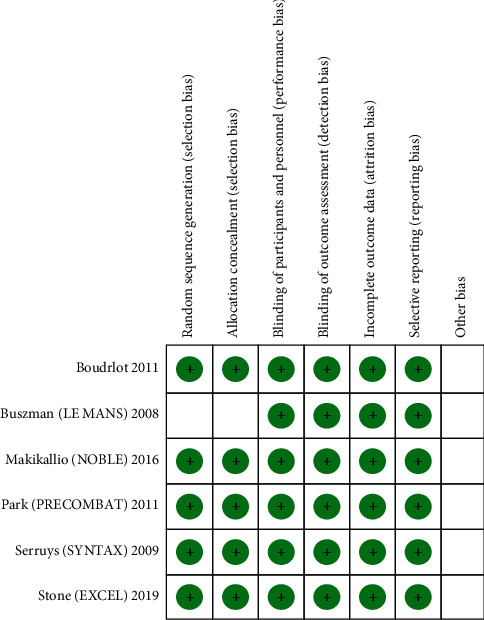
The methodological quality assessment of the included studies showing minimal risk of bias.

The overall quality of the included RCTs was high (Figure 2). Due to adequate randomization and allocation concealment in most studies, the risk of selection bias in RCTs was low. The risk of performance and detection bias was reduced due to the appropriate blinding of participants and outcomes, respectively. Similarly, reporting and attrition bias across all studies were reduced due to an adequate description of the study results and an “intention to treat model,” respectively. The Oxford scale of bias assessment showed the Jadad score ≥  3 indicating high quality of the included RCTs. The detailed quality assessments (Oxford and Newcastle–Ottawa Scale) are given in S. Tables 1, 2.

## 3. Results

### 3.1. Search Results and Study Characteristics

The initial search revealed 15,741 articles. After removal of irrelevant and duplicate items, 269 articles were deemed relevant for full-text review. Of these, 226 articles were excluded based on our selection criteria. 43 articles (6 RCTs and 37 OCS) were qualified for quantitative analysis ([3–8], S. Ref 1–48). The preferred reporting items for systematic reviews and meta-analyses (PRISMA) flow diagram is shown in Figure 1.

**Figure 3:**
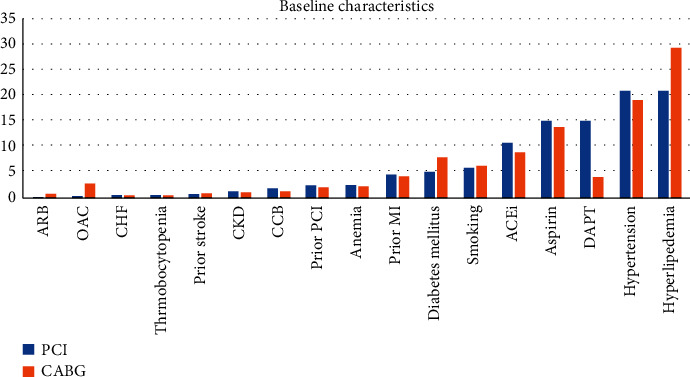
Baseline characteristics of patients undergoing PCI vs. CABG (*y*-axis percentages).

A total of 29,187 patients, 13,709 in the PCI and 15,478 in the CABG group, were included. The mean age of patients undergoing PCI was 66 and for CABG was 65 years, comprising 74% and 77% male patients, respectively. All patients had documented myocardial ischemia with ≥50% stenosis of the LMCAD. The PRECOMBAT and SYNTAX trials used the 1^st^ generation drug-eluting stents (DES), and the EXCEL and NOBLE trials used the 2^nd^ generation DES. About 10% of the NOBLE population had 1^st^ generation sirolimus-eluting Cypher stenting. Baseline characteristics of patients were comparable except for antiplatelet therapy (more in PCI) (Figure 3). The median follow-up duration was 4 years. The detailed definitions of trials, outcomes, components of MACCE, inclusion criteria of studies, and the baseline characteristics of included patients are given in S. Tables 3–7.

### 3.2. Pooled Analysis of Overall Studies

**Figure 4:**
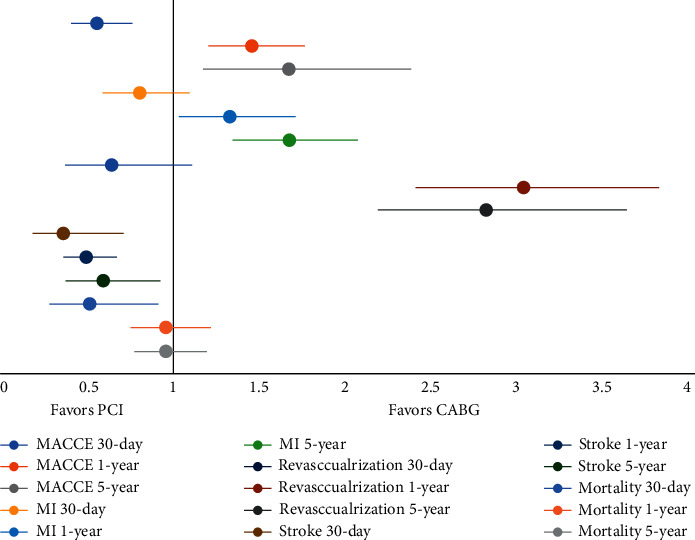
Forest plot of all the studies (RCTs + OCS) showing pooled estimates of outcomes between PCI vs. CABG across different follow-ups.

A comprehensive pooled analysis of the 43 studies favored PCI at the short-term (30 days) and CABG at long-term follow-up (1 and 5 years from randomization) (Figure 4).

The 30-day rate of MACCE (OR, 0.56; 95% CI, 0.42–0.76; *p* = 0.0002) and all-cause mortality (OR, 0.52; 95% CI, 0.30–0.91; *p* = 0.02) was significantly lower in the PCI group, while the odds of MI (OR, 0.81; 95% CI, 0.60–1.09,;*p* = 0.17), revascularization (OR, 0.65, 95% CI, 0.38–1.11; *p* = 0.12), and all-cause mortality (OR, 0.52; 95% CI, 0.30–0.91; *p* = 0.02) were comparable between the two groups. At 1 year, CABG was favored due to lower rate of MACCE (OR, 1.45; 95% CI, 1.21–1.75; *p* = <0.0001), MI (OR, 1.33; 95% CI, 1.04–1.70; *p* = 0.02), and revascularization (OR, 3.01; 95% CI, 2.40–3.79; *p* = <0.00001). CABG continued to show a favorable trend of lower rate of MACE (OR, 1.67; 95% CI, 1.18–2.36; *p* = <0.04), MI (OR, 1.67; 95% CI, 1.35–2.06; *p* = <0.00001), and revascularization (OR, 2.80; 95% CI, 2.18–3.60; *p* = <0.00001) at 5 years. The rate of stroke was significantly lower in the PCI arm at 30 days (OR, 0.37; 95% CI, 0.19–0.71; *p* = 0.03), 1 year (OR, 0.50; 95% CI, 0.37–0.67; *p* = <0.00001), and 5 year (OR, 0.60; 95% CI, 0.39–0.92; *p* = 0.02).

Only four studies reported extended data at the 10-year follow-up. There was no significant difference between the two groups in terms of MACCE (OR, 0.90; 95% CI, 0.55–1.45; *p* = 0.66), MI (OR, 1.19; 95% CI, 0.72–1.97; *p* = 0.51), revascularization (OR, 2.74; 95% CI, 0.72–10.45; *p* = 0.14), and stroke (OR, 0.73; 95% CI, 0.36–1.49; *p* = 0.38). The odds of all-cause mortality remained identical at 1 year (OR, 0.96; 95% CI, 0.76–1.22; *p* = 0.75), 5 year (OR, 0.96; 95% CI, 0.78–1.19; *p* = 0.72), and 10 year (OR, 0.85; 95% CI, 0.67–1.09; *p* = 0.20) (Table 1 and S. Figures 1–4). The heterogeneity among the outcomes of studies at 30 days to 10 years ranged from *I*^2^ = 0% to *I* = 92%.

### 3.3. Pooled Analysis of the RCTs

**Figure 5:**
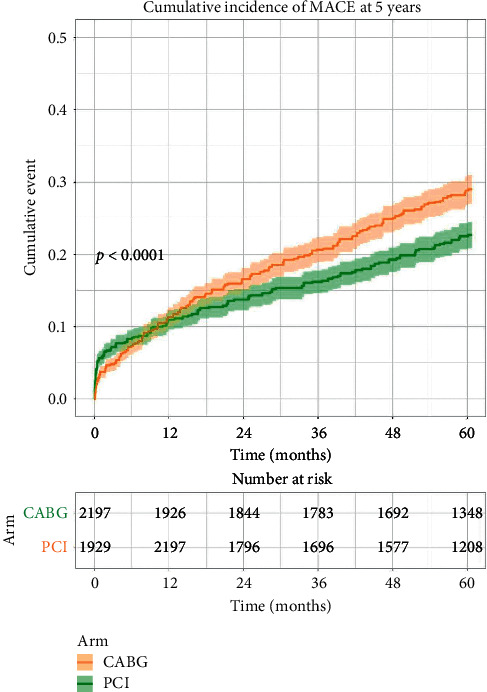
Kaplan–Meier analysis showing results favoring CABG for lower cumulative incidence of MACCE (RCTs).

**Figure 6:**
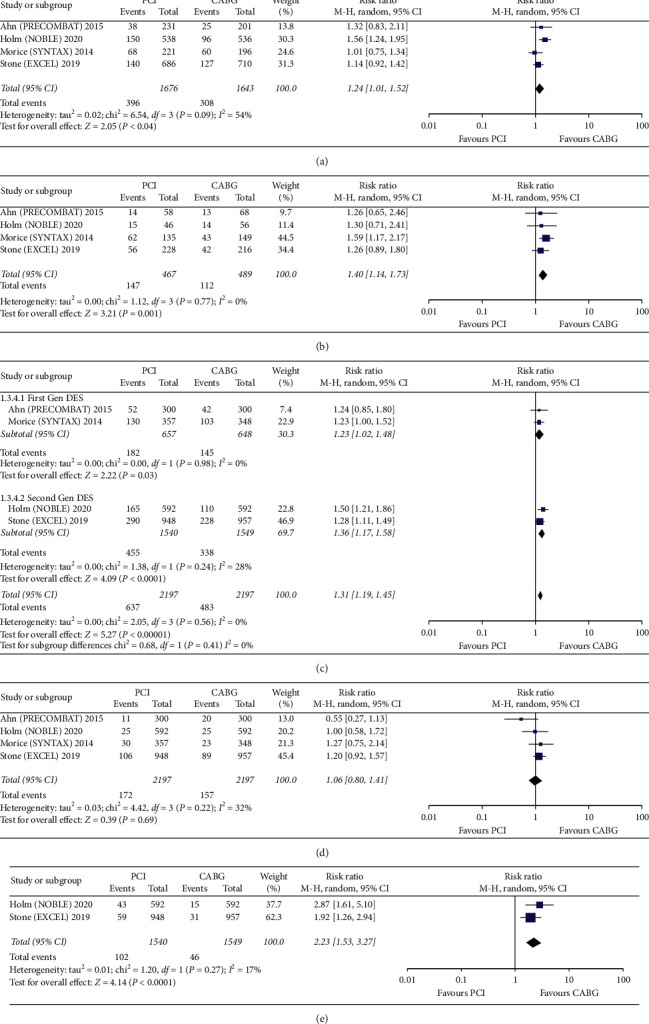
Forest plots showing an individual and pooled RR for RCTs comparing the MACCE in PCI vs. CABG for LMCAD at 5 years ((a) low SYNTAX, (b) high SYNTAX, (c) DES, (d) cardiovascular mortality, and (e) nonprocedural MI). The pooled RRs with 95% CI were calculated using random-effects models. Weight refers to the contribution of each study to the overall pooled estimate of the treatment effect. Each square and horizontal line denotes the point estimate and 95% CI for each trial's RR, respectively. The diamonds signify the pooled RR; the diamond's centre denotes the point estimate, and width denotes the 95% CI.

**Figure 7:**
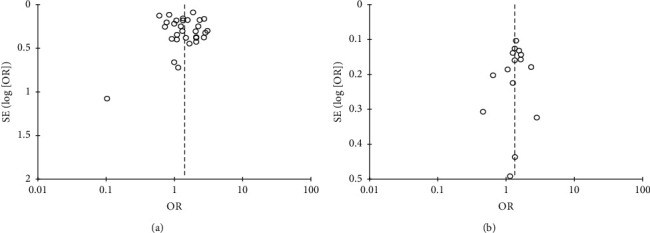
Funnel plot showing minimal publication bias across studies comparing the pooled estimate of MACCE at 1- and 5-year follow up.

Six RCTs comprising 4700 patients (PCI 2349 and CABG 2351) closely mirrored the pooled results at all time points, except that the 1-year rate of MI and MACE and the 5-year rate of stroke were identical between PCI and CABG. The heterogeneity among the outcomes of the included studies for all endpoints at 30 days and 1 year and for MACCE and revascularization after randomization was minimal (*I*^2^ = 0%–25%). Detailed results are given in S. Figures 5–7.

Kaplan–Meier analysis of four major RCTs (EXCEL, NOBLE, PRECOMBAT, and SYNTAX) showed a significant variation in the incidence of primary composite outcome over time with a cumulative rate of 45.0% (989 events) with PCI and 38.6% (849 events) with CABG at 5 years. As evident by the curve, PCI exhibited a numeric advantage over CABG in the first 30 days, while there was no significant difference in MACCE at 1 year. A significant deviation in the curves was observed in favor of CABG, from 1 to 5 years. Risk estimation by a shared frailty model at 5 years showed a significantly lower risk of MACCE in CABG (HR, 1.34; 95% CI, 1.16–1.47; *p* = <0.00001) (Figure 5).

### 3.4. Pooled Analysis of the Observational Cohort Studies

Pooled results from 37 OCS (24,487 patients: PCI 11360 and CABG 13127) were in agreement with the combined outcomes of all studies (RCTs + OCS) and with the findings of RCTs with few exceptions. In contrast to RCTs, the odds of MACCE at the 1-year follow-up were significantly lower in patients undergoing CABG (*p* = <0.0001). Unlike RCTs, CABG was superior due to significantly lower odds of MI at 1 year (*p* = 0.005) and 5 year (*p* = <0.00001). Contrary to the overall results of all studies, the odds of all-cause mortality at 30 days were identical between the two groups (OR, 0.52; 95% CI, 0.23–1.18; *p* = 0.12). The relative odds of all other endpoints in patients undergoing PCI vs. CABG were similar to the corresponding pooled risk ratios in the RCTs at all time points (S.  Table 9, S. Figures 8–13).

### 3.5. Subgroup Sensitivity Analysis of the 5-Year RCTs Data

A subgroup analysis based on the anatomical complexity of LMCAD, MI definition, cardiovascular mortality, and stent-generations favored CABG at 5 years. In contrast to pooled results, a significantly lower risk of nonprocedural (spontaneous) MI was seen in patients undergoing CABG (RR, 2.23; 95% CI, 1.53–3.27; *p* = <0.0001). These results were obtained after exclusion of periprocedural MI population (Figure 6).

The 5-year MACCE rate in a subgroup of patients with both first (RR, 1.23; 95% CI, 1.02–1.48; *p* = 0.03) and 2^nd^ generation DES (RR, 1.36; 95% CI, 1.17–1.58; *p* = <0.0001) was significantly higher compared to patients who underwent CABG (Figure 6). Interestingly, the 5-year incidence of MACCE was significantly lower in the CABG arm across all tertiles of the SYNTAX score. Both low SYNTAX score RR, 1.24; 95% CI, 1.01–1.52; *p* = 0.04, and high SYNTAX score RR, 1.40; 95% CI, 1.14–1.73; *p* = 0.001 favored CABG (Figure 6). There was no significant intergroup difference in the incidence of cardiovascular mortality (RR, 1.06; 95% CI, 0.80–1.41; *p* = 0.69) at 5 years.

### 3.6. Publication Bias

On visual assessment of the funnel plots, no significant publication bias was detected for MACCE and its individual components at 1 and 5 years (ERE ≈ *p* = 0.45 to *p* = 0.56) (Figure 7). Our funnel plots were symmetrical, indicating that the limited scatter was due to sampling variation and not publication bias (S. Figures 14–23).

## 4. Discussion

Our meta-analysis demonstrated a substantial relationship between time since randomization and effect of intervention on the primary composite endpoint. At 30 days, PCI was found to have a lower incidence of MACCE with comparable odds of MI and the need for revascularization. Patients undergoing PCI had 63% lower odds of stroke compared to CABG. These observations can be attributed to PCI being a minimally invasive procedure, dual antiplatelet therapy (DAPT), and the use of contemporary drug-eluting stents (DES). At 1 year, the rate of MACCE and MI was lower in patients undergoing CABG, albeit there was a 50% lower risk of stroke with PCI.

In contrast to the recently published EXCEL trial and all previous meta-analyses, PCI was associated with an increased risk of MACCE by 67%, at 5 years from the index procedure. A prominent impact of repeat unplanned revascularization and MI appear to drive this difference. In addition, the relative difference in stroke rate with PCI, which decreased from 63% to 40%, plausibly linked with the termination of dual antiplatelet therapy may be a contributing factor. Surprisingly, at an extended follow-up of 10 years, the risk of MACCE or its individual endpoints was identical. It can be speculated that the 10-year data were underpowered to assess the actual difference in outcomes, or the benefits of CABG were attenuated with progressive degenerative changes of the graft. Despite the prior mentioned differences in individual clinical outcomes, the risk of all-cause mortality remained identical in both groups, and this equipoise appears regardless of trial follow-up duration.

The management of unprotected LMCAD with revascularization is the accepted standard of care with clear survival benefits compared with medical management alone [9]. However, the decision about optimal revascularization strategy has been debated for years. The SYNTAX study suggested that the extent of anatomical complexity, as denoted by the SYNTAX score, should be taken into account when deciding between PCI and CABG [6]. For patients with a higher SYNTAX score (>32), the rate of MACCE was higher with PCI, while the rate was identical with a lower SYNTAX score (<32). A high rate of repeat revascularization seen with PCI was offset by a significantly higher proportion of stroke with CABG leading to a similar rate of MACCE between the two groups [6]. The PRECOMBAT trial mirrored the overall findings of the SYNTAX trial, with the exception that the SYNTAX score tertile did not impact the composite endpoint regardless of intervention strategy [8]. Contrary to these findings, the NOBLE trial found inferior outcomes in terms of primary composite endpoint with PCI compared to CABG irrespective of coronary lesion complexity [4].

The recently reported EXCEL trial added further uncertainty to this already confusing issue [3]. The arising controversy about the identical rates of the primary composite endpoint between PCI and CABG patients focus primarily on the following: (1) revascularizations were not considered as part of the reported MACCE, potentially tilting the results in favor of PCI over CABG, as evidenced by a higher MACCE in PCI (31.3% vs. 24.9%) once revascularization was taken into account. (2) Although the rate of nonprocedural MI with PCI were twice that in the CABG (6.8% vs. 3.5%), the identical odds of overall MI and unadjusted MACCE were driven by lower rate of periprocedural MIs in the PCI arm (3.9% vs. 6.1%). (3) MI diagnosis was based on CK-MB over high sensitive cardiac troponin, potentially underestimating the rate of periprocedural MI in PCI-treated patients.

On review, we found 38 prior meta-analyses, suggesting that PCI and CABG have an equivalent risk of MACCE (S.  Ref 49–86, S.  Table 10). However, in light of recent evidence, the applicability of their results is limited. Most of these studies were published before the completion of 5-year EXCEL, 5-year NOBLE, 10-year SYNTAX, and 10-year PRECOMBAT trials [3, 4, 10, 11]. All previous meta-analyses reported a crude MACCE and MI rate, unadjusted for the rate of revascularization and unstratified for periprocedural MI, respectively. The follow-up duration was short, and observational data and methodological quality were mostly missing. The most recent meta-analysis included only 5 studies, and the composite endpoint (MACCE), a commonly accepted yardstick of efficacy, was neglected [12]. Together, these limitations underestimated the long-term beneficial effects of CABG and overstated the benefits of PCI.

The present meta-analysis represents the most comprehensive study seeking to address the limitations of previous studies and assist in the clinical decision making for the management of LMCAD. Unlike previous meta-analyses, we collated findings of 43 studies, including the more contemporary clinical trials. We have demonstrated a significantly higher rate of MACCE with PCI at 5 years, irrespective of the SYNTAX score tertiles and DES generation. These findings contrast with the recent EXCEL study and all previous meta-analyses, which have tended to show an identical rate of MACCE between PCI and CABG, persisting at long follow-up durations (1–5 years).

### 4.1. Limitations

Our study is constrained by the limitations of the included studies. Patient level data were missing to determine the impact of PCI techniques or account for the differential use of antiplatelet agents. CABG-related complications such as bleeding, renal failure, and postprocedure infections were not assessed. The cumulative incidence of outcomes at 10 years was underpowered. Moreover, due to the differential risk of need for revascularization after the index procedure (PCI vs. CABG) and unmeasured confounding factors, individual patient level data such as patient age, life expectancy, operators skills, aortic calcifications, patient comorbidities, and bleeding risks should be considered as a cornerstone during the decision making process for LMCAD intervention.

## 5. Conclusion

In patients with LMCAD, PCI might offer early safety advantages, while CABG seems to provide greater durability in terms of consistently lower risk of MACCE and reduced need for revascularization. There appears to be an equivalent risk for all-cause mortality between both procedures.

## Figures and Tables

**Table 1:** Pooled results of all 43 studies showing comparison of odds ratio with 95% CI for primary and secondary endpoints of PCI vs. CABG for across all follow-up duration.

Event	30 days	1 year	5 years	10 years
MACCE	0.56 (0.42–0.76, *p* = 0.0002)	1.45 (1.21–1.75, *p* = <0.0001)	1.67 (1.18–2.36, *p* = 0.04)	0.68 (0.44–1.06, *p* = 0.09)
MI	0.81 (0.60–1.09, *p* = 0.17)	1.33 (1.04–1.70, *p* = 0.02)	1.67 (1.35–2.06, *p* = <0.00001)	1.21 (0.67–2.18, *p* = 0.53)
Revascularization	0.65 (0.38–1.11, *p* = 0.12)	3.01 (2.40–3.79, *p* = <0.00001)	2.80 (2.18–3.60, *p* = <0.00001)	2.95 (0.22–39.28, *p* = 0.41= 0.41)
Stroke	0.37 (0.19–0.71, *p* = 0.03)	0.50 (0.37–0.67, *p* = <0.00001)	0.60 (0.39–0.92, *p* = 0.02)	0.68 (0.28–1.65, *p* = 0.39)
All-cause mortality	0.52 (0.30–0.91, *p* = 0.02)	0.96 (0.76–1.22, *p* = 0.75)	0.96 (0.78–1.19, *p* = 0.72)	0.79 (0.60–1.05, *p* = 0.10)

## Data Availability

Data were obtained from published articles on the topic. All data can be obtained from the references mentioned in the supplementary file. The consolidated data are available from the corresponding author upon request.

## Supplementary Materials

Supplementary Materials The Supplementary Appendix contains a search map, additional tables, figures, and quality assessment tools.
