# Supplementary material for: Percutaneous Intervention or Bypass Graft for Left Main Coronary Artery Disease? A Systematic Review and Meta-Analysis
Source: J Interv Cardiol. 2020 Jul 26;2020:4081642. doi: 10.1155/2020/4081642 (PMC7399756; doi:10.1155/2020/4081642)
Supplement: Supplementary Materials — The Supplementary Appendix contains a search map, additional tables, figures, and quality assessment tools. [file 4081642.f1.pdf]

Supplementary Appendix

TITLE:

Percutaneous Intervention or Coronary Artery Bypass Graft for Left Main Stem Coronary Artery Disease? A Systematic Review and Meta-Analysis

Abbreviations:

PRECOMBAT: Premier of Randomized Comparison of Bypass Surgery versus Angioplasty Using Sirolimus-Eluting Stent in Patients with Left Main Coronary Artery Disease
SYNTAX: Synergy between Percutaneous Coronary Intervention with Taxus and Cardiac Surgery
EXCEL: Evaluation of XIENCE Versus Coronary Artery Bypass Surgery for Effectiveness of Left Main Revascularization
NOBLE:Nordic-Baltic-British Left Main Revascularization Study
LEMANS:Left Main Stenting Versus Bypass Surgery

CONTENTS:

S.No	Contents	Description
1.	Title	Text
2.	Additional Results	Text
3.	Search Strategy	Text
4.	Quality Assessment of the included RCTs	S. Table 1
5.	Quality Assessment of the included observational studies	S. Table 2
6.	Definitions of Outcomes	S. Table 3
7.	Inclusion Criteria of the included Trials and MACCE Components	S. Table 4
8.	Definitions and Descriptions of Terminologies/Scores	S. Table 5
9.	Baseline characteristics of the included RCTs	S. Table 6
10.	Baseline characteristics of the included observational studies	S. Table 7
11.	Pooled results of the included RCTs	S. Table 8
12.	Pooled results of the included observational studies	S. Table 9
13.	Results and limitations of previous meta-analyses	S. Table 10
14.	Supplementary forest plots of detailed results of the studies	S. Figures 1-13
15.	Supplementary funnel plots for all studies	S. Figures 14-23
16.	Supplementary References of the RCTs, OCS and Meta-analyses	List

A. ADDITIONAL RESULTS:

On a stratified analysis of RCTs only, PCI was favored due to a lower risk of MACCE (RR 0.61, 95% CI, 0.47-0.79, p=0.0002) and stroke (RR 0.41, 95% CI 0.17-0.98, p=0.05) and no significant difference in the risk of MI (RR 0.76, 95% CI 0.54-1.05, p=0.10), revascularization (RR 0.64, 95% CI 0.34-1.20, p=0.17) and all-cause mortality (RR 0.58, 95% CI 0.30-1.16, p=0.12) at 30-days. At 1 year, CABG showed significant benefits in terms of the lower need for revascularization (RR 1.71, 95% CI 1.39-2.10, p=<0.00001), while PCI was favored due to a lower rate of stroke (RR 0.32, 95% CI 0.15-0.69, p=0.004). While CABG did show a numerical advantage of a lower rate of MACCE and MI over PCI, this difference did not reach the statistical significance; (RR 1.07, 95% CI 0.93-1.23, p=0.33 and RR 1.06, 95% CI 0.72-1.57, p=0.76), respectively. Similarly, there was no significant difference in the risk of all-cause mortality (RR 0.83, 95% CI 0.63-1.07, p=0.15). A trend favoring CABG for MACCE (RR 1.31, 95% CI 1.19-1.45, p=<0.00001) and revascularization (RR 1.72, 95% CI 0.47-2.00, p=<0.00001) was observed at follow-up duration of 5-years. Nonetheless, the risk of all-cause mortality (RR 1.07, 95% CI 0.84-1.36, p=0.57) and MI (RR 1.62, 95% CI 1.0-2.62, p=0.05) was consistently identical between the two groups. In contrast to 1-year results, no significant difference was observed in the risk of stroke (RR 0.86, 95% CI 0.44-1.68, p=0.66). (S. Figure 5-7, S. Table 8)

B. SEARCH STRATEGY and MAP:

The MEDLINE (PubMed, Ovid), Embase, Clinicaltrials.org and Cochrane databases were queried with various combinations of medical subject headings (MeSH) to identify relevant articles. There were no language or time restrictions placed. Backward snowballing was performed to retrieve unidentified studies that were missed on the initial search. The MeSH used included two subsets: one for PCI using the terms like "PCI," "angiography," "percutaneous intervention," "coronary stenting," "drug-eluting stents," "cardiac catheterization', "left heart cath," and the other for CABG using "left main coronary artery bypass graft," "coronary graft," "LMCAD graft," and "CABG." The two subsets of MeSH were combined in a 1:1 combination using Boolean operators. Results from all possible combinations were downloaded into an EndNote library. All randomized control trials (RCT) and observational cohort studies (OCS) until February 20, 2020, were evaluated. Studies comparing the safety and efficacy of PCI with CABG in LMCAD stenosis were included. The primary endpoint was a composite of major adverse cardiovascular and cerebrovascular events (MACCE). Secondary outcomes included individual components of MACCE [all-cause death, revascularization, stroke, and myocardial infarction (MI)]. Review articles, case reports, conference papers, and studies with no control arm or insufficient data were excluded. Patients with acute coronary syndrome were excluded.

((((((((((((PCI AND left main left main coronary artery bypass graft)) OR (angiography AND left main left main coronary artery bypass graft)) OR (percutaneous intervention AND left main left main coronary artery bypass graft)) OR (coronary stenting AND left main left main coronary artery bypass graft)) OR (drug-eluting stents AND left main left main coronary artery bypass graft)) OR (cardiac catheterization AND left main left main coronary artery bypass graft)) OR (left heart cath AND left main left main coronary artery bypass graft)))) OR ((((((((((PCI AND CABG)) OR (angiography AND CABG)) OR (percutaneous intervention AND CABG)) OR (coronary stenting AND CABG)) OR (drug-eluting stents AND CABG)) OR (cardiac catheterization AND CABG)) OR (left heart cath AND CABG)))) OR ((((((((((PCI AND LMCAD graft)) OR (angiography AND LMCAD graft)) OR (percutaneous intervention AND LMCAD graft)) OR (coronary stenting AND LMCAD graft)) OR (drug-eluting stents AND LMCAD graft)) OR (cardiac catheterization AND LMCAD graft)) OR (left heart cath AND LMCAD graft)))

C. SUPPLEMENTAL TABLES:

S. Table 1

Randomized studies quality assessment using the Oxford Quality Scoring System.
(Jadad score ≥ 3 considered high quality)

Author/Study/Year/Ref	Rating Scale List	Response	Jadad Score
**Serruys (SYNTAX) 2009 [9]**	Was the study described as random	**Yes**	**3**
	Was the randomization described and appropriate	**Yes**	
	Was the study described as double-blind	**No**	
	Was the method of double-blinding appropriate	**No**	
	Was there a description of dropouts and withdrawals	**Yes**	
**Makikallio (NOBLE) 2016 [10]**	Was the study described as random	**Yes**	**3**
	Was the randomization described and appropriate	**Yes**	
	Was the study described as double-blind	**No**	
	Was the method of double-blinding appropriate	**No**	
	Was there a description of dropouts and withdrawals	**Yes**	
**Park (PRECOMBAT) 2011 [6]**	Was the study described as random	**Yes**	
	Was the randomization described and appropriate	**Yes**	**3**
	Was the study described as double-blind	**No**	
	Was the method of double blinding appropriate	**No**	
	Was there a description of dropouts and withdrawals	**Yes**	
**Stone (EXCEL) 2019 [1]**	Was the study described as random	**Yes**	**3**
	Was the randomization described and appropriate	**Yes**	
	Was the study described as double-blind	**No**	
	Was the method of double-blinding appropriate	**No**	
	Was there a description of dropouts and withdrawals	**Yes**	
**Buszman (LE MANS) 2008 [22]**	Was the study described as random	**Yes**	**2**
	Was the randomization described and appropriate	**No**	
	Was the study described as double-blind	**No**	
	Was the method of double blinding appropriate	**No**	
	Was there a description of dropouts and withdrawals	**Yes**	
**Boudriot et al. 2011 [9]**	Was the study described as random	**Yes**	**3**
	Was the randomization described and appropriate	**Yes**	
	Was the study described as double-blind	**No**	
	Was the method of double blinding appropriate	**No**	
	Was there a description of dropouts and withdrawals	**Yes**	

S. Table 2

NewCastle-Ottawa scale (NOS) for assessing quality of observational studies

**Selection Bias**					**Outcome Bias**				
**Author/Study/Year**	**Representativeness of the exposed cohort**	**Selection of the nonexposed cohort**	**Ascertainment of exposure**	**Outcome not present at baseline**	**Comparability of the cohort**	**Assessment of outcome**	**Enough follow-up duration**	**Adequate follow-up**	**Total score**
Brener 2008	*	*	*	*	**	*	*	-	8
**Buchanan (Delta Registry) 2014**	*	*	*	*	**	*	*	-	8
**Caggegi (CUSTOMIZE) 2011**	*	*	*	*	**	*	*	-	8
**Cavalcante 2016**	*	*	-	*	*	*	*	-	6
**Chang 2012**	*	*	*	*	*	*	*	-	7
**Chieffo 2012**	*	*	*	*	*	*	*	-	7
**Cheng 2009**	*	*	*	*	*	*	*	-	7
**Ghenim 2009**	*	*	*	*	*	*	*	-	7
**Hong 2005**	*	*	*	*	**	*	*	-	8
**Kang 2010**	*	*	*	*	*	*	*	-	7
**Kawecki 2012**	*	*	*	*	*	*	*	*	8
**Kim 2009**	*	*	*	*	*	-	*	*	7
**Lee 2006**	*	*	*	*	**	*	*	-	8
**Luo 2012**	*	*	*	*	*	*	*	-	7
**Lu 2016**	*	*	-	*	*	*	-	-	5
Makikikallio 2008	*	*	*	*	**	*	*	*	9
Makikikallio 2016	*	*	*	*	**	*	*	*	9
**Montalescot 2009**	*	*	*	*	*	*	*	*	8
**Naganuma 2014**	*	*	*	*	*	*	*	-	7
**Palmerini 2006**	*	*	*	*	**	*	*	*	9
**Park DW 2010**	*	*	*	*	**	*	*	*	9
**Park (MAIN COMPARE) 2011**	*	*	-	*	*	-	*	-	4
**Park SJ 2011**	*	*	*	*	*	-	-	-	5
**Qin 2013**	*	*	*	*	*	*	*		7
**Rittger 2011**	*	*	*	*	*	*	*	*	8
**Rodes- Cabau 2008**	*	*	*	*	**	*	*	-	8
**Sanmartin 2007**	*	*	*	*	**	*	*	-	8
**Shimizu 2010**	*	*	*	*	*	*	*	*	8
**Shiomi (CREDO KYOTO 2 2015**	*	*	-	*	*	*	*	-	6
**Stone EXCEL 2019**	*	*	*	*	**	*	*	*	9
**Stone EXCEL 2016**	*	*	-	*	*	*	-	-	5
**Thiele 2009**	*	*	*	*	*	*	*	-	7
**Te Hsu 2008**	*	*	*	*	**	*	*	-	8
**Wei 2016**	*	*	*	*	*	*	-	-	6
White 2008	*	*	*	*	**	*	*	*	9
**Wu 2008**	*	*	*	*	**	*	*	*	9
**Wu 2010**	*	*	*	*	**	*	*	-	8
**Yi Gijong 2012**	*	*	*	*	*	*	*		7
**Yin 2015**	*	*	-	*	-	-	-	-	3
**Yu 2016**	*	*	-	*	-	-	*	-	4
**Zhao 2011**	*	*	-	*	*	*	*	-	6
**Zheng 2016**	*	*	-	*	-	-	*	*	5

The methodological quality of retrospective or prospective observational studies was done using Newcastle–Ottawa scale (NOS) quality scale. Each asterisk/star in the Newcastle-Ottawa Scaling System (NOS) represents responses of the biases questionnaire. Each bias assessment part gets one star except comparability that gets a maximum of 2 stars. Each star counts towards the total score. Score <5 represents poor quality, 5-6 represents moderate quality and 7 to 9 are considered as high quality. Total of 30 studies had a NOS score >7 representing a high quality. Rest of the studies had moderate to poor quality owing to the ascertainment bias, comparability, and follow up limitations.

- Not Available or unable to extrac

S. Table 3

Definition of outcomes used across the randomized controlled trials

Outcome	Description
**Death**	The cause of death was adjudicated as being due to cardiovascular or non-cardiovascular causes<br>• Cardiovascular death includes sudden cardiac death, death due to acute MI, heart failure or cardiogenic shock, stroke, other cardiovascular causes, or bleeding<br>• Non-cardiovascular death is defined as any death with known cause not of cardiac or vascular causes.
**MI**	**Periprocedural MI:**<br>Defined as the occurrence within 48-72 hours after either PCI or CABG<br><br>**SCAI Definition:**<br>• CK-MB >10x upper reference limit (URL)*, OR<br>• CK-MB >5x URL*, PLUS<br>- new pathological Q waves in at least 2 contiguous leads or new persistent non-rate related LBBB, or angiographically documented graft or native coronary artery occlusion or new severe stenosis with thrombosis and/or diminished epicardial flow, or imaging evidence of new loss of viable myocardium or new regional wall motion abnormality<br><br>**Non-procedural MI:** SCAI defined non-procedural MI as the occurrence >72 hours after any PCI or CABG.<br><br>**SCAI Definition:**<br>• The rise and/or fall of cardiac biomarkers (CK-MB or troponin) >1x URL*<br>PLUS:<br>- ECG changes indicative of new ischemia [ST-segment elevation or depression, or bundle branch block (BBB)], or<br>- Development of pathological Q waves (≥0.04 seconds in duration and ≥1 mm in depth) in ≥2 contiguous precordial leads or ≥2 adjacent limb leads) of the ECG, or<br>- Angiographically documented graft or native coronary artery occlusion or new severe stenosis with thrombosis and/or diminished epicardial flow, or Imaging evidence of new loss of viable myocardium or new regional wall motion abnormality<br><br>**Third Universal Definition of MI:**<br>Detection of a rise and/or fall of cardiac biomarker values, with at least one of the values being elevated (i.e., > 99th percentile upper reference limit, URL). The preferred cardiac biomarker of necrosis is highly sensitive and specific cTn.
**Stroke**	The rapid onset of a new persistent neurologic deficit attributed to an obstruction in cerebral blood flow
**Revascularization**	A coronary revascularization procedure may be either a CABG or a PCI.
**MACCE**	Major adverse cardiovascular or cerebrovascular events were defined as all-cause mortality, stroke or transient ischemic attack, nonfatal MI, acute coronary syndrome (including unstable angina), and left ventricular failure requiring hospital admission.

S. Table 4

Inclusion Criteria of RCTs.

Trials	Inclusion Criteria	MACCE Components
LE MANS	Stable LMCAD >50% stenosis, symptomatic, documented myocardial ischemia	MI, death, stroke, and revascularization
EXCEL	Stable LMCAD 50-70% stenosis, symptomatic	MI, death, and stroke
NOBLE	Stable, unstable angina, ACS, LMCAD >50% stenosis, FFR <0.80, symptomatic, documented myocardial ischemia	MI, death, stroke, and revascularization
PRECOMBAT	Stable, unstable angina, NSTEMI, LMCAD >50% stenosis, symptomatic, documented myocardial ischemia	MI, death, stroke, and revascularization
SYNTAX	Stable LMCAD or multivessel >50% stenosis, symptomatic, documented myocardial ischemia	MI, death, stroke, and revascularization
Boudriot et al.	Stable LMCAD >50% stenosis, symptomatic, documented myocardial ischemia	MI,death, and revascularization

S. Table 5

Definitions and Descriptions of Terminologies/Scores

Terminology	Definition
SYNTAX Score	The SYNTAX score is a grading system that evaluates the complexity and prognosis of patients undergoing percutaneous coronary intervention (PCI). The SYNTAX score is the sum of the points assigned to each individual lesion identified in the coronary tree with greater than 50% diameter narrowing in vessels of greater than 1.5 mm diameter. The coronary tree is divided into 16 segments according to the AHA classification.
Ascertainment bias	Ascertainment bias is a systematic distortion in measuring the true frequency of a phenomenon due to the way in which the data are collected

S. Table 6

Selected baseline characteristics of randomized trials.

Author/Study/ Year	PCI-CA BG	Mean Age	Male %	HTN	DM	HLD	Smokers	Mean Syntax Score	EuroScor e	Site of Lesion	FU
**Ahn-PRECOMBAT 2015 [1]**	300-300	62-63	76-77	-	102 (34)-90 (30)	-	-	-	-	D	5
**Boudriot et al. 2011 [2]**	100-101	66-69	72-77	82 (82)-83 (82)	40 (40)-33 (33)	68 (68)-65 (64)	35 (35)-28 (28)	24-23	2.4-2.6	Ost/D	1
**Buszman-LE MANS 2016 [3]**	52-53	60-61	60-73	39 (75)-37 (70)	10 (19)-9 (17)	34 (65)-32 (60)	-	25.2-24.7	3.3-3.5	-	10
**Holm-NOBLE 2020 [4]**	592-592	66-66	80-76	386 (66)-389 (66)	94 (16)-90 (15)	482 (81)-464 (78)	108 (18)-127 (21)	22-22	2-2	Ost/MS/B	5
**Mäkikallio-NOBLE 2016 [5]**	592-592	66-66	80-76	386 (66)-389 (66)	86 (15)-90 (15)	482 (81)-464 (78)	108 (18)-127 (21)	22-22	2-2	Ost/MS/B	3
**Morice-SYNTAX 2014 [6]**	357-348	NA-66	74-76	250 (70)-215 (62)	85 (24)-89 (26)	289 (81)-261 (75)	64 (18)-83 (24)	29-30	3.9-3.9	Ost/MS/B	5
**Park-PRECOMBAT 2015 [7]**	300-300	62-63	76-74	-	102 (34)-90 (30)	-	-	-	-	D	1
**Stone-EXCEL 2019 [8]**	948-957	66-66	21-19	703 (74)-701 (73)	72 (8)-62 (7)	668 (70)-652 (68)	222 (23)-193 (20)	20-20	-	-	5
**Stone-EXCEL 2016 [9]**	948-957	66-66	76-77	703 (74)-701 (73)	286 (30)-268 (28)	668 (70)-652 (68)	222 (23)-193 (20)	20-20	-	B	3
**Serruys-SYNTAX 2009 [10]**	357-348	65-65	74-76	250 (70)-215 (62)	87 (25)-89 (26)	289 (81)-261 (75)	64 (18)-83 (24)	29-30	-	Ost/MS/B	1
**Thuijs-SYNTAX 2019 [11]**	357-348	65-65	76-79	246 (69) - 257(74)	87 (25)-89 (26)	289 (81)-261 (75)	64 (18)-83 (24)	29-30	-	Ost/MS/B	10

All data is presented in the format of PCI/ CABG if applicable.

PCI: Percutaneous Coronary Intervention, CABG: Coronary Artery Bypass Graft HTN= Hypertension, DM= Diabetes Mellitus, HL= Hyperlipidemia, SYNTAX: Synergy between percutaneous coronary intervention (PCI) with taxus and cardiac surgery, EuroScore: European System for Cardiac Operative Risk Evaluation, Ost: Ostial, MS: Midshaft, D: Distal Bifurcation, B: Bifurcation, PRECOMBAT: Bypass Surgery Versus Angioplasty Using Sirolimus-Eluting Stent in Patients With Left Main Coronary Artery Disease, Le MANS: Left Main Coronary Artery Stenting, EXCEL: Evaluation of XIENCE Versus Coronary Artery Bypass Surgery for Effectiveness of Left Main Revascularization.

-Not available or unable to extract, * Age presented as - mean PCI - mean CABG (%) = Percentages

S. Table 7

Selected baseline characteristics of observational studies.

Author/Study/ Year	PCI-CABG	Mean Age	Male %	HTN	DM	HLD	Smokers	Syntax Score	EuroScore	Site of Lesion	FU
**Brener 2008 [12]**	97-190	68-68	72-74	82 (85)-144 (76)	42 (43)-25 (13)	-	11 (11)-101 (53)	-	4.6-4.5	-	3
**Buchanan Delta 2014 [13]**	489-328	67-68	-	359 (73)-240 (73)	161 (33)-101 (31)	323 (66)-232 (71)	122 (25)-55 (17)	26.8-37.1	5.6-5.4	-	3
**Caggegi CUSTOMIZE 2011 [14]**	222-361	67-66	76-78	159 (72)-266 (74)	79 (36)-146	123 (55)-191	97 (44)-162 (49)	26-33.6	6.3-5.6	Ost-MS-DB	1
**Cavalcante 2016 [15]**	657-648	64-64	74-76	-	187 (28) -179 (28)	416 (63)-380 (59)	153 (23)-165 (25)	27-28	3.3-3.4	-	5
**Chang 2012 [16]**	558-309	64-65	73-72	330 (59)-171 (55)	184 (33)-121 (39)	177 (32)-102 (33)	135 (24)-80 (26)	25-34	3.8-4.2	-	5
**Cheng 2009 [17]**	94-216	68-67	74-76	68 (72)-155 (72)	32 (34)-108 (50)	67 (71)-97 (45)	18 (19)-64 (30)	-	6.9-6.4	Ost/MS/B	1
**Chieffo 2012 [18]**	1874-900	66-66	74-64	1200 (64)-609 (68)	520 (28) -306 (34)	1159 (62)-582 (65)	847 (45)-384 (43)	28-38	4.9-5.1	Ost/MS/B	4
**Ghenim 2009 [19]**	105-106	81-79	64-72	69 (66)-77 (73)	25 (24)-32 (30)	44 (42)-67 (63)	26 (25)-21 (20)	-	7-8	D	1
**Kang 2010 [20]**	205-257	64-66	70-74	130 (63)-173 (67)	77 (38)-112 (44)	112 (55)-153 (60)	89 (43)-127 (49)	-	4.2-5.6	Ost/shaft/B	3
**Kawecki 2012 [21]**	34-111	67-66	68-73	25 (74)-80 (72)	6 (18)-34 (30)	16 (47)-45 (41)	4 (12)-19 (17)	-	4.7-4.8	Ost/MS/B	1
**Kim 2009 [22]**	251-256	64-66	71-72	418-343	251 (100)-256 (100)	241 (96)-254 (99)	166 (66)-22 (9)	-	4-5	Ost/MS/DB	3
**Lee 2007 [23]**	50-123	72-70	50-76	44 (88)-99 (81)	18 (36)-38 (31)	37 (74)-88 (72)	6 (12)-23 (19)	-	-	Ost/MS	1
**Lu 2016 [24]**	208-270	70-69	84-86	163 (78)-223 (83)	98 (47)-124 (46)	112 (54)-135 (50)	104 (50)-180 (67)	-	7.1-6.4	D/B	5
**Luo 2012 [25]**	331-492	61-63	79-80	194 (59)-294 (60)	99 (30)-127 (26)	55 (17)-102 (21)	150 (45) -199 (40)	-	5-5	Ost/MS/DB	2
**Makikikallio 2008 [26]**	49-238	72-70	59-80	23 (47)-108 (45)	10 (20)-40(17)	-	10 (20)-43 (18)	-	7.7-5.2	Ost/D	1
**Montalescot 2009 [27]**	514-612	-	73-74	321 (62)-430 70)	140 (27)-169 (28)	233 (45)-351 (57)	271 (53)-370 (60)	-	-	-	6
**Naganuma 2014 [28]**	482-374	64-67	73-62	307 (64)-264 (71)	127 (26)-138 (37)	284 (59)-240 (64)	231 (48)-169 (45)	26-35	4.5-5.2	Ost/MS	3.5
**Palmerini 2006 [29]**	157-154	73-69	70-76	109 (69)-112 (72)	41 (26)-39 (25)	98 (62)-111 (72)	76 (48)-74 (48)	-	6-5	Ost/MS/B	≥1
**Park DW 2010 [30]**	176-219	61-62	71-74	83 (47)-121 (55)	52 (30)-81 (37)	62 (35)-121 (55)	31 (18)-43 (20)	-	3.3-4.5	Ost/MS/DB	5
**Park DW 2010 [30]**	100-250	55-61	60-74	23 (23)-125 (50)	21 (21)-82 (33)	34 (34)-115 (46)	36 (36)-68 (27)	-	3.3-4.4	Ost/MS/DB	10
**Park MAIN COMPARE 2011 [31]**	784-690	62-64	70-72	418 (53)-343 (50)	251 (32)-256 (37)	240 (31)-253 (37)	193 (25)-178 (26)	-	-	Ost/MS/DB	5
**Park SJ 2011 [32]**	300-300	62-68	76-77	163 (54)-154 (51)	102 (34)-90 (30)	127 (42)-120 (40)	89-83	-	2.6-2.8	B involved	2
**Qin 2013 [33]**	233-282	65-67	84-87	132 (57)-195 (69)	57 (24)-77 (27)	82 (35)-113 (40)	112 (48)-113 (40)	24-34	3.7-4.5	B: 155/213	≥2
**Rittger 2011 [34]**	95-192	73-73	76-72	84 (88)-124 (65)	35 (37)-68 (35)	54 (57)-194 (100)	-	-	-	Ost/MS/DB	1
**Rodes-Cabau 2008 [35]**	104-145	85-82	54-63	78 (75)-105 (72)	28 (27)-38 (26)	63 (61)-119 (82)	3 (3)-9 (6)	-	9.5-8.4	Ost/MS/DB	2
**Sanmartin 2007 [36]**	96-245	66-66	81-87	42 (44)-148 (60)	18 (19)-78 (32)	40 (42)-112 (46)	37 (39)-112 (46)	-	25% >6	Ost/MS/D	≥1
**Shimizu 2010 [37]**	64-89	71-70	81-85	54 (84)-70 (79)	31 (48)-41 (46)	29 (45)-52 (58)	42 (66)-58 (65)	-	-	-	2
**Shiomi CREDO-KYOTO-2 2015**	364-640	71-69	71-77	312 (86)-542 (85)	154 (42)-291 (45)	117 (32)-112 (18)	78 (21)-157 (25)	26-30	-	-	5
**[38]**	20-39	66-66	60-69	8 (40)-20 (51)	9 (45)-14 (36)	5 (25)-12 (31)	4 (20)-11 (28)	-	6.3-5.5	Ost/MS/DB	1
**Te Hsu 2008 [39]**	64-62	74-71	75-79	39 (61)-45 (73)	28 (44)-21 (34)	11 (17)-13 (21)	25 (39)-36 (58)	27-35	6.8-6	Ost/MS/B	1
**Wei 2016 [40]**	120-223	71-69	58-77	90 (76)-170 (76)	42 (36)-60 (27)	89 (75)-171 (77)	21 (18)-37 (17)	-	-	Ost/MS/DB	3
**White 2008 [41]**	135-135	71-68	64-71	-	29 (21)-29 (21)	-	-	-	-	-	1
**Wu 2008 [42]**	131-245	61-NA	75-82	85 (65)-153 (62)	35 (27)-71 (29)	42 (32)-75 (31)	51 (39)-96 (39)	-	4.3-4.2	Ost/MS/D	3
**Wu 2010 [43]**	128-128	62-65	72 -77	76 (56)-80 (63)	42 (33)-40 (31)	-	-	-	-	MS/D/B	5
**Yi Gijong 2012 [44]**	106-121	62-61	68-56	71 (67)-79 (65)	23 (22)-26 (21)	47 (44)-66 (55)	34 (32)-37 (31)	26-32	-	Ost/MS/B	1
**Yin 2015 [45]**	465-457	62-64	79-82	286 (62)-269 (59)	143 (31)-131 (29)	231 (50)-158 (35)	230 (49)-205 (45)	-	5-5	Ost/MS/B	7
**Yu 2016 [46]**	56-116	-	73-72	32 (57)-60 (52)	56 (100)-116 (100)	44.6-42-4	28 (50)-51 (44)	-	-	DB/total occlusion	2
**Zhao 2011 [47]**	1442-2604	60-62	82-79	782 (54)-1674 (64)	348 (24)-806 (31)	722 (50)-1539 (59)	671 (47)-1395 (54)	23-33	1.8-2.8	Ost/MS/B	3
**Zheng 2016 [48]**											

All data is presented in the format of PCI/ CABG if applicable.
PCI: Percutaneous Coronary Intervention, CABG: Coronary Artery Bypass Graft HTN= Hypertension, DM= Diabetes Mellitus, HL= Hyperlipidemia, MAIN COMPARE: Revascularization for Unprotected Left Main Coronary Artery Stenosis: Comparison of Percutaneous Coronary Angioplasty versus Surgical Revascularization. DELTA: Drug-eluting stent for left main coronary artery disease. CUSTOMIZE: The Appraise a Customized Strategy for Left Main Revascularization. Credo-KYOTO: Coronary REvascularization Demonstrating Outcome Study in Kyoto
-Not available or unable to extract, * Age presented as - mean PCI - mean CABG (%) = Percentages, FU: Follow up period

S. Table 8

Pooled outcomes of PCI vs. CABG, based on follow up durations across RCTs.

Event	30 days	1 year	5 year	10 year
**MACCE**	**0.61 (0.47-0.79, p=0.0002)**	**1.07 (1.93-1.23, p= 0.33)**	**1.31(1.19-1.45, p<0.00001)**	**0.68 (0.44-1.06, p=0.09)**
**MI**	**0.76 (0.54-1.05, P=0.10)**	**1.06 (0.72-1.57, P=0.76)**	**1.62 (1.00-2.62, P=0.05)**	**1.21 (0.67-2.18, P=0.53)**
**Revascularization**	**0.64 (0.34-1.20, P=0.17)**	**1.71 (1.39-2.10, P=<0.00001)**	**1.72 (1.47-2.00, P=<0.00001)**	**2.95 (0.22-39.28, P=0.41)**
**Stroke**	**0.41 (0.17-0.98, P=0.05)**	**0.32 (0.15-0.69, P= 0.004)**	**0.86 (0.44-1.68, P=0.66)**	**0.68 (0.28-1.65, P=0.39)**
**Mortality**	**0.58(0.30-1.16, P=0.12)**	**0.83(0.63-1.07, P=0.15)**	**1.07 (0.84-1.36, P=0.57)**	**0.79 (0.60-1.05, P=0.10)**

MI: Myocardial Infarction, MACCE: Major Adverse Cardiovascular and Cerebrovascular events.

S. Table 9

Pooled results of PCI vs. CABG; based on follow up durations across observational studies.

Follow Up	MACCE	MI	Revascularization	Stroke	Mortality
**IH**	**0.27 (0.20-0.36, p <0.00001)**	**0.41 (0.12-0.95, p= 0.04)**	**1.49 (0.63-3.52, p= 0.36)**	**0.20 (0.08-0.49, p= 0.0004)**	**0.40 (0.19-0.87, p= 0.02)**
**30 days**	**0.52 (0.29-0.94, p= 0.03)**	**1.02 (0.58-1.81, p= 0.94)**	**0.69 (0.26-1.86, p=0.46)**	**0.34 (0.13-0.87, p= 0.02)**	**0.52 (0.23-1.18, p=0.12)**
**6 months**	**1.16 (0.62-2.16, p= 0.64)**	**6.02 (2.00-18.06, p= 0.001)**	**1.92 (0.67-5.49, p= 0.22)**	**0.53 (0.19-1.54, p= 0.24)**	**1.92 (0.59-6.23, p= 0.28)**
**1 year**	**1.61 (1.30-2.00, p <0.0001)**	**1.47 (1.12-1.92, p=0.005)**	**1.69 (1.04-4.47, p <0.00001)**	**0.55 (0.19-0.79, p= 0.001)**	**1.04 (0.78-.138, p= 0.79)**
**3 year**	**0.96 (0.64-1.43, p= 0.85)**	**1.71 (1.23-2.38, p= 0.001)**	**5.08 (4.02-6.40, p <0.00001)**	**0.29 (0.19-0.45, p <0.00001)**	**0.92 (0.54-1.54, p= 0.74)**
**5 year**	**1.82 (1.04-3.21, p=0.04)**	**1.74 (1.37-2.22, p <0.00001)**	**1.48 (2.57-4.71, p <0.00001)**	**0.49 (0.28-0.84, p= 0.009)**	**0.92 (0.69-1.23, p=0.59)**
**10 year**	**0.68 (0.44-1.06, p= 0.09)**	**1.21 (0.67-2.18, p= 0.53)**	**2.95 (0.22-39.28, p= 0.41)**	**0.68 (0.28-1.65, p= 0.39)**	**0.79 (0.60-1.05, p= 0.10)**

Abbreviations: IH: In-hospital, MI: Myocardial Infarction, MACCE: Major Adverse Cardiovascular and Cerebrovascular events

S. Table 10

Results and Limitations of previous meta-analyses on PCI vs. CABG for LMCAD.

Author/Year/Reference	Studies	Limitations	Follow-up	MACCE
De Rosa 2018 [49]	5	No subgroup analyses, Variation in the definition of the primary end points	5 years	1.33 (1.12-1.58)
Kodumuri 2018 [50]	12	No subgroup analyses, Variation in the definition of the primary end points,	5 years	1.23 (1.01–1.51)
Bittl 2018 [51]	12	No subgroup analyses, Variation in the definition of the primary end points,	1 year	1.00 (0.72-1.40)
Bajaj 2018 [52]	6	No subgroup analyses, Variation in the definition of the primary end points,	1 year	1.21 (1.05-1.40)
Upadhaya 2018 [53]	5	Variation in the definition of the primary end points, follow-up data for only 1-3 years. Less population	1 year	1.36 (1.18-1.58)
Khan 2017 [54]	6	Unexplored heterogeneities with regards to study design, patient characteristics, methods employed, types of stents used	5 years	1.32 (1.13–1.53)
Zhang 2017 [55]	28	Selective outcome reporting was observed in a number of observational studies, and publication bias	1 year	1.42 (1.14-1.77)
Kajimoto 2017 [56]	3	Follow-up period was limited to one year, Less population	1 year	0.40 (0.29 to 0.55)
Jang 2017 [57]	12	No subgroup analyses, Variation in the definition of the primary end points,	1 year	0.70 (0.49 to 1.00)
Tamburino 2017 [58]		Lower population and confounding variables.	2 years	
Alam 2017 [59]	10	Limited data on the medical therapies, no long-term follow-up	1 year	0.82 (0.47-1.41)
Capodanno 2017 [60]	4	Variation in study design, endpoint definitions, and possible publication bias	1 year	1.276 (0.950–1.715)
Biondi-Zoccai 2017 [61]	17	No subgroup analyses, Variation in the definition of the primary end points,	1 year	2.5 (1.2-3.8)
Palmerini 2017 [62]	19	No subgroup analysis on Syntax data, shorter duration, less patients	30 days	2.94 (1.69 to 5.09)
Ye 2017 [63]	6	Variation in revascularization and myocardial infarction were not resolved	1 year	1.21 (1.05-1.40)
Ali 2017 [64]	29	Lack of bias assessment, High selection bias	5 years	1.22 (0.95–1.56)
Naik 2016 [65]	10	Short duration of follow up. Variable study designs and publication bias	1 year	0.82 (0.62–1.09)
Lee 2015 [66]	8	Short length of follow up duration; non-availability of clinical event rates on anatomical basis	1 year	1.25 (0.86-1.82)
Alam 2014 [67]	27	Lack of longer length of follow up; not all studies reported rates of stent thrombosis or cardiac death	5 years	1.30 (1.10–1.55)
Athappan 2013 [68]	24	Variable definitions of endpoints by primary studies	5 years	0.64 (0.51-0.803)
Sa 2013 [69]	16	Short length of follow ups in studies	1 year	1.607
Ali 2013 [70]	29	Short follow-up; Risk of selection bias, variable definition of MI	5 years	1.22 (0.95-1.56)
Lee CW 2012 [71]	3	Definition of clinical outcome was different across trials; Imbalance drug use	5 years	0.46 (0.33–0.64)
Khan 2012 [72]	9	No subgroup analyses based on DES, Variation in the definition of the primary end points and follow ups	5 years	1.19 (0.93-1.54)
Testa 2012 [73]	6	No subgroup analyses based on DES, Variation in the definition of the primary end points and follow ups	5 years	1.02 (0.76-1.38)
Putzu 2012 [74]	5	Variability in nature of the two treatments; individual patient data could not be explored	5 years	0.55 (0.45-0.67)
Sharma 2012 [75]	6	No subgroup analyses based on DES, Variation in the definition of the primary end points and follow ups	5 years	1.79 (1.22-2.64)
Spinthakis 2012 [76]	5	No subgroup analyses based on DES, Variation in the definition of the primary end points and follow ups	5 years	2.04 (1.30-3.19)
Moore 2011 [77]	4	No subgroup analyses based on DES, Variation in the definition of the primary end points and follow ups	5 years	1.37 (1.18-1.58)
Garg 2011 [78]	5	No subgroups on SYNTAX score	5 years	1.45 (0.87-2.40)
Shah 2010 [79]	8	No subgroup analyses based on DES, Variation in the definition of the primary end points and follow ups	5 years	1.20 (1.03-1.40)
Mahmoud 2010 [80]	6	No subgroup analyses based on DES, Variation in the definition of the primary end points and follow ups	5 years	1.19 (1.01-1.41)
Khan AR 2009 [81]	8	No subgroup analyses, Variation in the definition of the primary end points,	5 years	1.16 (0.95-1.43)
Nerlekar 2008 [82]	5	No long-term data; variable definition of repeat revascularization, No subgroup analyses on DES	5 years	1.46 (0.88-2.45)
Giacoppo 2017 [83]	4	No subgroup analyses, Variation in the definition of the primary end points,	5 years	1.06 (0.85-1.32)
Palmerini 2012 [84]	6	Differences in baseline characteristics, variation in definition of clinical end	5 years	1.33 (0.84-2.11)

		**points across trials, short median follow up (39 months)**		
**Wang 2019 [85]**	**19**	**No subgroup analyses, Variation in the definition of the primary end points,**	**5 years**	**1.89 (1.48-2.40)**
**Ahmad 2020 [86)**	**5**	**No comparison of MACCE**	**5 years**	**-------**

D. SUPPLEMENTAL FOREST PLOTS FOR ALL STUDIES:

A. All 30 day MACCE

Study or Subgroup	PCI Events	PCI Total	CABG Events	CABG Total	Weight	Odds Ratio M-H, Random, 95% CI
Buszman (LE MANS) 2008	1	52	7	53	1.8%	0.13 [0.02, 1.09]
Sanmartin 2007	2	96	22	245	3.7%	0.22 [0.05, 0.94]
Park (PRECOMBAT) 2011	4	300	9	300	5.3%	0.44 [0.13, 1.43]
Wu 2010	4	131	14	245	5.8%	0.52 [0.17, 1.61]
Qin 2013	4	233	14	282	5.8%	0.33 [0.11, 1.03]
LU 2016	4	208	18	270	6.1%	0.27 [0.09, 0.82]
Boudriot 2011	6	100	10	101	6.5%	0.58 [0.20, 1.66]
Kang 2010	13	205	10	257	9.2%	1.67 [0.72, 3.90]
Rodes Cabau 2008	19	104	40	145	14.3%	0.59 [0.32, 1.09]
Makikallio (NOBLE) 2016	28	592	37	592	17.9%	0.74 [0.45, 1.23]
Stone (EXCEL) 2019	46	948	80	957	23.4%	0.56 [0.38, 0.81]
**Total (95% CI)**		**2969**		**3447**	**100.0%**	**0.56 [0.42, 0.76]**
Total events	131		261			

Heterogeneity: Tau² = 0.06; Chi² = 13.70, df = 10 (P = 0.19); I² = 27%
Test for overall effect: Z = 3.79 (P = 0.0002)

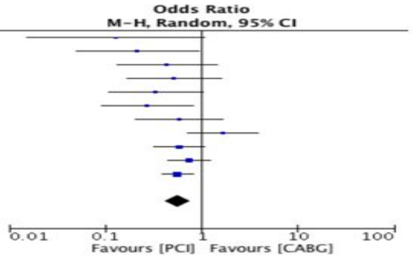

B. All 30 day MI

Study or Subgroup	PCI Events	PCI Total	CABG Events	CABG Total	Weight	Odds Ratio M-H, Random, 95% CI
Sanmartin 2007	0	96	3	245	1.0%	0.36 [0.02, 7.02]
LU 2016	1	208	1	270	1.1%	1.30 [0.08, 20.90]
Buszman (LE MANS) 2008	1	52	2	53	1.5%	0.50 [0.04, 5.69]
Qin 2013	2	233	1	282	1.5%	2.43 [0.22, 27.00]
Kang 2010	3	205	1	257	1.7%	3.80 [0.39, 36.83]
Boudriot 2011	3	100	3	101	3.3%	1.01 [0.20, 5.13]
Palmerini 2006	7	157	3	154	4.7%	2.35 [0.60, 9.26]
Rodes Cabau 2008	13	104	25	145	16.7%	0.69 [0.33, 1.41]
Makikallio (NOBLE) 2016	19	592	16	592	19.2%	1.19 [0.61, 2.34]
Stone (EXCEL) 2019	37	948	59	957	49.3%	0.62 [0.41, 0.94]
**Total (95% CI)**		**2695**		**3056**	**100.0%**	**0.81 [0.60, 1.09]**
Total events	86		114			

Heterogeneity: Tau² = 0.00; Chi² = 8.58, df = 9 (P = 0.48); I² = 0%
Test for overall effect: Z = 1.37 (P = 0.17)

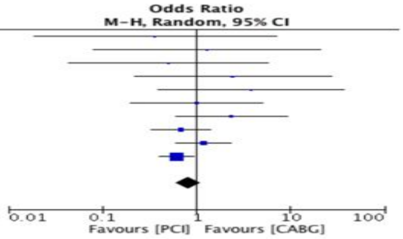

C. All 30 day Revascularization

Study or Subgroup	PCI Events	PCI Total	CABG Events	CABG Total	Weight	Odds Ratio M-H, Random, 95% CI
Wu 2010	0	131	0	245		Not estimable
Buszman (LE MANS) 2008	1	52	0	53	2.7%	3.12 [0.12, 78.27]
LU 2016	0	208	1	270	2.8%	0.43 [0.02, 10.63]
Qin 2013	0	233	1	282	2.8%	0.40 [0.02, 9.91]
Sanmartin 2007	0	96	2	245	3.1%	0.50 [0.02, 10.61]
Palmerini 2006	1	157	1	154	3.7%	0.98 [0.06, 15.82]
Boudriot 2011	1	100	2	101	4.9%	0.50 [0.04, 5.60]
Rodes Cabau 2008	1	104	4	145	5.8%	0.34 [0.04, 3.11]
Kang 2010	3	205	3	257	10.9%	1.26 [0.25, 6.30]
Makikallio (NOBLE) 2016	7	592	10	592	30.0%	0.70 [0.26, 1.84]
Stone (EXCEL) 2019	7	948	13	957	33.3%	0.54 [0.21, 1.36]
**Total (95% CI)**		**2826**		**3301**	**100.0%**	**0.65 [0.38, 1.11]**
Total events	21		37			

Heterogeneity: Tau² = 0.00; Chi² = 2.36, df = 9 (P = 0.98); I² = 0%
Test for overall effect: Z = 1.56 (P = 0.12)

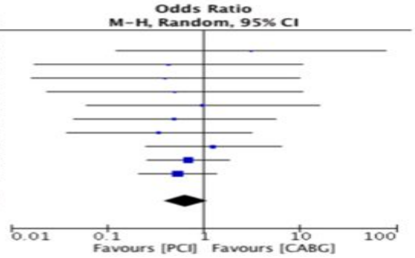

D. All 30 day Stroke

Study or Subgroup	PCI Events	PCI Total	CABG Events	CABG Total	Weight	Odds Ratio M-H, Random, 95% CI
Buszman (LE MANS) 2008	0	52	2	53	4.5%	0.20 [0.01, 4.19]
Sanmartin 2007	0	96	2	245	4.6%	0.50 [0.02, 10.61]
Makikallio (NOBLE) 2016	0	592	4	592	4.9%	0.11 [0.01, 2.05]
Qin 2013	1	233	3	282	8.2%	0.40 [0.04, 3.88]
LU 2016	1	208	5	270	9.1%	0.26 [0.03, 2.21]
Rodes Cabau 2008	1	104	8	145	9.6%	0.17 [0.02, 1.35]
Kang 2010	2	205	5	257	15.5%	0.50 [0.10, 2.59]
Stone (EXCEL) 2019	6	948	12	957	43.6%	0.50 [0.19, 1.34]
**Total (95% CI)**		**2438**		**2801**	**100.0%**	**0.37 [0.19, 0.71]**
Total events	11		41			

Heterogeneity: Tau² = 0.00; Chi² = 2.08, df = 7 (P = 0.96); I² = 0%
Test for overall effect: Z = 3.00 (P = 0.003)

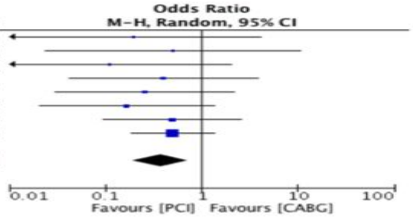

E. All 30 day all-cause Mortality

Study or Subgroup	PCI Events	PCI Total	CABG Events	CABG Total	Weight	Odds Ratio M-H, Random, 95% CI
Buszman (LE MANS) 2008	0	52	2	53	2.9%	0.20 [0.01, 4.19]
Wu 2010	1	131	8	245	5.4%	0.23 [0.03, 1.84]
Boudriot 2011	2	100	5	101	7.5%	0.39 [0.07, 2.07]
Makikallio (NOBLE) 2016	2	592	7	592	8.1%	0.28 [0.06, 1.37]
Sanmartin 2007	2	96	15	245	8.6%	0.33 [0.07, 1.45]
Qin 2013	2	233	20	282	8.8%	0.11 [0.03, 0.49]
Kang 2010	9	205	3	257	9.9%	3.89 [1.04, 14.55]
LU 2016	3	98	14	161	10.3%	0.33 [0.09, 1.18]
Palmerini 2006	5	157	7	154	11.2%	0.69 [0.21, 2.23]
Rodes Cabau 2008	7	104	12	145	13.3%	0.80 [0.30, 2.11]
Stone (EXCEL) 2019	9	948	10	957	13.9%	0.91 [0.37, 2.24]
**Total (95% CI)**		**2716**		**3192**	**100.0%**	**0.52 [0.30, 0.91]**
Total events	42		103			

Heterogeneity: Tau² = 0.38; Chi² = 17.97, df = 10 (P = 0.06); I² = 44%
Test for overall effect: Z = 2.27 (P = 0.02)

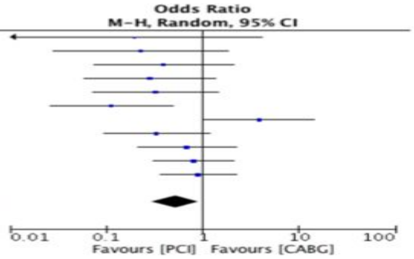

S. Figure 1: Forest Plots showing an individual and pooled OR for all-studies comparing PCI to CABG for LMCAD at 30-days (a. MACCE b. MI c. revascularization d. stroke e. death).

A. All 1-year MACCE

Study or Subgroup	PCI Events	PCI Total	CABG Events	CABG Total	Weight	Odds Ratio M-H, Random, 95% CI
HSU 2008	1	20	13	39	0.7%	0.11 [0.01, 0.88]
Yin 2015	4	101	4	121	1.4%	1.21 [0.29, 4.95]
White 2008	15	67	10	67	2.9%	1.64 [0.68, 3.98]
Buszman (LE MANS) 2008	16	52	13	53	3.0%	1.37 [0.58, 3.23]
Zhao 2011	13	55	14	108	3.1%	2.08 [0.90, 4.80]
Ghenim 2009	15	101	14	101	3.3%	1.08 [0.49, 2.38]
Sanmartin 2007	10	96	27	245	3.4%	0.94 [0.44, 2.02]
Shimizu 2010	20	64	16	89	3.5%	2.07 [0.97, 4.42]
Boudriot 2011	19	100	14	101	3.5%	1.46 [0.69, 3.10]
Rittger 2011	14	95	26	192	3.8%	1.10 [0.55, 2.23]
Kawecki 2012	24	88	18	111	3.9%	1.94 [0.97, 3.86]
Yi Gijong 2012	39	128	17	128	4.2%	2.86 [1.52, 5.39]
Park (PRECOMBAT) 2011	26	300	20	300	4.4%	1.33 [0.72, 2.44]
Caggegi (CUSTOMIZE) 2011	32	222	19	361	4.5%	3.03 [1.67, 5.49]
Rodes Cabau 2008	45	104	51	145	5.1%	1.41 [0.84, 2.36]
Qin 2013	29	233	46	282	5.2%	0.73 [0.44, 1.20]
Kang 2010	49	205	32	257	5.3%	2.21 [1.35, 3.61]
Wu 2010	35	131	55	245	5.3%	1.26 [0.77, 2.06]
Makikallio (NOBLE) 2016	42	592	42	592	5.7%	1.00 [0.64, 1.56]
Buchanan 2014	149	489	51	328	6.4%	2.38 [1.67, 3.40]
Serruys (SYNTAX) 2009	111	357	96	348	6.7%	1.18 [0.86, 1.64]
Stone (EXCEL) 2019	129	948	136	957	7.2%	0.95 [0.73, 1.23]
Chieffo 2012	659	1874	205	900	7.8%	1.84 [1.53, 2.21]
**Total (95% CI)**		**6422**		**6070**	**100.0%**	**1.45 [1.21, 1.75]**
Total events	1496		939			

Heterogeneity: $Tau^2 = 0.10$; $Chi^2 = 59.57$, df = 22 (P < 0.0001); $I^2 = 63\%$
Test for overall effect: Z = 3.98 (P < 0.0001)

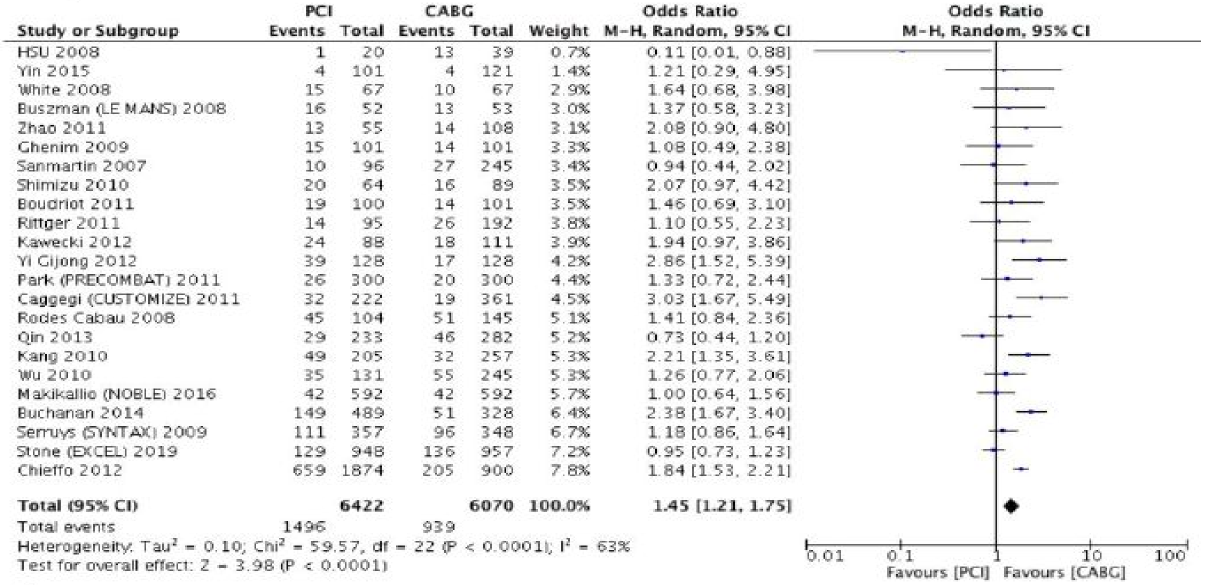

B. All 1-year MI

Study or Subgroup	PCI Events	PCI Total	CABG Events	CABG Total	Weight	Odds Ratio M-H, Random, 95% CI
HSU 2008	0	20	0	39		Not estimable
Yin 2015	0	106	1	121	0.6%	0.38 [0.02, 9.36]
Shimizu 2010	2	64	0	89	0.6%	7.16 [0.34, 151.72]
Yi Gijong 2012	3	128	0	128	0.7%	7.17 [0.37, 140.18]
Sanmartin 2007	0	96	3	245	0.7%	0.36 [0.02, 7.02]
Rittger 2011	2	95	1	192	1.0%	4.11 [0.37, 45.88]
Buszman (LE MANS) 2008	1	52	3	53	1.1%	0.33 [0.03, 3.25]
Caggegi (CUSTOMIZE) 2011	4	222	2	361	1.9%	3.29 [0.60, 18.13]
Boudriot 2011	3	100	3	101	2.1%	1.01 [0.20, 5.13]
Qin 2013	3	233	3	282	2.2%	1.21 [0.24, 6.07]
Park (PRECOMBAT) 2011	4	300	3	300	2.5%	1.34 [0.30, 6.03]
Kang 2010	5	205	3	257	2.7%	2.12 [0.50, 8.96]
Kawecki 2012	10	88	4	111	3.7%	3.43 [1.04, 11.34]
Zhao 2011	6	55	9	108	4.4%	1.35 [0.45, 4.00]
Buchanan 2014	21	489	5	328	5.3%	2.90 [1.08, 7.77]
Palmerini 2006	13	157	7	154	5.6%	1.90 [0.74, 4.89]
Makikallio (NOBLE) 2016	11	592	8	592	5.9%	1.38 [0.55, 3.46]
Serruys (SYNTAX) 2009	28	357	16	348	10.6%	1.77 [0.94, 3.33]
Rodes Cabau 2008	24	104	28	145	11.0%	1.25 [0.68, 2.32]
Chieffo 2012	75	1874	33	900	17.7%	1.10 [0.72, 1.66]
Stone (EXCEL) 2019	53	948	69	957	19.8%	0.76 [0.53, 1.10]
**Total (95% CI)**		**6285**		**5811**	**100.0%**	**1.33 [1.04, 1.70]**
Total events	268		201			

Heterogeneity: $Tau^2 = 0.04$; $Chi^2 = 22.57$, df = 19 (P = 0.26); $I^2 = 16\%$
Test for overall effect: Z = 2.30 (P = 0.02)

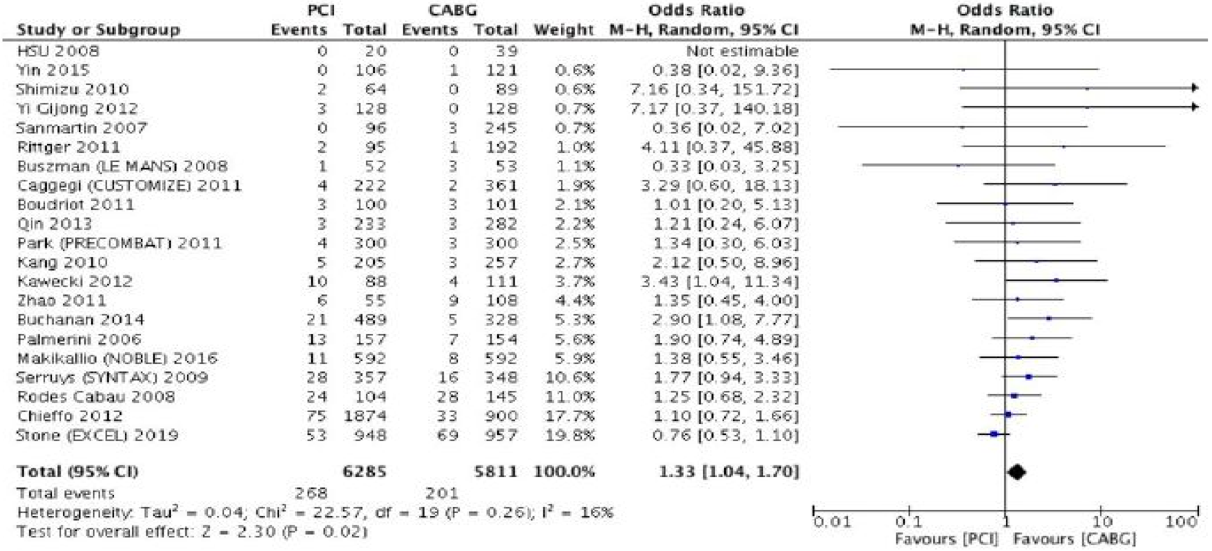

C. All 1-year Revascularization

Study or Subgroup	PCI Events	PCI Total	CABG Events	CABG Total	Weight	Odds Ratio M-H, Random, 95% CI
HSU 2008	0	20	4	39	0.6%	0.19 [0.01, 3.76]
Yin 2015	4	106	1	121	1.0%	4.71 [0.52, 42.78]
Ghenim 2009	14	101	1	101	1.1%	16.09 [2.07, 124.88]
Sanmartin 2007	5	96	2	245	1.6%	6.68 [1.27, 35.02]
Rittger 2011	10	95	3	192	2.4%	7.41 [1.99, 27.62]
Zhao 2011	8	55	4	108	2.6%	4.43 [1.27, 15.43]
Yi Gijong 2012	19	128	4	128	3.1%	5.40 [1.78, 16.37]
Buszman (LE MANS) 2008	15	52	5	53	3.1%	3.89 [1.30, 11.68]
Rodes Cabau 2008	10	104	7	145	3.5%	2.10 [0.77, 5.71]
Boudriot 2011	14	100	6	101	3.5%	2.58 [0.95, 7.01]
Kim 2009	28	251	5	256	3.7%	6.30 [2.39, 16.60]
Caggegi (CUSTOMIZE) 2011	18	222	6	361	3.8%	5.22 [2.04, 13.36]
Shimizu 2010	17	64	10	89	4.3%	2.86 [1.21, 6.76]
Qin 2013	17	233	9	282	4.5%	2.39 [1.04, 5.46]
Wu 2008	37	135	8	135	4.6%	5.99 [2.67, 13.45]
Park (PRECOMBAT) 2011	18	300	10	300	4.7%	1.85 [0.84, 4.08]
Kang 2010	35	205	10	257	5.2%	5.09 [2.45, 10.55]
Wu 2010	24	131	23	245	6.1%	2.16 [1.17, 4.01]
Makikallio (NOBLE) 2016	32	592	24	592	6.8%	1.35 [0.79, 2.33]
Buchanan 2014	124	489	29	328	7.9%	3.50 [2.27, 5.40]
Stone (EXCEL) 2019	65	948	41	957	8.2%	1.64 [1.10, 2.46]
Serruys (SYNTAX) 2009	83	357	46	348	8.2%	1.99 [1.34, 2.95]
Chieffo 2012	482	1874	80	900	9.6%	3.55 [2.76, 4.57]
**Total (95% CI)**		**6658**		**6283**	**100.0%**	**3.01 [2.40, 3.79]**
Total events	1079		338			

Heterogeneity: $Tau^2 = 0.12$; $Chi^2 = 45.18$, df = 22 (P = 0.003); $I^2 = 51\%$
Test for overall effect: Z = 9.46 (P < 0.00001)

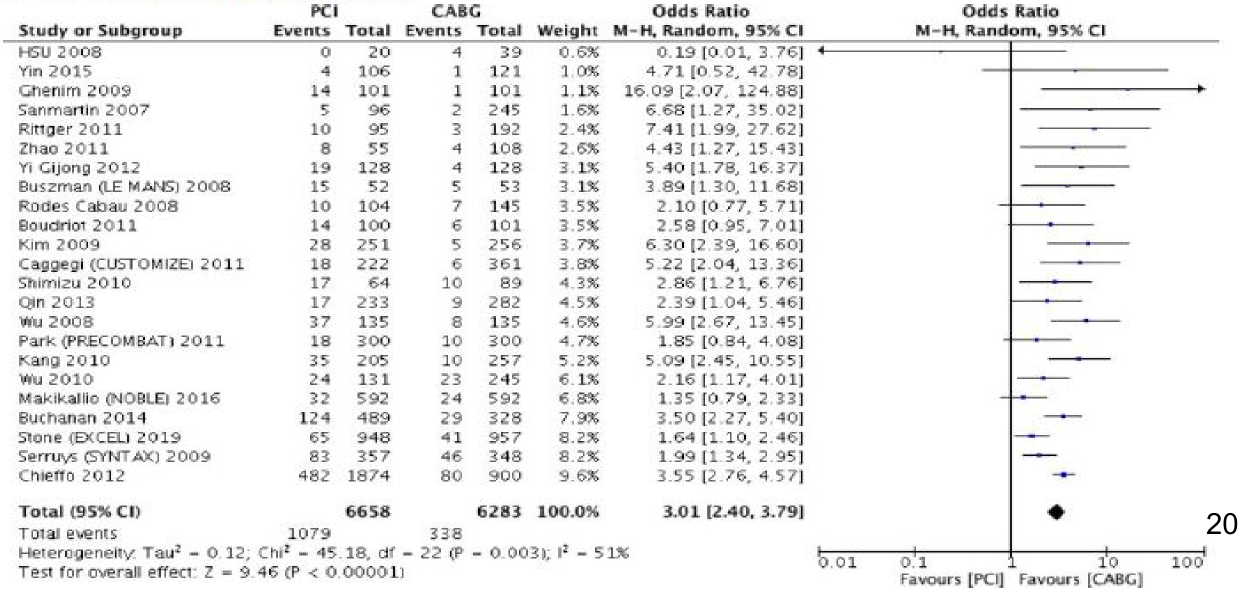

D. All 1-year Stroke

Study or Subgroup	PCI Events	PCI Total	CABG Events	CABG Total	Weight	Odds Ratio M-H, Random, 95% CI	Year
Sanmartin 2007	0	96	2	245	1.0%	0.50 [0.02, 10.61]	2007
HSU 2008	0	20	1	39	0.9%	0.63 [0.02, 16.07]	2008
Rodes Cabau 2008	9	104	9	145	10.2%	1.43 [0.55, 3.74]	2008
Buszman (LE MANS) 2008	0	52	2	53	1.0%	0.20 [0.01, 4.19]	2008
Serruys (SYNTAX) 2009	2	357	19	348	4.4%	0.10 [0.02, 0.42]	2009
Kang 2010	2	205	8	257	3.9%	0.31 [0.06, 1.46]	2010
Shimizu 2010	0	64	1	89	0.9%	0.46 [0.02, 11.41]	2010
Zhao 2011	0	55	6	108	1.1%	0.14 [0.01, 2.57]	2011
Rittger 2011	1	95	13	192	2.2%	0.15 [0.02, 1.14]	2011
Boudriot 2011	0	0	0	0		Not estimable	2011
Park (PRECOMBAT) 2011	0	300	2	300	1.0%	0.20 [0.01, 4.16]	2011
Chieffo 2012	30	1874	25	900	32.6%	0.57 [0.33, 0.97]	2012
Kawecki 2012	0	88	2	111	1.0%	0.25 [0.01, 5.22]	2012
Yi Gijong 2012	4	128	6	128	5.6%	0.66 [0.18, 2.38]	2012
Qin 2013	7	233	18	282	11.8%	0.45 [0.19, 1.11]	2013
Yin 2015	0	106	0	121		Not estimable	2015
Wei 2016	1	64	4	62	1.9%	0.23 [0.02, 2.12]	2016
Makikallio (NOBLE) 2016	2	592	6	592	3.6%	0.33 [0.07, 1.65]	2016
Stone (EXCEL) 2019	11	948	19	957	16.8%	0.58 [0.27, 1.22]	2019
**Total (95% CI)**		**5381**		**4929**	**100.0%**	**0.50 [0.37, 0.67]**	
Total events	69		143				

Heterogeneity: $Tau^2 = 0.00$; $Chi^2 = 14.55$, df = 16 (P = 0.56); $I^2 = 0\%$
Test for overall effect: Z = 4.49 (P < 0.00001)

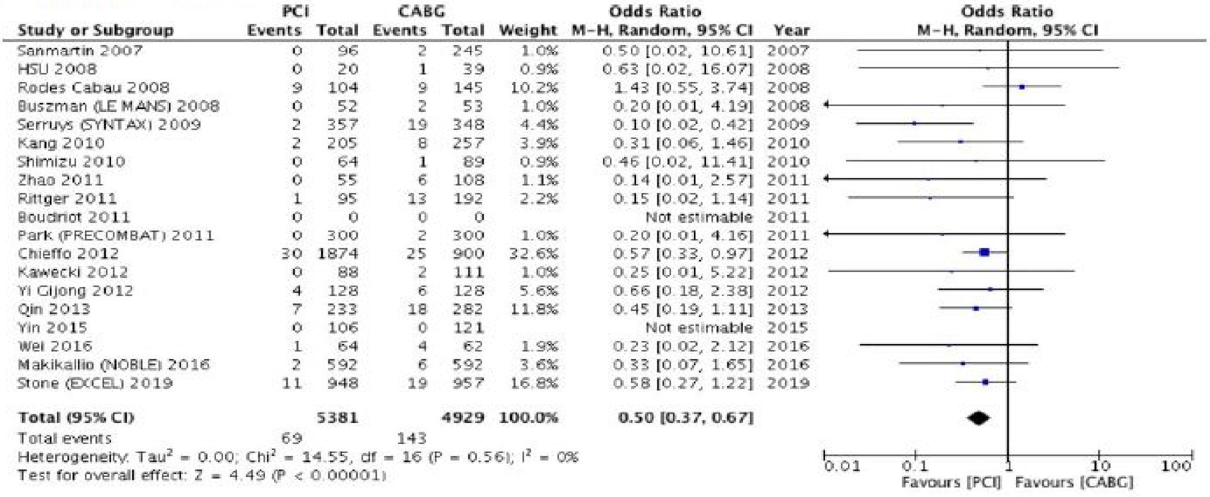

E. All 1-year all-cause Mortality

Study or Subgroup	PCI Events	PCI Total	CABG Events	CABG Total	Weight	Odds Ratio M-H, Random, 95% CI
Yin 2015	0	106	2	121	0.6%	0.22 [0.01, 4.73]
Buszman (LE MANS) 2008	1	52	4	53	1.0%	0.24 [0.03, 2.23]
HSU 2008	1	20	8	39	1.0%	0.20 [0.02, 1.76]
Boudriot 2011	2	100	5	101	1.6%	0.39 [0.07, 2.07]
Zhao 2011	3	55	7	108	2.1%	0.83 [0.21, 3.35]
Shimizu 2010	5	64	6	89	2.6%	1.17 [0.34, 4.02]
Rittger 2011	4	95	14	192	2.9%	0.56 [0.18, 1.75]
White 2008	8	67	6	67	2.9%	1.38 [0.45, 4.21]
Park (PRECOMBAT) 2011	6	300	8	300	3.1%	0.74 [0.26, 2.17]
Wei 2016	6	64	11	62	3.1%	0.48 [0.17, 1.39]
Sanmartin 2007	5	90	20	245	3.3%	0.66 [0.24, 1.82]
Qin 2013	5	233	31	282	3.6%	0.18 [0.07, 0.46]
Wu 2010	6	131	23	245	3.7%	0.46 [0.18, 1.17]
Yi Gijong 2012	16	128	9	128	4.1%	1.89 [0.80, 4.45]
Wu 2008	23	135	8	135	4.1%	3.26 [1.40, 7.58]
Kawecki 2012	14	88	12	111	4.2%	1.56 [0.68, 3.57]
Makikallio (NOBLE) 2016	9	592	17	592	4.3%	0.52 [0.23, 1.18]
Caggegi (CUSTOMIZE) 2011	14	222	13	361	4.5%	1.80 [0.83, 3.91]
Kang 2010	14	205	16	257	4.7%	1.10 [0.53, 2.32]
Rodes Cabau 2008	17	104	18	145	4.8%	1.38 [0.67, 2.82]
Kim 2009	16	251	22	256	5.1%	0.72 [0.37, 1.41]
Palmerini 2006	21	157	19	154	5.2%	1.10 [0.56, 2.13]
Stone (EXCEL) 2019	31	948	33	957	6.3%	0.95 [0.57, 1.56]
Buchanan 2014	69	489	23	328	6.3%	2.18 [1.33, 3.57]
Serruys (SYNTAX) 2009	45	357	48	348	6.8%	0.90 [0.58, 1.39]
Chieffo 2012	264	1874	103	900	8.1%	1.27 [1.00, 1.62]
**Total (95% CI)**		**6927**		**6576**	**100.0%**	**0.96 [0.76, 1.22]**
Total events	605		486			

Heterogeneity: $Tau^2 = 0.16$; $Chi^2 = 54.46$, df = 25 (P = 0.0006); $I^2 = 54\%$
Test for overall effect: Z = 0.32 (P = 0.75)

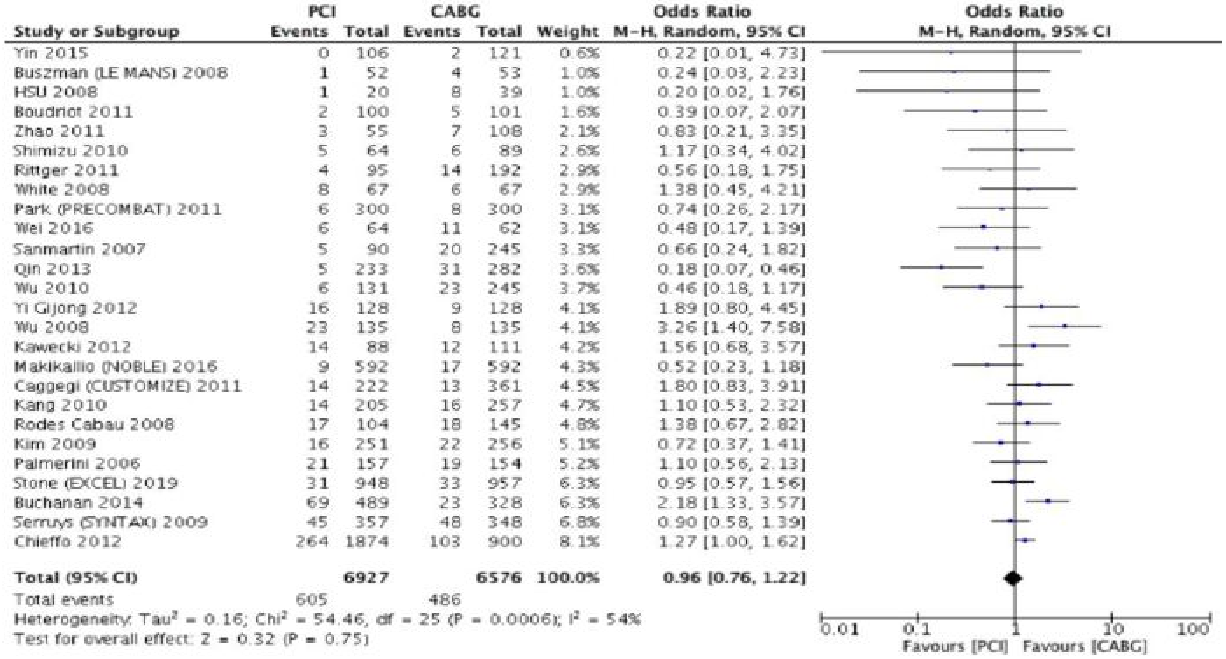

S. Figure 2: Forest Plots showing an individual and pooled OR for all-studies comparing PCI to CABG for LMCAD at 1-year (a. MACCE b. MI c. revascularization d. stroke e. death).

A. All 5-year MACCE

Study or Subgroup	PCI Events	PCI Total	CABG Events	CABG Total	Weight	Odds Ratio M–H, Random, 95% CI
Brener 2008	7	97	12	190	5.2%	1.15 [0.44, 3.03]
Park (MAIN COMPARE) 2011	149	784	7	690	6.2%	22.89 [10.65, 49.23]
Park DW 2010	17	176	41	219	6.9%	0.46 [0.25, 0.85]
Ahn (PRECOMBAT) 2015	52	300	42	300	7.7%	1.29 [0.83, 2.00]
Chang 2012	63	556	51	309	7.9%	0.65 [0.43, 0.96]
Shiomi (CREDO KYOTO) 2015	121	364	47	640	8.0%	6.28 [4.35, 9.08]
LU 2016	91	208	114	270	8.0%	1.06 [0.74, 1.53]
Delta Registry 2014	149	489	51	328	8.1%	2.38 [1.67, 3.40]
Morice (SYNTAX) 2014	130	357	103	348	8.2%	1.36 [0.99, 1.87]
Holm (NOBLE) 2020	165	592	110	592	8.4%	1.69 [1.29, 2.23]
Yu 2016	232	465	178	457	8.4%	1.56 [1.20, 2.03]
Cavalcante 2016	186	657	149	648	8.4%	1.32 [1.03, 1.70]
Stone (EXCEL) 2019	290	948	228	957	8.6%	1.41 [1.15, 1.73]
**Total (95% CI)**		**5993**		**5948**	**100.0%**	**1.67 [1.18, 2.36]**
Total events	1652		1133			

Heterogeneity: $Tau^2 = 0.35$; $Chi^2 = 153.36$, df = 12 ($P < 0.00001$); $I^2 = 92\%$
Test for overall effect: Z = 2.92 (P = 0.004)

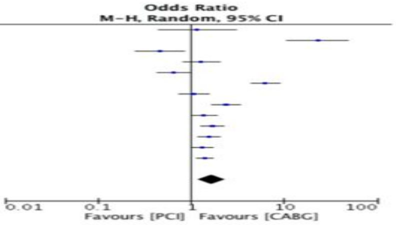

B. All 5-year MI

Study or Subgroup	PCI Events	PCI Total	CABG Events	CABG Total	Weight	Odds Ratio M–H, Random, 95% CI
Chang 2012	5	556	3	309	2.1%	0.93 [0.22, 3.90]
Ahn (PRECOMBAT) 2015	6	300	5	300	2.9%	1.20 [0.36, 3.99]
Delta Registry 2014	21	489	5	328	4.2%	2.90 [1.08, 7.77]
LU 2016	17	208	18	270	7.7%	1.25 [0.63, 2.48]
Morice (SYNTAX) 2014	28	357	16	348	8.8%	1.77 [0.94, 3.33]
Shiomi (CREDO KYOTO) 2015	25	364	19	640	9.3%	2.41 [1.31, 4.44]
Park DW 2010	27	176	21	219	9.3%	1.71 [0.93, 3.14]
Holm (NOBLE) 2020	43	592	15	592	9.5%	3.01 [1.65, 5.49]
Cavalcante 2016	34	657	21	648	10.7%	1.63 [0.94, 2.84]
Yu 2016	53	465	32	457	13.9%	1.71 [1.08, 2.70]
Stone (EXCEL) 2019	95	948	84	957	21.7%	1.16 [0.85, 1.58]
**Total (95% CI)**		**5112**		**5068**	**100.0%**	**1.67 [1.35, 2.06]**
Total events	354		239			

Heterogeneity: $Tau^2 = 0.03$; $Chi^2 = 13.16$, df = 10 ($P = 0.21$); $I^2 = 24\%$
Test for overall effect: Z = 4.71 ($P < 0.00001$)

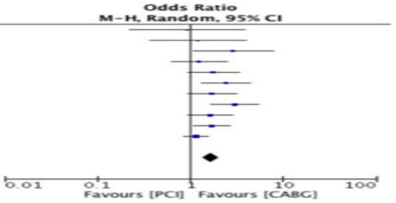

C. All 5-year Revascularization

Study or Subgroup	PCI Events	PCI Total	CABG Events	CABG Total	Weight	Odds Ratio M–H, Random, 95% CI
Park DW 2010	32	176	10	219	5.7%	4.64 [2.21, 9.75]
Chang 2012	88	556	9	309	6.0%	6.27 [3.11, 12.64]
Ahn (PRECOMBAT) 2015	38	300	21	300	7.3%	1.93 [1.10, 3.37]
Delta Registry 2014	74	489	17	328	7.4%	3.26 [1.89, 5.64]
LU 2016	47	208	31	270	7.9%	2.25 [1.37, 3.69]
Park (MAIN COMPARE) 2011	123	784	22	690	8.2%	5.65 [3.54, 9.01]
Morice (SYNTAX) 2014	90	357	49	348	9.0%	2.06 [1.40, 3.02]
Holm (NOBLE) 2020	97	592	58	592	9.4%	1.80 [1.27, 2.55]
Cavalcante 2016	128	657	70	648	9.7%	2.00 [1.46, 2.74]
Shiomi (CREDO KYOTO) 2015	148	364	84	640	9.7%	4.54 [3.32, 6.19]
Yu 2016	171	465	80	457	9.8%	2.74 [2.02, 3.72]
Stone (EXCEL) 2019	153	948	92	957	10.0%	1.81 [1.37, 2.38]
**Total (95% CI)**		**5896**		**5758**	**100.0%**	**2.80 [2.18, 3.60]**
Total events	1189		543			

Heterogeneity: $Tau^2 = 0.14$; $Chi^2 = 48.79$, df = 11 ($P < 0.00001$); $I^2 = 77\%$
Test for overall effect: Z = 8.11 ($P < 0.00001$)

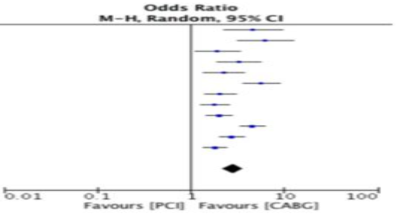

D. All 5-year Stroke

Study or Subgroup	PCI Events	PCI Total	CABG Events	CABG Total	Weight	Odds Ratio M–H, Random, 95% CI
Chang 2012	1	556	4	309	3.1%	0.14 [0.02, 1.23]
Ahn (PRECOMBAT) 2015	2	300	2	300	3.7%	1.00 [0.14, 7.15]
Park DW 2010	3	176	12	219	6.6%	0.30 [0.08, 1.08]
LU 2016	4	208	14	270	7.6%	0.36 [0.12, 1.11]
Morice (SYNTAX) 2014	5	357	14	348	8.3%	0.34 [0.12, 0.95]
Cavalcante 2016	7	657	16	648	9.4%	0.43 [0.17, 1.04]
Park (MAIN COMPARE) 2011	12	784	13	690	10.4%	0.81 [0.37, 1.79]
Holm (NOBLE) 2020	21	592	12	592	11.1%	1.78 [0.87, 3.65]
Stone (EXCEL) 2019	26	948	33	957	13.0%	0.79 [0.47, 1.33]
Shiomi (CREDO KYOTO) 2015	27	364	42	640	13.2%	1.14 [0.69, 1.88]
Yu 2016	30	465	80	457	13.7%	0.33 [0.21, 0.51]
**Total (95% CI)**		**5407**		**5430**	**100.0%**	**0.60 [0.39, 0.92]**
Total events	138		242			

Heterogeneity: $Tau^2 = 0.30$; $Chi^2 = 29.63$, df = 10 ($P = 0.0010$); $I^2 = 66\%$
Test for overall effect: Z = 2.36 (P = 0.02)

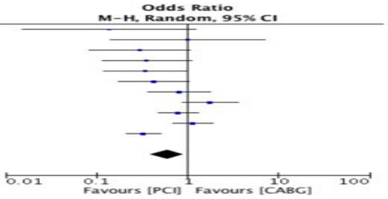

E. All 5-year all-cause Mortality

Study or Subgroup	PCI Events	PCI Total	CABG Events	CABG Total	Weight	Odds Ratio M–H, Random, 95% CI
Park DW 2010	10	176	24	219	4.8%	0.49 [0.23, 1.05]
Ahn (PRECOMBAT) 2015	17	300	23	300	5.8%	0.72 [0.38, 1.38]
Delta Registry 2014	69	489	23	328	7.4%	2.18 [1.33, 3.57]
Morice (SYNTAX) 2014	45	357	48	348	8.1%	0.90 [0.58, 1.39]
Chang 2012	57	556	45	309	8.4%	0.67 [0.44, 1.02]
Holm (NOBLE) 2020	54	592	50	592	8.6%	1.09 [0.73, 1.63]
LU 2016	57	208	85	270	8.6%	0.82 [0.55, 1.22]
Cavalcante 2016	62	657	72	648	9.2%	0.83 [0.58, 1.19]
Yu 2016	73	465	89	457	9.4%	0.77 [0.55, 1.08]
Shiomi (CREDO KYOTO) 2015	88	364	109	640	9.7%	1.55 [1.13, 2.13]
Park (MAIN COMPARE) 2011	95	784	106	690	10.0%	0.76 [0.56, 1.02]
Stone (EXCEL) 2019	119	948	89	957	10.1%	1.40 [1.05, 1.87]
**Total (95% CI)**		**5896**		**5758**	**100.0%**	**0.96 [0.78, 1.19]**
Total events	746		763			

Heterogeneity: $Tau^2 = 0.10$; $Chi^2 = 37.75$, df = 11 ($P < 0.0001$); $I^2 = 71\%$
Test for overall effect: Z = 0.35 (P = 0.72)

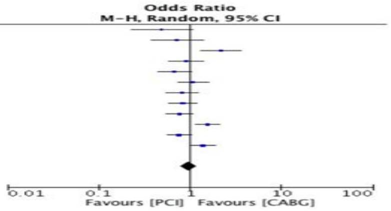

S. Figure 3: Forest Plots showing an individual and pooled OR for all-studies comparing PCI to CABG for LMCAD at 5-Year (a. MACCE b. MI c. revascularization d. stroke e. death)

A. 10-year MACCE

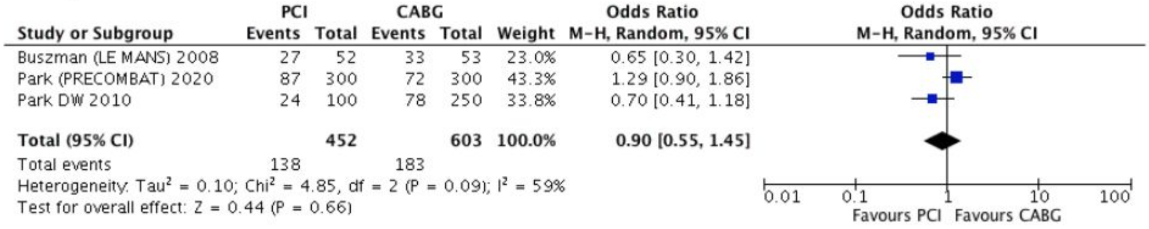

B. 10-year MI

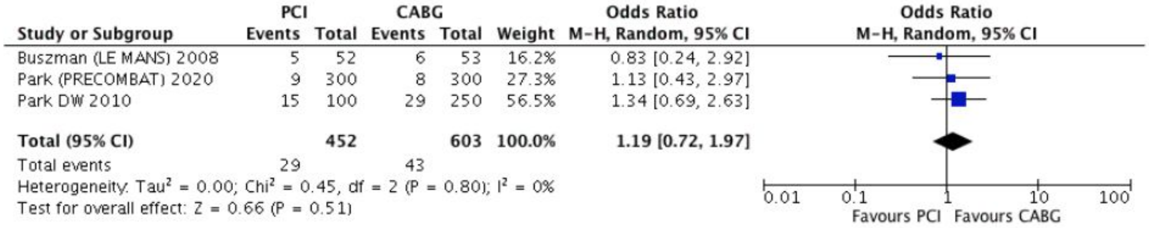

C. 10-year Revascularization

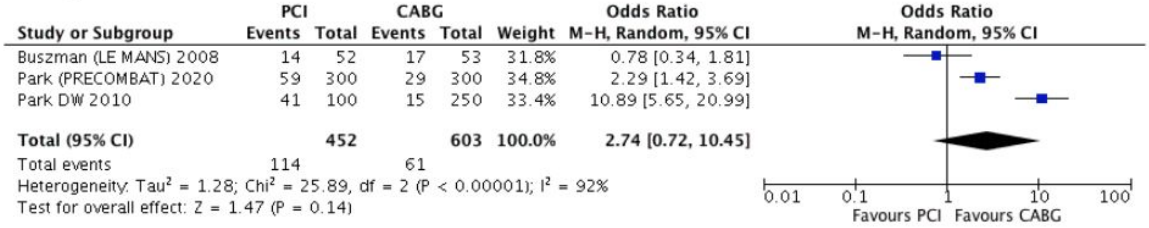

D. 10-year Stroke

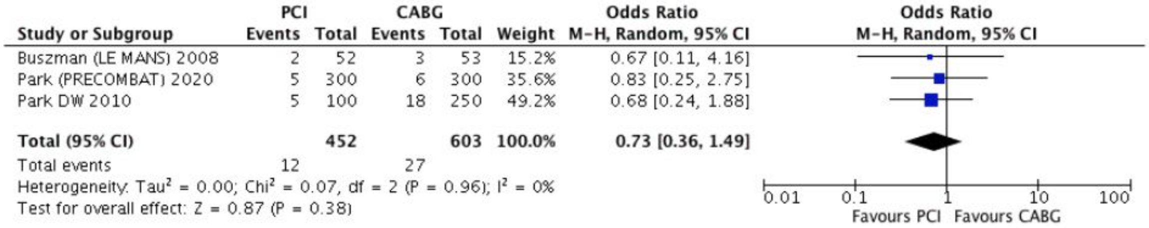

E. 10-year all-cause Mortality

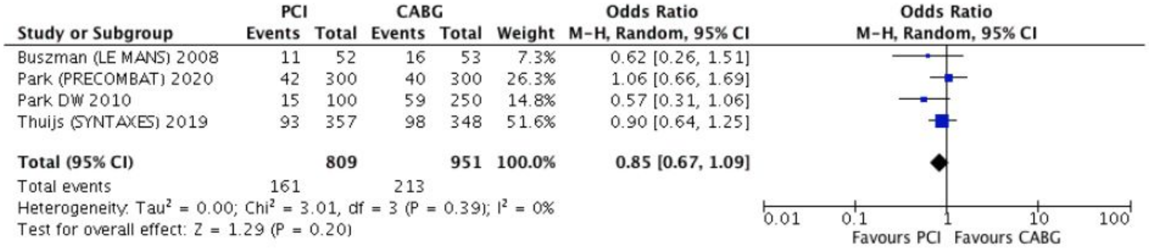

S. Figure 4: Forest Plots showing an individual and pooled RR for RCTs comparing PCI to CABG for LMCAD at 10-year (a. MACCE b. MI c. revascularization d. stroke e. death).

A. 30-days MACCE

Study or Subgroup	PCI Events	PCI Total	CABG Events	CABG Total	Weight	Risk Ratio M-H, Random, 95% CI
Buszman (LE MANS) 2008	1	52	7	53	1.6%	0.15 [0.02, 1.14]
Park (PRECOMBAT) 2011	4	300	9	300	5.1%	0.44 [0.14, 1.43]
Boudriot 2011	6	100	10	101	7.3%	0.61 [0.23, 1.60]
Makikallio (NOBLE) 2016	28	592	37	592	30.2%	0.76 [0.47, 1.22]
Stone (EXCEL) 2019	46	948	80	957	55.8%	0.58 [0.41, 0.82]
**Total (95% CI)**		**1992**		**2003**	**100.0%**	**0.61 [0.47, 0.79]**
Total events	85		143			

Heterogeneity: $Tau^2 = 0.00$; $Chi^2 = 3.02$, df = 4 (P = 0.56); $I^2 = 0\%$
Test for overall effect: Z = 3.71 (P = 0.0002)

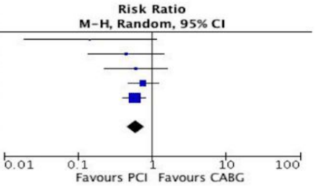

B. 30-days MI

Study or Subgroup	PCI Events	PCI Total	CABG Events	CABG Total	Weight	Risk Ratio M-H, Random, 95% CI
Buszman (LE MANS) 2008	1	52	2	53	2.0%	0.51 [0.05, 5.45]
Boudriot 2011	3	100	3	101	4.4%	1.01 [0.21, 4.89]
Makikallio (NOBLE) 2016	19	592	16	592	25.5%	1.19 [0.62, 2.29]
Stone (EXCEL) 2019	37	948	59	957	68.1%	0.63 [0.42, 0.95]
**Total (95% CI)**		**1692**		**1703**	**100.0%**	**0.76 [0.54, 1.05]**
Total events	60		80			

Heterogeneity: $Tau^2 = 0.00$; $Chi^2 = 2.81$, df = 3 (P = 0.42); $I^2 = 0\%$
Test for overall effect: Z = 1.66 (P = 0.10)

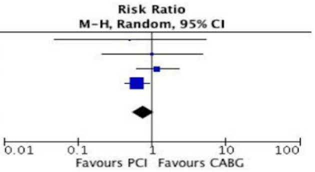

C. 30-days Revascularization

Study or Subgroup	PCI Events	PCI Total	CABG Events	CABG Total	Weight	Risk Ratio M-H, Random, 95% CI
Buszman (LE MANS) 2008	1	52	0	53	3.9%	3.06 [0.13, 73.36]
Boudriot 2011	1	100	2	101	6.9%	0.51 [0.05, 5.48]
Makikallio (NOBLE) 2016	7	592	10	592	42.5%	0.70 [0.27, 1.83]
Stone (EXCEL) 2019	7	948	13	957	46.8%	0.54 [0.22, 1.36]
**Total (95% CI)**		**1692**		**1703**	**100.0%**	**0.64 [0.34, 1.20]**
Total events	16		25			

Heterogeneity: $Tau^2 = 0.00$; $Chi^2 = 1.12$, df = 3 (P = 0.77); $I^2 = 0\%$
Test for overall effect: Z = 1.38 (P = 0.17)

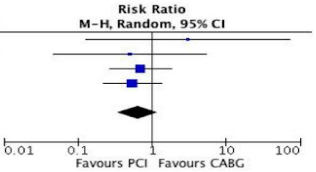

D. 30-days Stroke

Study or Subgroup	PCI Events	PCI Total	CABG Events	CABG Total	Weight	Risk Ratio M-H, Random, 95% CI
Buszman (LE MANS) 2008	0	52	2	53	8.6%	0.20 [0.01, 4.14]
Makikallio (NOBLE) 2016	0	592	4	592	9.2%	0.11 [0.01, 2.06]
Stone (EXCEL) 2019	6	948	12	957	82.2%	0.50 [0.19, 1.34]
**Total (95% CI)**		**1592**		**1602**	**100.0%**	**0.41 [0.17, 0.98]**
Total events	6		18			

Heterogeneity: $Tau^2 = 0.00$; $Chi^2 = 1.19$, df = 2 (P = 0.55); $I^2 = 0\%$
Test for overall effect: Z = 2.00 (P = 0.05)

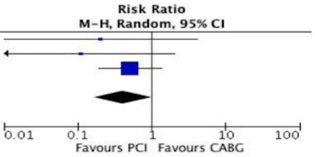

E. 30-days all-cause Mortality

Study or Subgroup	PCI Events	PCI Total	CABG Events	CABG Total	Weight	Risk Ratio M-H, Random, 95% CI
Buszman (LE MANS) 2008	0	52	2	53	5.1%	0.20 [0.01, 4.14]
Boudriot 2011	2	100	5	101	17.8%	0.40 [0.08, 2.03]
Makikallio (NOBLE) 2016	2	592	7	592	19.0%	0.29 [0.06, 1.37]
Stone (EXCEL) 2019	9	948	10	957	58.1%	0.91 [0.37, 2.23]
**Total (95% CI)**		**1692**		**1703**	**100.0%**	**0.58 [0.30, 1.16]**
Total events	13		24			

Heterogeneity: $Tau^2 = 0.00$; $Chi^2 = 2.42$, df = 3 (P = 0.49); $I^2 = 0\%$
Test for overall effect: Z = 1.54 (P = 0.12)

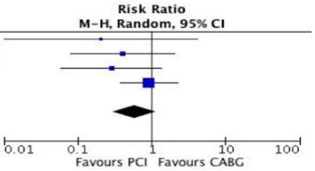

S. Figure 5: Forest Plots showing an individual and pooled RR for RCTs comparing PCI to CABG for LMCAD at 30-days (a. MACCE b. MI c. revascularization d. stroke e. death).

A. 1-year MAACE

Study or Subgroup	PCI Events	PCI Total	CABG Events	CABG Total	Weight	Risk Ratio M-H, Random, 95% CI
Boudriot 2011	19	100	14	101	4.7%	1.37 [0.73, 2.58]
Buszman (LE MANS) 2008	16	52	13	53	4.8%	1.25 [0.67, 2.34]
Park (PRECOMBAT) 2011	26	300	20	300	6.0%	1.30 [0.74, 2.28]
Makikallio (NOBLE) 2016	42	592	42	592	11.1%	1.00 [0.66, 1.51]
Serruys (SYNTAX) 2009	111	357	96	348	35.7%	1.13 [0.90, 1.42]
Stone (EXCEL) 2019	129	948	136	957	37.7%	0.96 [0.77, 1.20]
**Total (95% CI)**		**2349**		**2351**	**100.0%**	**1.07 [0.93, 1.23]**
Total events	343		321			

Heterogeneity: Tau² = 0.00; Chi² = 2.55, df = 5 (P = 0.77); I² = 0%
Test for overall effect: Z = 0.97 (P = 0.33)

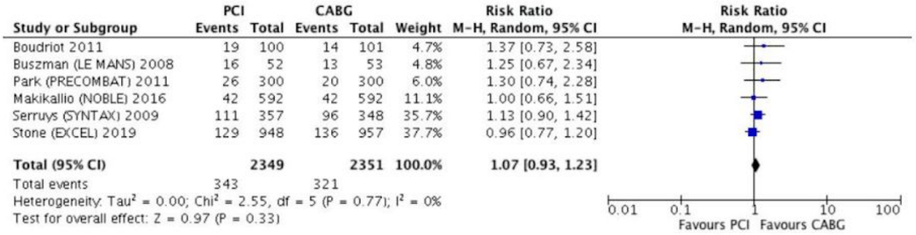

B. 1-year MI

Study or Subgroup	PCI Events	PCI Total	CABG Events	CABG Total	Weight	Risk Ratio M-H, Random, 95% CI
Buszman (LE MANS) 2008	1	52	3	53	2.9%	0.34 [0.04, 3.16]
Boudriot 2011	3	100	3	101	5.6%	1.01 [0.21, 4.89]
Park (PRECOMBAT) 2011	4	300	3	300	6.2%	1.33 [0.30, 5.91]
Makikallio (NOBLE) 2016	11	592	8	592	14.6%	1.38 [0.56, 3.39]
Serruys (SYNTAX) 2009	28	357	16	348	26.3%	1.71 [0.94, 3.10]
Stone (EXCEL) 2019	53	948	69	957	44.5%	0.78 [0.55, 1.10]
**Total (95% CI)**		**2349**		**2351**	**100.0%**	**1.06 [0.72, 1.57]**
Total events	100		102			

Heterogeneity: Tau² = 0.06; Chi² = 6.65, df = 5 (P = 0.25); I² = 25%
Test for overall effect: Z = 0.31 (P = 0.76)

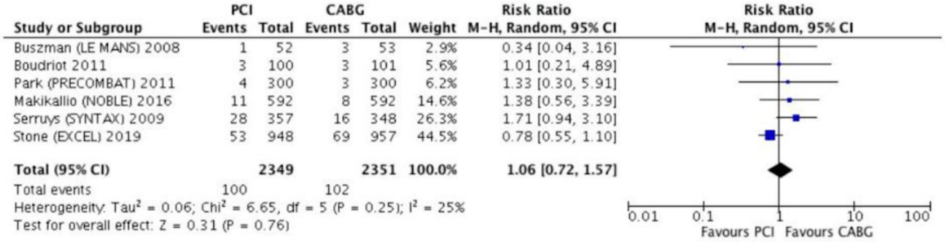

C. 1-year Revascularization

Study or Subgroup	PCI Events	PCI Total	CABG Events	CABG Total	Weight	Risk Ratio M-H, Random, 95% CI
Buszman (LE MANS) 2008	15	52	5	53	4.7%	3.06 [1.20, 7.80]
Boudriot 2011	14	100	6	101	5.0%	2.36 [0.94, 5.89]
Park (PRECOMBAT) 2011	18	300	10	300	7.3%	1.80 [0.84, 3.83]
Makikallio (NOBLE) 2016	32	592	24	592	15.6%	1.33 [0.80, 2.24]
Stone (EXCEL) 2019	65	948	41	957	28.8%	1.60 [1.09, 2.34]
Serruys (SYNTAX) 2009	83	357	46	348	38.6%	1.76 [1.27, 2.44]
**Total (95% CI)**		**2349**		**2351**	**100.0%**	**1.71 [1.39, 2.10]**
Total events	227		132			

Heterogeneity: Tau² = 0.00; Chi² = 3.00, df = 5 (P = 0.70); I² = 0%
Test for overall effect: Z = 5.15 (P < 0.00001)

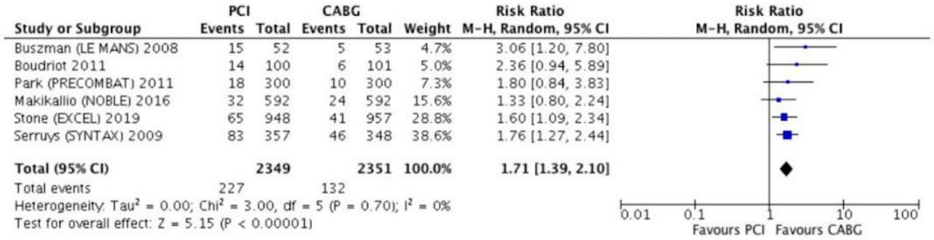

D. 1-year Stroke

Study or Subgroup	PCI Events	PCI Total	CABG Events	CABG Total	Weight	Risk Ratio M-H, Random, 95% CI
Boudriot 2011	0	0	0	0		Not estimable
Park (PRECOMBAT) 2011	0	300	2	300	5.9%	0.20 [0.01, 4.15]
Buszman (LE MANS) 2008	0	52	2	53	6.0%	0.20 [0.01, 4.14]
Makikallio (NOBLE) 2016	2	592	6	592	18.1%	0.33 [0.07, 1.64]
Serruys (SYNTAX) 2009	2	357	19	348	21.1%	0.10 [0.02, 0.44]
Stone (EXCEL) 2019	11	948	19	957	48.9%	0.58 [0.28, 1.22]
**Total (95% CI)**		**2249**		**2250**	**100.0%**	**0.32 [0.15, 0.69]**
Total events	15		48			

Heterogeneity: Tau² = 0.17; Chi² = 5.04, df = 4 (P = 0.28); I² = 21%
Test for overall effect: Z = 2.92 (P = 0.004)

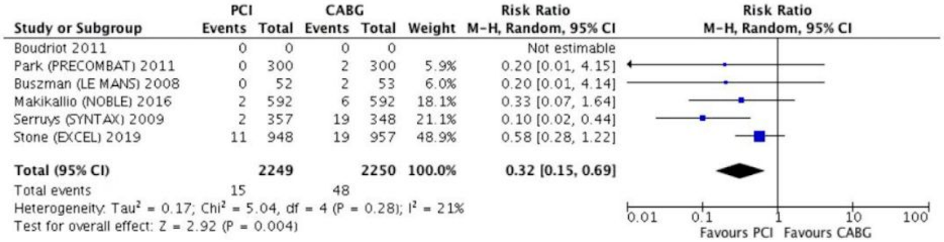

E. 1-year all-cause Mortality

Study or Subgroup	PCI Events	PCI Total	CABG Events	CABG Total	Weight	Risk Ratio M-H, Random, 95% CI
Buszman (LE MANS) 2008	1	52	4	53	1.5%	0.25 [0.03, 2.20]
Boudriot 2011	2	100	5	101	2.7%	0.40 [0.08, 2.03]
Park (PRECOMBAT) 2011	6	300	8	300	6.4%	0.75 [0.26, 2.14]
Makikallio (NOBLE) 2016	9	592	17	592	10.9%	0.53 [0.24, 1.18]
Stone (EXCEL) 2019	31	948	33	957	30.0%	0.95 [0.59, 1.54]
Serruys (SYNTAX) 2009	45	357	48	348	48.6%	0.91 [0.63, 1.33]
**Total (95% CI)**		**2349**		**2351**	**100.0%**	**0.83 [0.63, 1.07]**
Total events	94		115			

Heterogeneity: Tau² = 0.00; Chi² = 3.72, df = 5 (P = 0.59); I² = 0%
Test for overall effect: Z = 1.42 (P = 0.15)

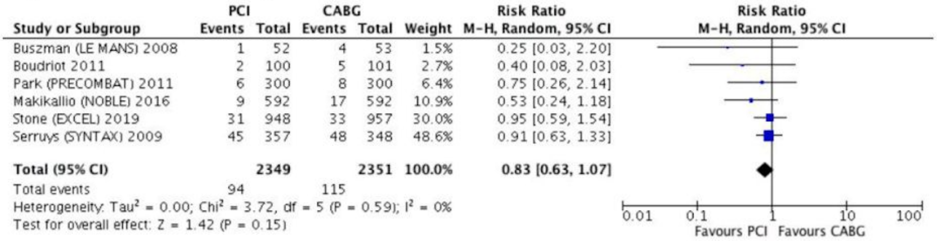

S. Figure 6: Forest Plots showing an individual and pooled RR for RCTs comparing PCI to CABG for LMCAD at 1-year (a. MACCE b. MI c. revascularization d. stroke e. death).

A. 5-year MAACE

Study or Subgroup	PCI Events	PCI Total	CABG Events	CABG Total	Weight	Risk Ratio M-H, Random, 95% CI
Ahn (PRECOMBAT) 2015	52	300	42	300	7.4%	1.24 [0.85, 1.80]
Holm (NOBLE) 2020	165	592	110	592	22.8%	1.50 [1.21, 1.86]
Morice (SYNTAX) 2014	130	357	103	348	22.9%	1.23 [1.00, 1.52]
Stone (EXCEL) 2019	290	948	228	957	46.9%	1.28 [1.11, 1.49]
**Total (95% CI)**		**2197**		**2197**	**100.0%**	**1.31 [1.19, 1.45]**
Total events	637		483			

Heterogeneity: $Tau^2 = 0.00$; $Chi^2 = 2.05$, df = 3 (P = 0.56); $I^2 = 0\%$
Test for overall effect: Z = 5.27 (P < 0.00001)

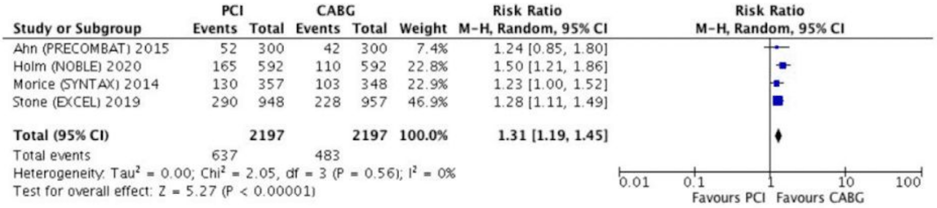

B. 5-year MI

Study or Subgroup	PCI Events	PCI Total	CABG Events	CABG Total	Weight	Risk Ratio M-H, Random, 95% CI
Ahn (PRECOMBAT) 2015	6	300	5	300	11.9%	1.20 [0.37, 3.89]
Holm (NOBLE) 2020	43	592	15	592	26.1%	2.87 [1.61, 5.10]
Morice (SYNTAX) 2014	28	357	16	348	25.4%	1.71 [0.94, 3.10]
Stone (EXCEL) 2019	95	948	84	957	36.6%	1.14 [0.86, 1.51]
**Total (95% CI)**		**2197**		**2197**	**100.0%**	**1.62 [1.00, 2.62]**
Total events	172		120			

Heterogeneity: $Tau^2 = 0.14$; $Chi^2 = 8.52$, df = 3 (P = 0.04); $I^2 = 65\%$
Test for overall effect: Z = 1.96 (P = 0.05)

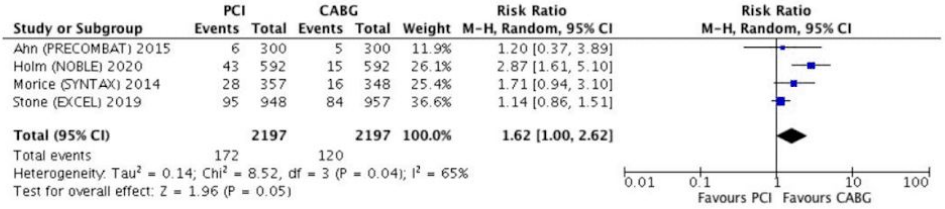

C. 5-year Revascularization

Study or Subgroup	PCI Events	PCI Total	CABG Events	CABG Total	Weight	Risk Ratio M-H, Random, 95% CI
Ahn (PRECOMBAT) 2015	38	300	21	300	9.3%	1.81 [1.09, 3.01]
Holm (NOBLE) 2020	97	592	58	592	25.8%	1.67 [1.23, 2.27]
Morice (SYNTAX) 2014	90	357	49	348	24.1%	1.79 [1.31, 2.45]
Stone (EXCEL) 2019	153	948	92	957	40.8%	1.68 [1.32, 2.14]
**Total (95% CI)**		**2197**		**2197**	**100.0%**	**1.72 [1.47, 2.00]**
Total events	378		220			

Heterogeneity: $Tau^2 = 0.00$; $Chi^2 = 0.17$, df = 3 (P = 0.98); $I^2 = 0\%$
Test for overall effect: Z = 6.83 (P < 0.00001)

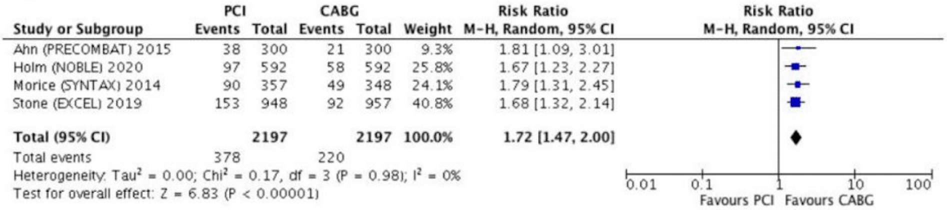

D. 5-year Stroke

Study or Subgroup	PCI Events	PCI Total	CABG Events	CABG Total	Weight	Risk Ratio M-H, Random, 95% CI
Ahn (PRECOMBAT) 2015	2	300	2	300	9.3%	1.00 [0.14, 7.05]
Holm (NOBLE) 2020	21	592	12	592	31.0%	1.75 [0.87, 3.52]
Morice (SYNTAX) 2014	5	357	14	348	22.6%	0.35 [0.13, 0.96]
Stone (EXCEL) 2019	26	948	33	957	37.0%	0.80 [0.48, 1.32]
**Total (95% CI)**		**2197**		**2197**	**100.0%**	**0.86 [0.44, 1.68]**
Total events	54		61			

Heterogeneity: $Tau^2 = 0.25$; $Chi^2 = 7.10$, df = 3 (P = 0.07); $I^2 = 58\%$
Test for overall effect: Z = 0.44 (P = 0.66)

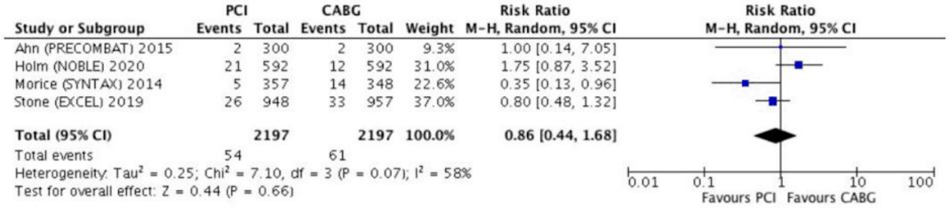

E. 5-year all-cause Mortality

Study or Subgroup	PCI Events	PCI Total	CABG Events	CABG Total	Weight	Risk Ratio M-H, Random, 95% CI
Ahn (PRECOMBAT) 2015	17	300	23	300	12.7%	0.74 [0.40, 1.36]
Holm (NOBLE) 2020	54	592	50	592	25.7%	1.08 [0.75, 1.56]
Morice (SYNTAX) 2014	45	357	48	348	24.8%	0.91 [0.63, 1.33]
Stone (EXCEL) 2019	119	948	89	957	36.8%	1.35 [1.04, 1.75]
**Total (95% CI)**		**2197**		**2197**	**100.0%**	**1.07 [0.84, 1.36]**
Total events	235		210			

Heterogeneity: $Tau^2 = 0.02$; $Chi^2 = 4.94$, df = 3 (P = 0.18); $I^2 = 39\%$
Test for overall effect: Z = 0.57 (P = 0.57)

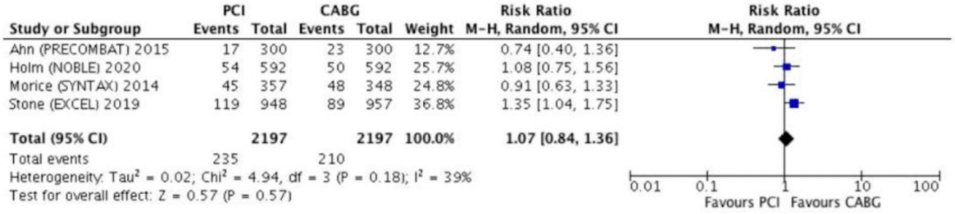

S. Figure 7: Forest Plots showing an individual and pooled RR for RCTs comparing PCI to CABG for LMCAD at 5-year (a. MACCE b. MI c. revascularization d. stroke e. death).

A. In Hospital MAACE

Study or Subgroup	PCI Events	PCI Total	CABG Events	CABG Total	Weight	Odds Ratio M-H, Random, 95% CI
HSU 2008	1	20	10	39	2.0%	0.15 [0.02, 1.29]
Yin 2015	1	106	7	121	2.0%	0.16 [0.02, 1.28]
Qin 2013	2	233	10	282	3.7%	0.24 [0.05, 1.09]
Ghenim 2009	4	105	6	106	5.1%	0.66 [0.18, 2.41]
Zhao 2011	5	56	36	116	8.1%	0.22 [0.08, 0.59]
Buchanan 2014	47	489	74	328	30.8%	0.36 [0.25, 0.54]
Chieffo 2012	148	1874	257	900	48.3%	0.21 [0.17, 0.27]
Total (95% CI)		2883		1892	100.0%	0.27 [0.20, 0.36]
Total events	208		400			

Heterogeneity: Tau² = 0.04; Chi² = 7.93, df = 6 (P = 0.24); I² = 24%
Test for overall effect: Z = 8.55 (P < 0.00001)

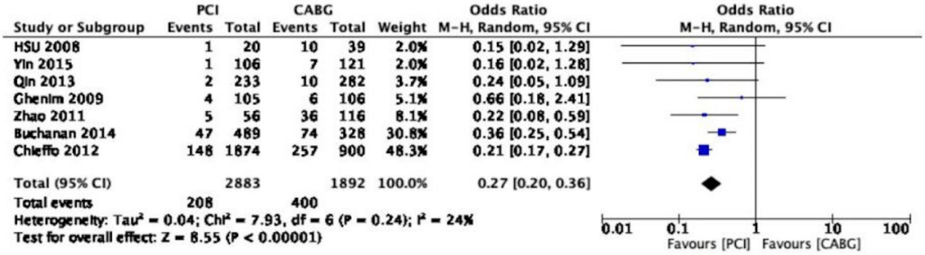

B. In Hospital MI

Study or Subgroup	PCI Events	PCI Total	CABG Events	CABG Total	Weight	Odds Ratio M-H, Random, 95% CI
HSU 2008	0	20	0	39		Not estimable
Qin 2013	0	233	1	282	5.6%	0.40 [0.02, 9.91]
Yin 2015	1	106	1	121	7.0%	1.14 [0.07, 18.50]
Cheng 2009	12	147	1	216	10.8%	19.11 [2.46, 148.65]
Zhao 2011	5	56	34	116	20.9%	0.24 [0.09, 0.64]
Buchanan 2014	29	489	60	328	27.0%	0.28 [0.18, 0.45]
Chieffo 2012	88	1874	213	900	28.7%	0.16 [0.12, 0.21]
Total (95% CI)		2925		2002	100.0%	0.41 [0.18, 0.95]
Total events	135		310			

Heterogeneity: Tau² = 0.63; Chi² = 27.30, df = 5 (P < 0.0001); I² = 82%
Test for overall effect: Z = 2.07 (P = 0.04)

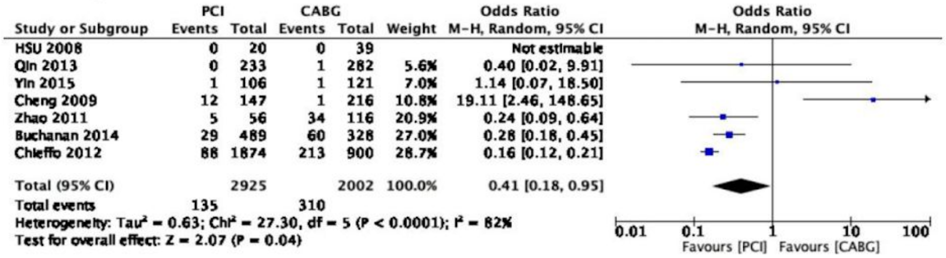

C. In Hospital Revascularization

Study or Subgroup	PCI Events	PCI Total	CABG Events	CABG Total	Weight	Odds Ratio M-H, Random, 95% CI
Ghenim 2009	0	105	0	106		Not estimable
HSU 2008	0	20	1	39	7.0%	0.63 [0.02, 16.07]
Yin 2015	0	106	1	121	7.1%	0.38 [0.02, 9.36]
Qin 2013	0	233	1	282	7.1%	0.40 [0.02, 9.91]
Buchanan 2014	4	489	0	328	8.6%	6.09 [0.33, 113.49]
Cheng 2009	2	147	3	216	22.6%	0.98 [0.16, 5.93]
Chieffo 2012	15	1874	3	900	47.6%	2.41 [0.70, 8.35]
Total (95% CI)		2974		1992	100.0%	1.49 [0.63, 3.52]
Total events	21		9			

Heterogeneity: Tau² = 0.00; Chi² = 3.33, df = 5 (P = 0.65); I² = 0%
Test for overall effect: Z = 0.92 (P = 0.36)

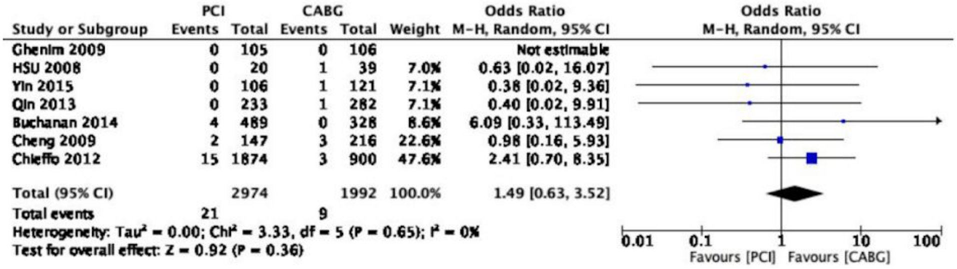

D. In Hospital Stroke

Study or Subgroup	PCI Events	PCI Total	CABG Events	CABG Total	Weight	Odds Ratio M-H, Random, 95% CI
HSU 2008	0	20	1	39	7.5%	0.63 [0.02, 16.07]
Yin 2015	0	106	1	121	7.7%	0.38 [0.02, 9.36]
Cheng 2009	0	147	14	216	9.9%	0.05 [0.00, 0.80]
Qin 2013	1	233	2	282	13.6%	0.60 [0.05, 6.70]
Chieffo 2012	4	1874	12	900	61.3%	0.16 [0.05, 0.49]
Total (95% CI)		2380		1558	100.0%	0.20 [0.08, 0.49]
Total events	5		30			

Heterogeneity: Tau² = 0.00; Chi² = 2.75, df = 4 (P = 0.60); I² = 0%
Test for overall effect: Z = 3.55 (P = 0.0004)

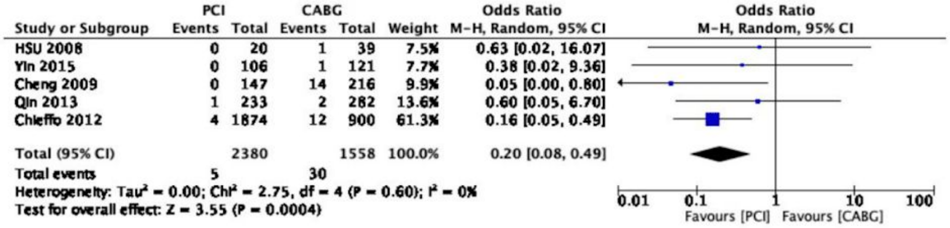

E. In Hospital all-cause Mortality

Study or Subgroup	PCI Events	PCI Total	CABG Events	CABG Total	Weight	Odds Ratio M-H, Random, 95% CI
Zhao 2011	0	56	4	116	5.6%	0.22 [0.01, 4.18]
Yin 2015	0	106	4	121	5.6%	0.12 [0.01, 2.30]
HSU 2008	1	20	8	39	8.9%	0.20 [0.02, 1.76]
Qin 2013	2	233	15	282	14.1%	0.15 [0.03, 0.68]
Cheng 2009	3	147	22	216	16.9%	0.18 [0.05, 0.63]
Buchanan 2014	20	489	9	328	22.4%	1.51 [0.68, 3.36]
Chieffo 2012	41	1874	29	900	26.5%	0.67 [0.41, 1.09]
Total (95% CI)		2925		2002	100.0%	0.40 [0.19, 0.87]
Total events	67		91			

Heterogeneity: Tau² = 0.52; Chi² = 15.08, df = 6 (P = 0.02); I² = 60%
Test for overall effect: Z = 2.30 (P = 0.02)

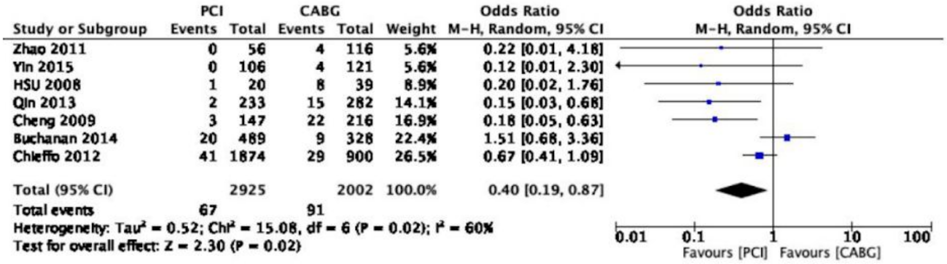

S. Figure 8: In-hospital pooled estimates of PCI vs. CABG events for LMCAD across observational cohort studies.

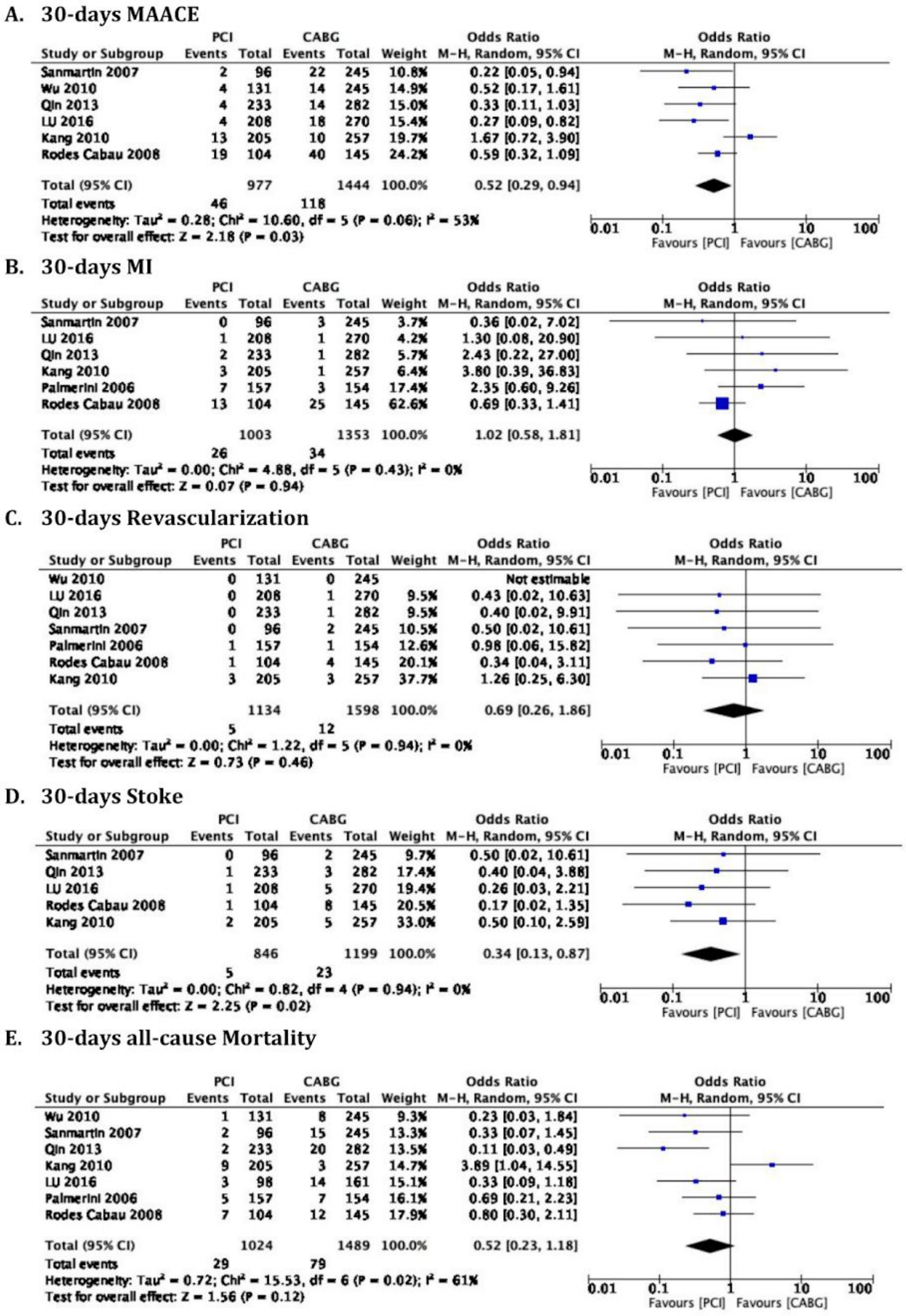

S. Figure 9: 30-days pooled estimates of PCI vs. CABG events for LMCAD across observational cohort studies.

A. 6-month MAACE

Study or Subgroup	PCI Events	PCI Total	CABG Events	CABG Total	Weight	Odds Ratio M-H, Random, 95% CI
Kang 2010	21	205	23	257	100.0%	1.16 [0.62, 2.16]
Total (95% CI)		205		257	100.0%	1.16 [0.62, 2.16]
Total events	21		23			

Heterogeneity: Not applicable
Test for overall effect: Z = 0.47 (P = 0.64)

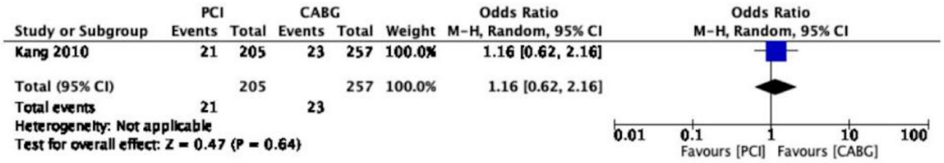

B. 6-month MI

Study or Subgroup	PCI Events	PCI Total	CABG Events	CABG Total	Weight	Odds Ratio M-H, Random, 95% CI
Kang 2010	3	205	1	257	23.4%	3.80 [0.39, 36.83]
Montalescot 2009	14	293	3	417	76.6%	6.92 [1.97, 24.32]
Total (95% CI)		498		674	100.0%	6.02 [2.00, 18.06]
Total events	17		4			

Heterogeneity: $Tau^2 = 0.00$; $Chi^2 = 0.21$, df = 1 (P = 0.65); $I^2 = 0\%$
Test for overall effect: Z = 3.20 (P = 0.001)

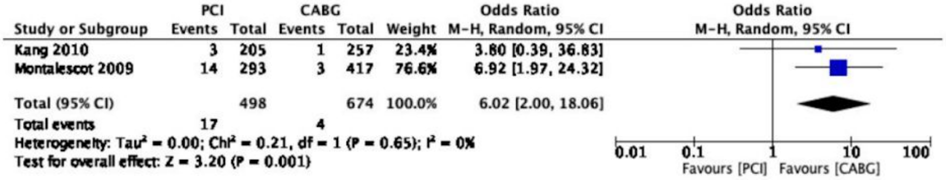

C. 6-month Revascularization

Study or Subgroup	PCI Events	PCI Total	CABG Events	CABG Total	Weight	Odds Ratio M-H, Random, 95% CI
Kang 2010	9	205	6	257	100.0%	1.92 [0.67, 5.49]
Total (95% CI)		205		257	100.0%	1.92 [0.67, 5.49]
Total events	9		6			

Heterogeneity: Not applicable
Test for overall effect: Z = 1.22 (P = 0.22)

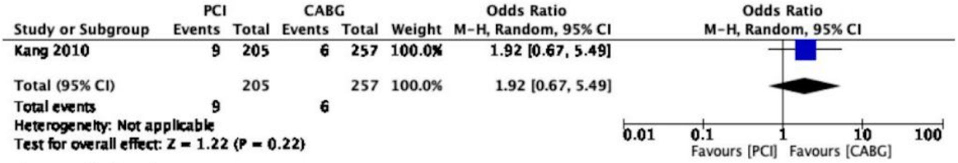

D. 6-month Stroke

Study or Subgroup	PCI Events	PCI Total	CABG Events	CABG Total	Weight	Odds Ratio M-H, Random, 95% CI
Kang 2010	2	205	8	257	46.0%	0.31 [0.06, 1.46]
Montalescot 2009	3	295	5	421	54.0%	0.85 [0.20, 3.60]
Total (95% CI)		500		678	100.0%	0.53 [0.19, 1.54]
Total events	5		13			

Heterogeneity: $Tau^2 = 0.00$; $Chi^2 = 0.90$, df = 1 (P = 0.34); $I^2 = 0\%$
Test for overall effect: Z = 1.16 (P = 0.24)

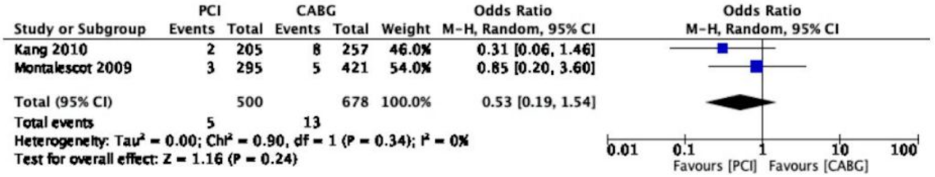

E. 6-month all-cause Mortality

Study or Subgroup	PCI Events	PCI Total	CABG Events	CABG Total	Weight	Odds Ratio M-H, Random, 95% CI
Montalescot 2009	17	312	7	437	49.0%	3.54 [1.45, 8.64]
Kang 2010	11	205	13	257	51.0%	1.06 [0.47, 2.43]
Total (95% CI)		517		694	100.0%	1.92 [0.59, 6.23]
Total events	28		20			

Heterogeneity: $Tau^2 = 0.53$; $Chi^2 = 3.76$, df = 1 (P = 0.05); $I^2 = 73\%$
Test for overall effect: Z = 1.08 (P = 0.28)

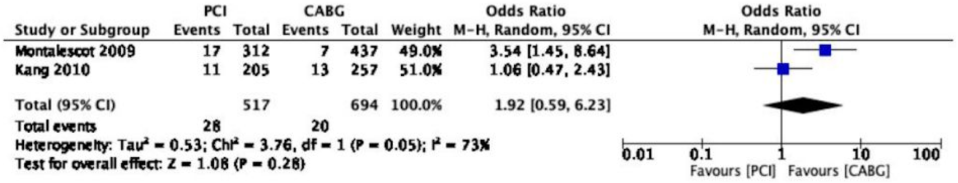

S. Figure 10: 6-months pooled estimates of PCI vs. CABG events for LMCAD across observational cohort studies.

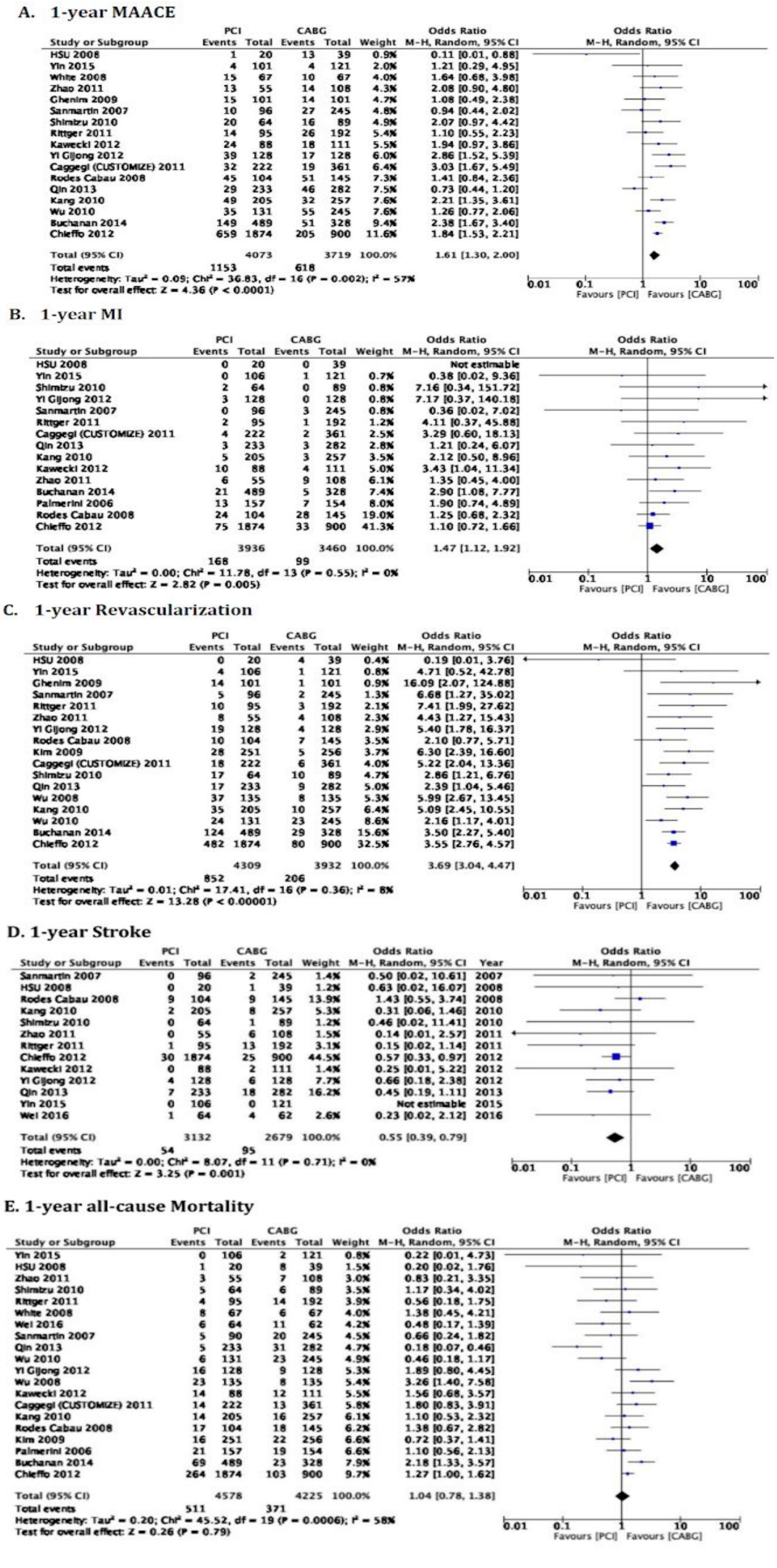
A. 1-year MAACE
PCI CABG Odds Ratio Odds Ratio
Study or Subgroup Events Total Events Total Weight M-H, Random, 95% CI M-H, Random, 95% CI
HSU 2008 1 20 13 39 0.9% 0.11 [0.01, 0.88]
Yin 2015 4 101 4 121 2.0% 1.21 [0.29, 4.95]
White 2008 15 67 10 67 4.0% 1.64 [0.68, 3.98]
Zhao 2011 13 55 14 108 4.3% 2.08 [0.90, 4.80]
Ghenim 2009 15 101 14 101 4.7% 1.08 [0.49, 2.38]
Sanmartin 2007 10 96 27 245 4.8% 0.94 [0.44, 2.02]
Shimizu 2010 20 64 16 89 4.9% 2.07 [0.97, 4.42]
Rittger 2011 14 95 26 192 5.4% 1.10 [0.55, 2.23]
Kawecki 2012 24 88 18 111 5.5% 1.94 [0.97, 3.86]
Yi Gijong 2012 39 128 17 128 6.0% 2.86 [1.52, 5.39]
Caggegi (CUSTOMIZE) 2011 32 222 19 361 6.4% 3.03 [1.67, 5.49]
Rodes Cabau 2008 45 104 51 145 7.3% 1.41 [0.84, 2.36]
Qin 2013 29 233 46 282 7.5% 0.73 [0.44, 1.20]
Kang 2010 49 205 32 257 7.6% 2.21 [1.35, 3.61]
Wu 2010 35 131 55 245 7.6% 1.26 [0.77, 2.06]
Buchanan 2014 149 489 51 328 9.4% 2.38 [1.67, 3.40]
Chieffo 2012 659 1874 205 900 11.6% 1.84 [1.53, 2.21]
Total (95% CI) 4073 3719 100.0% 1.61 [1.30, 2.00]
Total events 1153 618
Heterogeneity: Tau² = 0.09; Chi² = 36.83, df = 16 (P = 0.002); I² = 57%
Test for overall effect: Z = 4.36 (P < 0.0001)
0.01 0.1 1 10 100
Favours [PCI] Favours [CABG]
B. 1-year MI
PCI CABG Odds Ratio Odds Ratio
Study or Subgroup Events Total Events Total Weight M-H, Random, 95% CI M-H, Random, 95% CI
HSU 2008 0 20 0 39 Not estimable
Yin 2015 0 106 1 121 0.7% 0.38 [0.02, 9.36]
Shimizu 2010 2 64 0 89 0.8% 7.16 [0.34, 151.72]
Yi Gijong 2012 3 128 0 128 0.8% 7.17 [0.37, 140.18]
Sanmartin 2007 0 96 3 245 0.8% 0.36 [0.02, 7.02]
Rittger 2011 2 95 1 192 1.2% 4.11 [0.37, 45.88]
Caggegi (CUSTOMIZE) 2011 4 222 2 361 2.5% 3.29 [0.60, 18.13]
Qin 2013 3 233 3 282 2.8% 1.21 [0.24, 6.07]
Kang 2010 5 205 3 257 3.5% 2.12 [0.50, 8.96]
Kawecki 2012 10 88 4 111 5.0% 3.43 [1.04, 11.34]
Zhao 2011 6 55 9 108 6.1% 1.35 [0.45, 4.00]
Buchanan 2014 21 489 5 328 7.4% 2.90 [1.08, 7.77]
Palmerini 2006 13 157 7 154 8.0% 1.90 [0.74, 4.89]
Rodes Cabau 2008 24 104 28 145 19.0% 1.25 [0.68, 2.32]
Chieffo 2012 75 1874 33 900 41.3% 1.10 [0.72, 1.66]
Total (95% CI) 3936 3460 100.0% 1.47 [1.12, 1.92]
Total events 168 99
Heterogeneity: Tau² = 0.00; Chi² = 11.78, df = 13 (P = 0.55); I² = 0%
Test for overall effect: Z = 2.82 (P = 0.005)
0.01 0.1 1 10 100
Favours [PCI] Favours [CABG]
C. 1-year Revascularization
PCI CABG Odds Ratio Odds Ratio
Study or Subgroup Events Total Events Total Weight M-H, Random, 95% CI M-H, Random, 95% CI
HSU 2008 0 20 4 39 0.4% 0.19 [0.01, 3.76]
Yin 2015 4 106 1 121 0.8% 4.71 [0.52, 42.78]
Ghenim 2009 14 101 1 101 0.9% 16.09 [2.07, 124.88]
Sanmartin 2007 5 96 2 245 1.3% 6.68 [1.27, 35.02]
Rittger 2011 10 95 3 192 2.1% 7.41 [1.99, 27.62]
Zhao 2011 8 55 4 108 2.3% 4.43 [1.27, 15.43]
Yi Gijong 2012 19 128 4 128 2.9% 5.40 [1.78, 16.37]
Rodes Cabau 2008 10 104 7 145 3.5% 2.10 [0.77, 5.71]
Kim 2009 28 251 5 256 3.7% 6.30 [2.39, 16.60]
Caggegi (CUSTOMIZE) 2011 18 222 6 361 4.0% 5.22 [2.04, 13.36]
Shimizu 2010 17 64 10 89 4.7% 2.86 [1.21, 6.76]
Qin 2013 17 233 9 282 5.0% 2.39 [1.04, 5.46]
Wu 2008 37 135 8 135 5.3% 5.99 [2.67, 13.45]
Kang 2010 35 205 10 257 6.4% 5.09 [2.45, 10.55]
Wu 2010 24 131 23 245 8.6% 2.16 [1.17, 4.01]
Buchanan 2014 124 489 29 328 15.6% 3.50 [2.27, 5.40]
Chieffo 2012 482 1874 80 900 32.5% 3.55 [2.76, 4.57]
Total (95% CI) 4309 3932 100.0% 3.69 [3.04, 4.47]
Total events 852 206
Heterogeneity: Tau² = 0.01; Chi² = 17.41, df = 16 (P = 0.36); I² = 8%
Test for overall effect: Z = 13.28 (P < 0.00001)
0.01 0.1 1 10 100
Favours [PCI] Favours [CABG]
D. 1-year Stroke
PCI CABG Odds Ratio Odds Ratio
Study or Subgroup Events Total Events Total Weight M-H, Random, 95% CI Year M-H, Random, 95% CI
Sanmartin 2007 0 96 2 245 1.4% 0.50 [0.02, 10.61] 2007
HSU 2008 0 20 1 39 1.2% 0.63 [0.02, 16.07] 2008
Rodes Cabau 2008 9 104 9 145 13.9% 1.43 [0.55, 3.74] 2008
Kang 2010 2 205 8 257 5.3% 0.31 [0.06, 1.46] 2010
Shimizu 2010 0 64 1 89 1.2% 0.46 [0.02, 11.41] 2010
Zhao 2011 0 55 6 108 1.5% 0.14 [0.01, 2.57] 2011
Rittger 2011 1 95 13 192 3.1% 0.15 [0.02, 1.14] 2011
Chieffo 2012 30 1874 25 900 44.5% 0.57 [0.33, 0.97] 2012
Kawecki 2012 0 88 2 111 1.4% 0.25 [0.01, 5.22] 2012
Yi Gijong 2012 4 128 6 128 7.7% 0.66 [0.18, 2.38] 2012
Qin 2013 7 233 18 282 16.2% 0.45 [0.19, 1.11] 2013
Yin 2015 0 106 0 121 Not estimable 2015
Wei 2016 1 64 4 62 2.6% 0.23 [0.02, 2.12] 2016
Total (95% CI) 3132 2679 100.0% 0.55 [0.39, 0.79]
Total events 54 95
Heterogeneity: Tau² = 0.00; Chi² = 8.07, df = 11 (P = 0.71); I² = 0%
Test for overall effect: Z = 3.25 (P = 0.001)
0.01 0.1 1 10 100
Favours [PCI] Favours [CABG]
E. 1-year all-cause Mortality
PCI CABG Odds Ratio Odds Ratio
Study or Subgroup Events Total Events Total Weight M-H, Random, 95% CI M-H, Random, 95% CI
Yin 2015 0 106 2 121 0.8% 0.22 [0.01, 4.73]
HSU 2008 1 20 8 39 1.5% 0.20 [0.02, 1.76]
Zhao 2011 3 55 7 108 3.0% 0.83 [0.21, 3.35]
Shimizu 2010 5 64 6 89 3.5% 1.17 [0.34, 4.02]
Rittger 2011 4 95 14 192 3.9% 0.56 [0.18, 1.75]
White 2008 8 67 6 67 4.0% 1.38 [0.45, 4.21]
Wei 2016 6 64 11 62 4.2% 0.48 [0.17, 1.39]
Sanmartin 2007 5 90 20 245 4.5% 0.66 [0.24, 1.82]
Qin 2013 5 233 31 282 4.7% 0.18 [0.07, 0.46]
Wu 2010 6 131 23 245 4.9% 0.46 [0.18, 1.17]
Yi Gijong 2012 16 128 9 128 5.3% 1.89 [0.80, 4.45]
Wu 2008 23 135 8 135 5.4% 3.26 [1.40, 7.58]
Kawecki 2012 14 88 12 111 5.5% 1.56 [0.68, 3.57]
Caggegi (CUSTOMIZE) 2011 14 222 13 361 5.8% 1.80 [0.83, 3.91]
Kang 2010 14 205 16 257 6.1% 1.10 [0.53, 2.32]
Rodes Cabau 2008 17 104 18 145 6.2% 1.38 [0.67, 2.82]
Kim 2009 16 251 22 256 6.6% 0.72 [0.37, 1.41]
Palmerini 2006 21 157 19 154 6.6% 1.10 [0.56, 2.13]
Buchanan 2014 69 489 23 328 7.9% 2.18 [1.33, 3.57]
Chieffo 2012 264 1874 103 900 9.7% 1.27 [1.00, 1.62]
Total (95% CI) 4578 4225 100.0% 1.04 [0.78, 1.38]
Total events 511 371
Heterogeneity: Tau² = 0.20; Chi² = 45.52, df = 19 (P = 0.0006); I² = 58%
Test for overall effect: Z = 0.26 (P = 0.79)
0.01 0.1 1 10 100
Favours [PCI] Favours [CABG]

S. Figure 11: 1-year pooled estimates of PCI vs. CABG events for LMCAD across observational cohort studies.

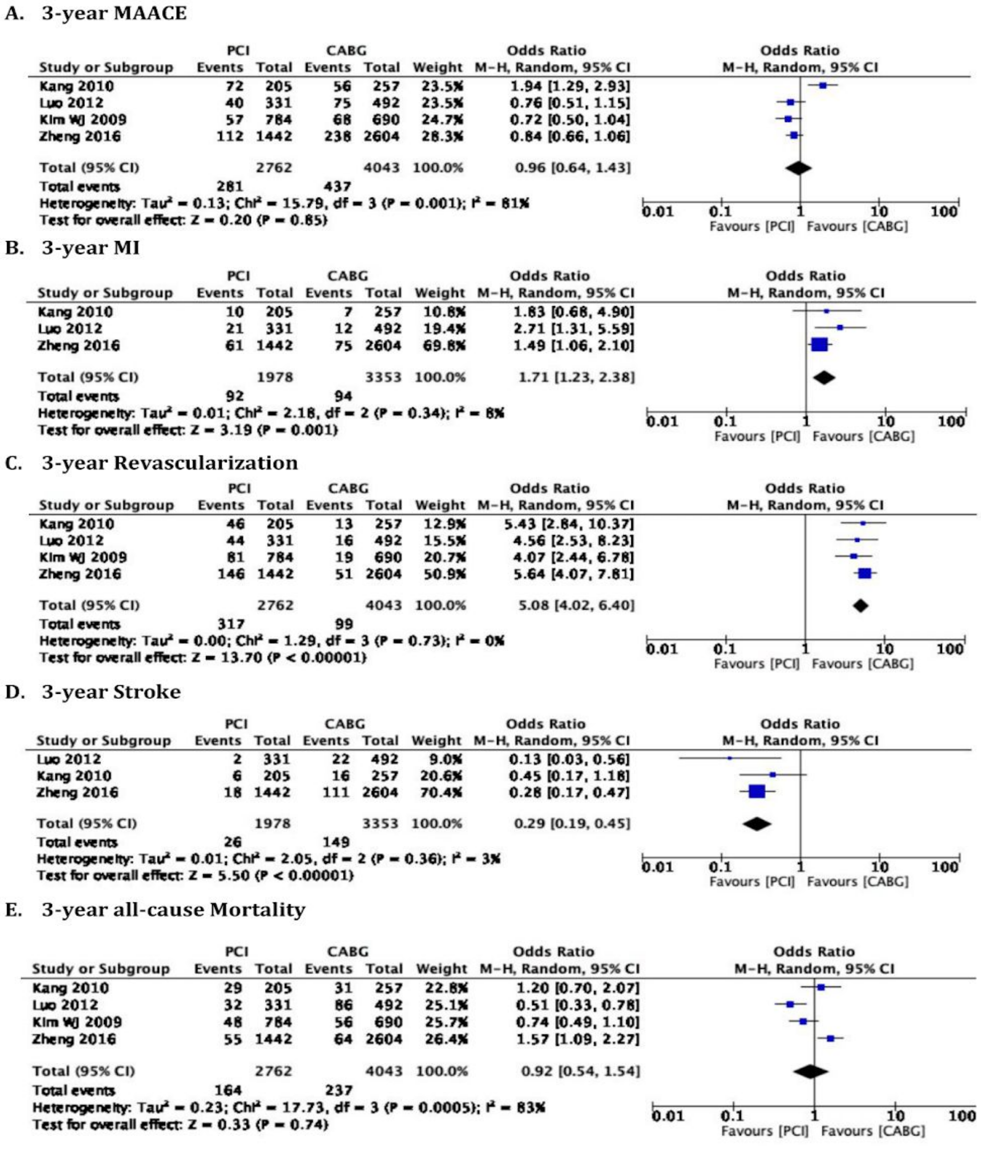

S. Figure 12: 3-year pooled estimates of PCI vs. CABG events for LMCAD across observational cohort studies.

A. 5-year MAACE

Study or Subgroup	PCI Events	PCI Total	CABG Events	CABG Total	Weight	Odds Ratio M-H, Random, 95% CI
Brener 2008	7	97	12	190	9.0%	1.15 [0.44, 3.03]
Park (MAIN COMPARE) 2011	149	784	7	690	10.0%	22.89 [10.65, 49.23]
Park DW 2010	17	176	41	219	10.7%	0.46 [0.25, 0.85]
Chang 2012	63	556	51	309	11.5%	0.65 [0.43, 0.96]
Shiomi (CREDO KYOTO) 2015	121	364	47	640	11.6%	6.28 [4.35, 9.08]
LU 2016	91	208	114	270	11.6%	1.06 [0.74, 1.53]
Delta Registry 2014	149	489	51	328	11.7%	2.38 [1.67, 3.40]
Yu 2016	232	465	178	457	11.9%	1.56 [1.20, 2.03]
Cavalcante 2016	186	657	149	648	11.9%	1.32 [1.03, 1.70]
Total (95% CI)		3796		3751	100.0%	1.82 [1.04, 3.21]
Total events	1015		650			

Heterogeneity: Tau² = 0.68; Chi² = 151.01, df = 8 (P < 0.00001); I² = 95%
Test for overall effect: Z = 2.08 (P = 0.04)

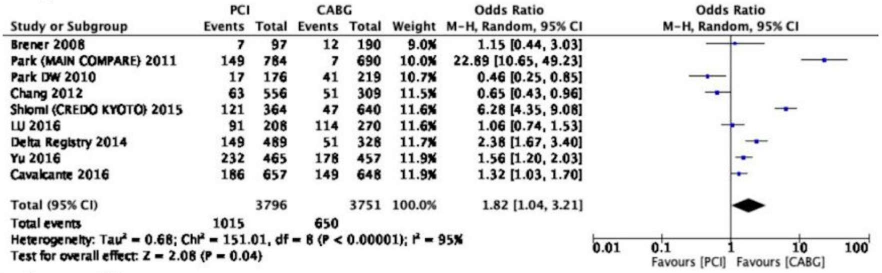

B. 5-year MI

Study or Subgroup	PCI Events	PCI Total	CABG Events	CABG Total	Weight	Odds Ratio M-H, Random, 95% CI
Chang 2012	5	556	3	309	2.8%	0.93 [0.22, 3.90]
Delta Registry 2014	21	489	5	328	6.1%	2.90 [1.08, 7.77]
LU 2016	17	208	18	270	12.4%	1.25 [0.63, 2.48]
Shiomi (CREDO KYOTO) 2015	25	364	19	640	15.8%	2.41 [1.31, 4.44]
Park DW 2010	27	176	21	219	15.9%	1.71 [0.93, 3.14]
Cavalcante 2016	34	657	21	648	19.1%	1.63 [0.94, 2.84]
Yu 2016	53	465	32	457	27.9%	1.71 [1.08, 2.70]
Total (95% CI)		2915		2871	100.0%	1.74 [1.37, 2.22]
Total events	182		119			

Heterogeneity: Tau² = 0.00; Chi² = 3.83, df = 6 (P = 0.70); I² = 0%
Test for overall effect: Z = 4.49 (P < 0.00001)

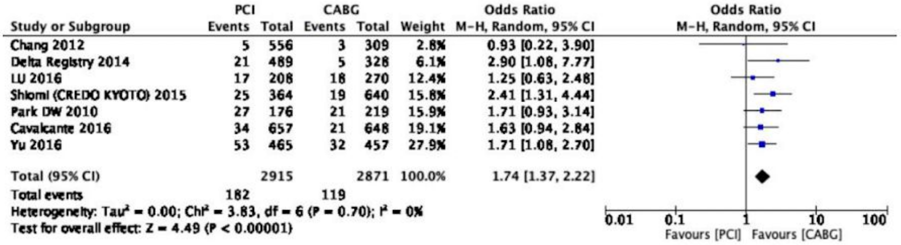

C. 5-year Revascularization

Study or Subgroup	PCI Events	PCI Total	CABG Events	CABG Total	Weight	Odds Ratio M-H, Random, 95% CI
Park DW 2010	32	176	10	219	8.7%	4.64 [2.21, 9.75]
Chang 2012	88	556	9	309	9.2%	6.27 [3.11, 12.64]
Delta Registry 2014	74	489	17	328	11.4%	3.26 [1.89, 5.64]
LU 2016	47	208	31	270	12.2%	2.25 [1.37, 3.69]
Park (MAIN COMPARE) 2011	123	784	22	690	12.7%	5.65 [3.54, 9.01]
Cavalcante 2016	128	657	70	648	15.2%	2.00 [1.46, 2.74]
Shiomi (CREDO KYOTO) 2015	148	364	84	640	15.2%	4.54 [3.32, 6.19]
Yu 2016	171	465	80	457	15.3%	2.74 [2.02, 3.72]
Total (95% CI)		3699		3561	100.0%	3.48 [2.57, 4.71]
Total events	811		323			

Heterogeneity: Tau² = 0.13; Chi² = 26.72, df = 7 (P = 0.0004); I² = 74%
Test for overall effect: Z = 8.06 (P < 0.00001)

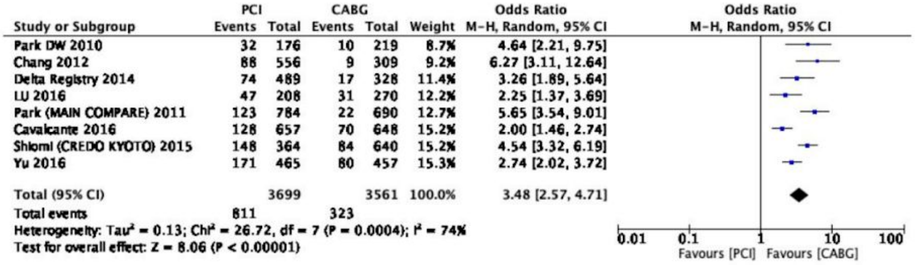

D. 5-year Stroke

Study or Subgroup	PCI Events	PCI Total	CABG Events	CABG Total	Weight	Odds Ratio M-H, Random, 95% CI
Chang 2012	1	556	4	309	4.9%	0.14 [0.02, 1.23]
Park DW 2010	3	176	12	219	10.4%	0.30 [0.08, 1.08]
LU 2016	4	208	14	270	11.9%	0.36 [0.12, 1.11]
Cavalcante 2016	7	657	16	648	14.8%	0.43 [0.17, 1.04]
Park (MAIN COMPARE) 2011	12	784	13	690	16.2%	0.81 [0.37, 1.79]
Shiomi (CREDO KYOTO) 2015	27	364	42	640	20.5%	1.14 [0.69, 1.88]
Yu 2016	30	465	80	457	21.4%	0.33 [0.21, 0.51]
Total (95% CI)		3210		3233	100.0%	0.49 [0.28, 0.84]
Total events	84		181			

Heterogeneity: Tau² = 0.30; Chi² = 17.75, df = 6 (P = 0.007); I² = 66%
Test for overall effect: Z = 2.61 (P = 0.009)

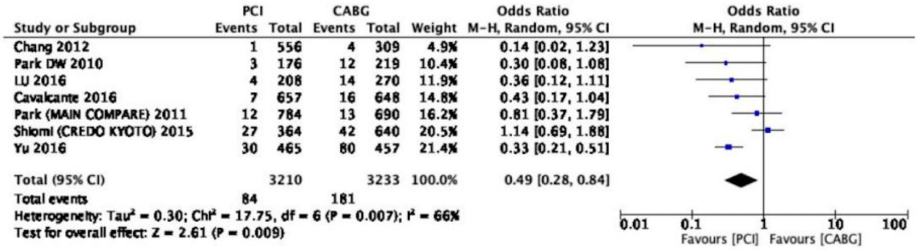

E. 5-year all-cause Mortality

Study or Subgroup	PCI Events	PCI Total	CABG Events	CABG Total	Weight	Odds Ratio M-H, Random, 95% CI
Park DW 2010	10	176	24	219	7.7%	0.49 [0.23, 1.05]
Delta Registry 2014	69	489	23	328	11.3%	2.18 [1.33, 3.57]
Chang 2012	57	556	45	309	12.5%	0.67 [0.44, 1.02]
LU 2016	57	208	85	270	12.8%	0.82 [0.55, 1.22]
Cavalcante 2016	62	657	72	648	13.4%	0.83 [0.58, 1.19]
Yu 2016	73	465	89	457	13.7%	0.77 [0.55, 1.08]
Shiomi (CREDO KYOTO) 2015	88	364	109	640	14.1%	1.55 [1.13, 2.13]
Park (MAIN COMPARE) 2011	95	784	106	690	14.4%	0.76 [0.56, 1.02]
Total (95% CI)		3699		3561	100.0%	0.92 [0.69, 1.23]
Total events	511		553			

Heterogeneity: Tau² = 0.13; Chi² = 30.23, df = 7 (P < 0.0001); I² = 77%
Test for overall effect: Z = 0.54 (P = 0.59)

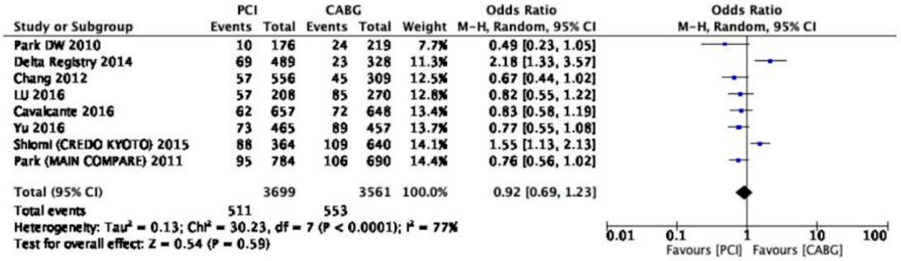

S. Figure 13: 5-year pooled estimates of PCI vs. CABG events for LMCAD across observational cohort studies.

E. SUPPLEMENTAL FUNNEL PLOTS FOR ALL STUDIES:

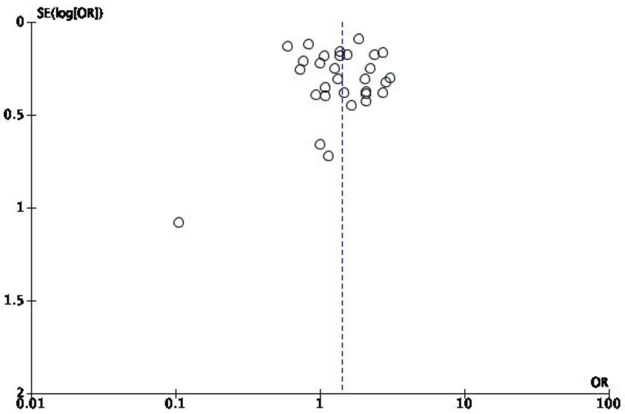

S. Figure 14: Funnel plot showing minimal publication bias across studies comparing the pooled estimate of MACCE at 1-year follow up.

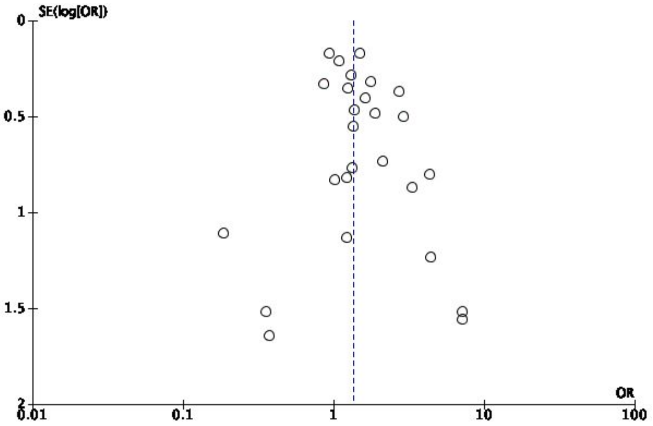

S. Figure 15: Funnel plot showing minimal publication bias across studies comparing the pooled estimate of MI at 1-year follow up.

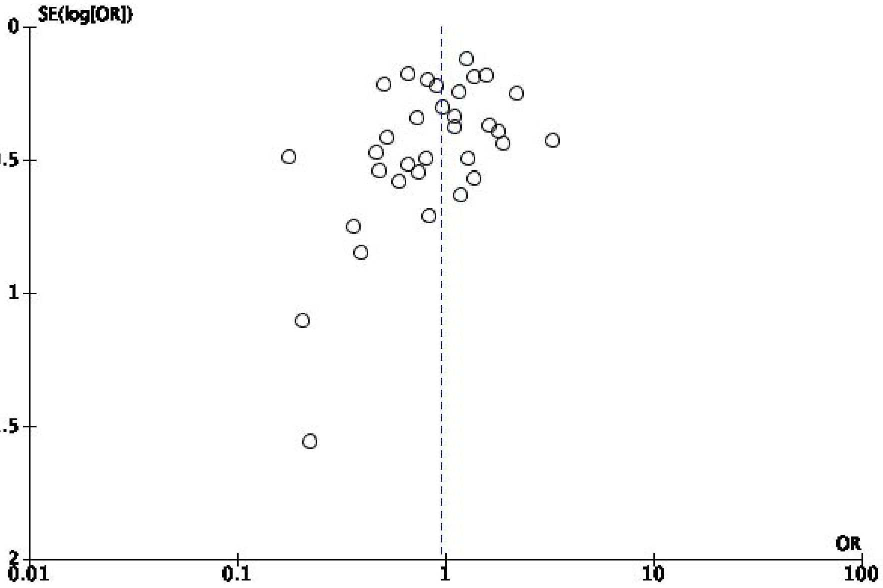

S. Figure 16: Funnel plot showing minimal publication bias across studies comparing the pooled estimate of mortality at 1-year follow up.

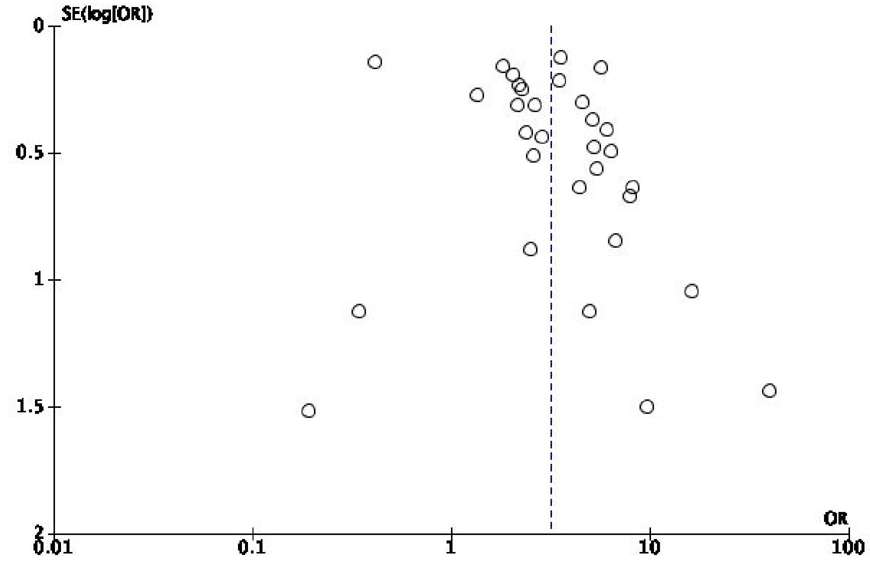

S. Figure 17: Funnel plot showing minimal to moderate publication bias across studies comparing the pooled estimate of revascularization at 1-year follow up.

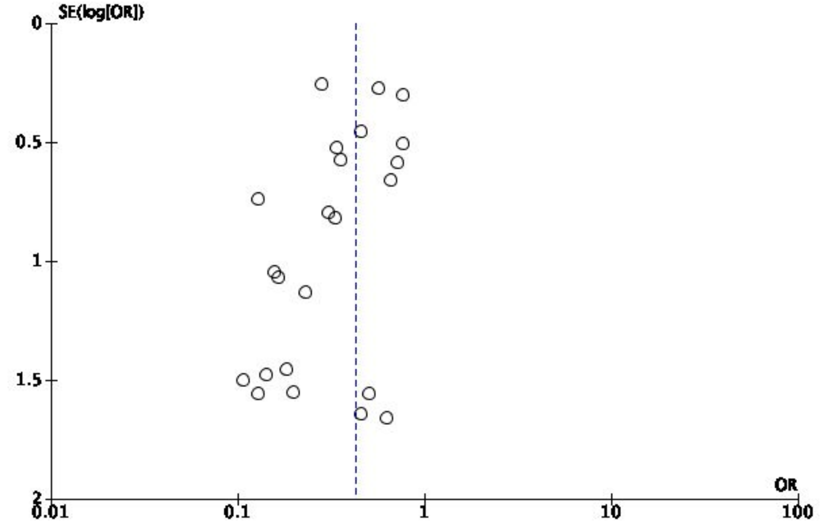

S. Figure 18: Funnel plot showing minimal to moderate publication bias across studies comparing the pooled estimate of a stroke at 1-year follow up.

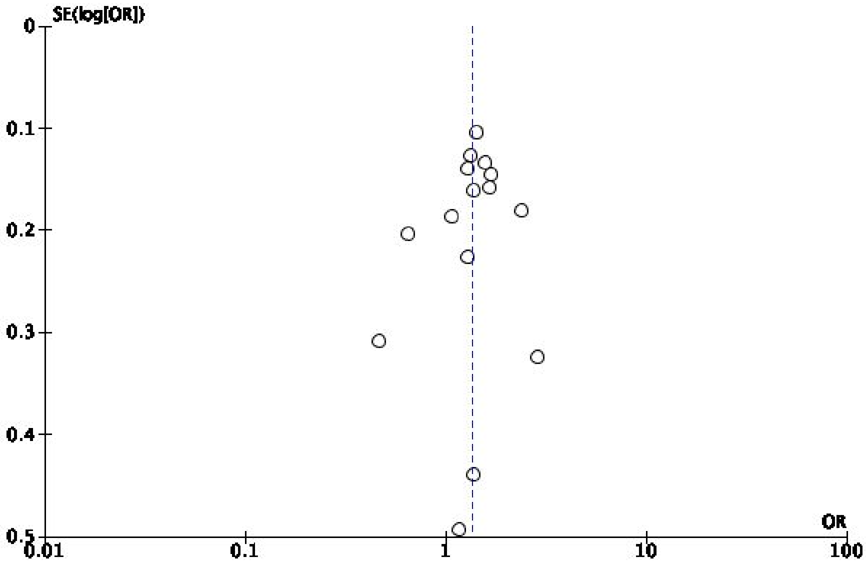

S. Figure 19: Funnel plot showing minimal publication bias across studies comparing the pooled estimate of MACCE at 5-year follow up.

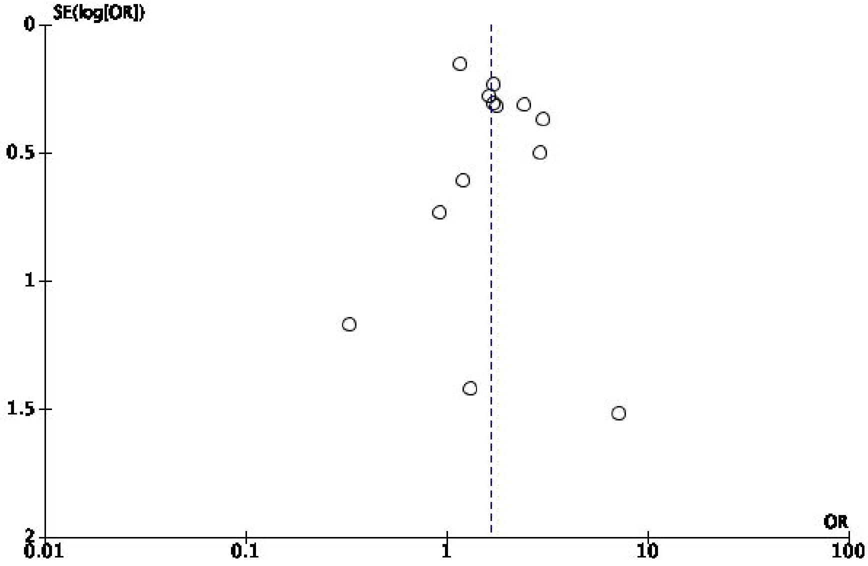

S. Figure 20: Funnel plot showing minimal publication bias across studies comparing the pooled estimate of MI at 5-year follow up.

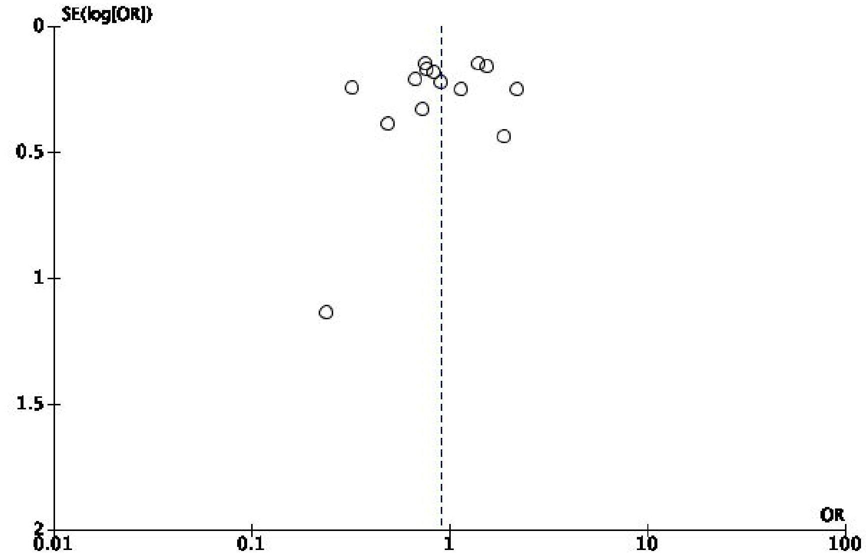

S. Figure 21: Funnel plot showing minimal publication bias across studies comparing the pooled estimate of mortality at a 5-year follow up.

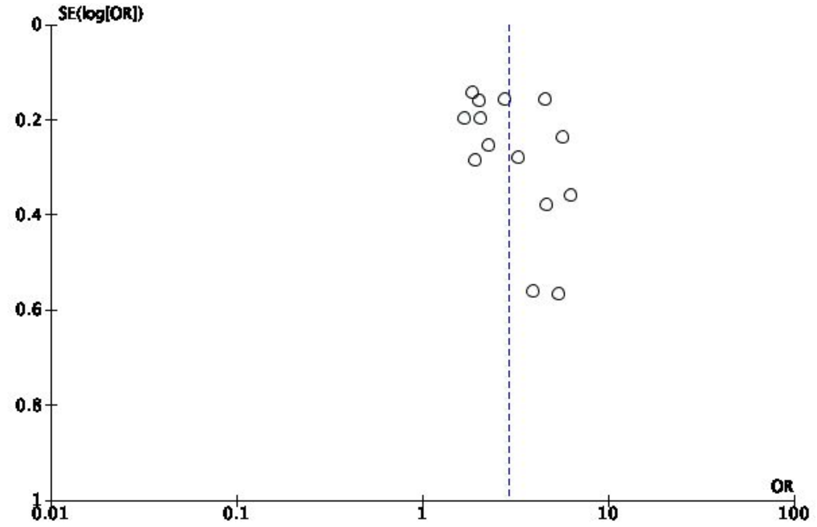

S. Figure 22: Funnel plot showing minimal publication bias across studies comparing the pooled estimate of revascularization at 5-year follow up.

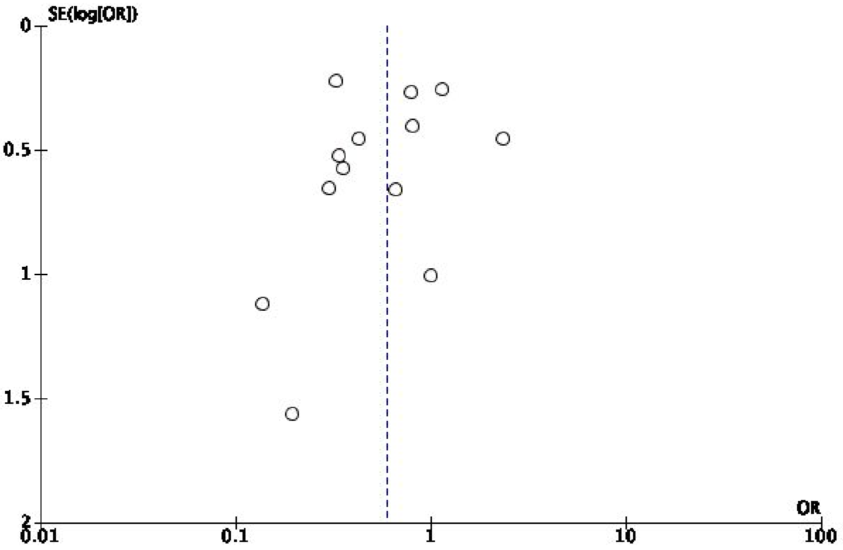

S. Figure 23: Funnel plot showing minimal publication bias across studies comparing the pooled estimate of a stroke at 5-year follow up.

E. REFERENCES:

1. Ahn JM, Roh JH, Kim YH, Park DW, Yun SC, Lee PH, Chang M, Park HW, Lee SW, Lee CW, Park SW, Choo SJ, Chung C, Lee J, Lim DS, Rha SW, Lee SG, Gwon HC, Kim HS, Chae IH, Jang Y, Jeong MH, Tahk SJ, Seung KB, Park SJ. Randomized trial of stents versus bypass surgery for left main coronary artery disease: 5-year outcomes of the PRECOMBAT study. J Am Coll Cardiol 2015;65:2198–2206.
2. Boudriot E, Thiele H, Walther T, Liebetrau C, Boeckstegers P, Pohl T, Reichart B, Mudra H, Beier F, Gansera B, Neumann FJ. Randomized comparison of percutaneous coronary intervention with sirolimus-eluting stents versus coronary artery bypass grafting in unprotected left main stem stenosis. Journal of the American College of Cardiology. 2011 Feb 1;57(5):538-45.
3. Buszman PE, Buszman PP, Banasiewicz-Szkróbka I, et al. Left main stenting in comparison with surgical revascularization: 10-year outcomes of the (Left Main Coronary Artery Stenting) LE MANS trial. JACC: Cardiovascular Interventions. 2016 Feb 22;9(4):318-27.
4. Holm NR, Mäkikallio T, Lindsay MM, Spence MS, Erglis A, Menown IB, Trovik T, Kellerth T, Kalinauskas G, Mogensen LJ, Nielsen PH. Percutaneous coronary angioplasty versus coronary artery bypass grafting in the treatment of unprotected left main stenosis: updated 5-year outcomes from the randomised, non-inferiority NOBLE trial. The Lancet. 2020 Jan 18;395(10219):191-9.
5. Makikallio T, Holm NR, Lindsay M et al. Percutaneous coronary angioplasty versus coronary artery bypass grafting in the treatment of unprotected left main stenosis (NOBLE): a prospective,

randomised, open-label, non-inferiority trial. Lancet 2016;388:2743-2752.

6. Morice MC, Serruys PW, Kappetein AP, Feldman TE, Ståhle E, Colombo A, Mack MJ, Holmes DR, Choi JW, Ruzyllo W, Religa G. Five-year outcomes in patients with left main disease treated with either percutaneous coronary intervention or coronary artery bypass grafting in the synergy between percutaneous coronary intervention with taxus and cardiac surgery trial. Circulation. 2014 Jun 10;129(23):2388-94.
7. Park SJ, Kim YH, Park DW, et al. Randomized trial of stents versus bypass surgery for left main coronary artery disease. *N Eng J Med*. 2011 May 5;364(18):1718-27.
8. Stone GW, Kappetein AP, Sabik JF, et al. Five-year outcomes after PCI or CABG for left main coronary disease. New England Journal of Medicine. 2019 Nov 7;381(19):1820-30
9. Stone GW, Sabik JF, Serruys PW, et al. Everolimus-Eluting Stents or Bypass Surgery for Left Main Coronary Artery Disease. *N Eng J Med* 2016;375:2223-35.
10. Serruys PW, Morice MC, Kappetein AP, Colombo A, Holmes DR, Mack MJ, Ståhle E, Feldman TE, Van Den Brand M, Bass EJ, Van Dyck N. Percutaneous coronary intervention versus coronary-artery bypass grafting for severe coronary artery disease. New England Journal of Medicine. 2009 Mar 5;360(10):961-72.
11. Thuijs DJ, Kappetein AP, Serruys PW, Mohr FW, Morice MC, Mack MJ, Holmes Jr DR, Curzen N, Davierwala P, Noack T, Milojevic M. Percutaneous coronary intervention versus coronary artery bypass grafting in patients with three-vessel or left main coronary artery disease: 10-year follow-up of the multicentre randomised controlled SYNTAX trial. The Lancet. 2019 Oct 12;394(10206):1325-34.
12. Brener SJ, Galla JM, Bryant III R, Sabik III JF, Ellis SG. Comparison of percutaneous versus surgical revascularization of severe unprotected left main coronary stenosis in matched patients. The American journal of cardiology. 2008 Jan 15;101(2):169-72.
13. Buchanan GL, Chieffo A, Meliga E, Mehran R, Park SJ, Onuma Y, Capranzano P, Valgimigli M, Narbute I, Makkar RR, Palacios IF, Kim YH, Buszman PP, Chakravarty T, Sheiban I, Naber C, Margey R, Agnihotri A, Marra S, Capodanno D, Allgar V, Leon MB, Moses JW, Fajadet J, Lefevre T, Morice MC, Erglis A, Tamburino C, Alfieri O, Serruys PW, Colombo A. Comparison of percutaneous coronary intervention (with drug-eluting stents) versus coronary artery bypass grafting in women with severe narrowing of the left main coronary artery (from the Women-Drug-Eluting stent for LefT main coronary Artery disease Registry). Am J Cardiol 2014;113:1348–1355
14. Caggegi A, Capodanno D, Capranzano P, Chisari A, Ministeri M, Mangiameli A, Ronsivalle G, Ricca G, Barrano G, Monaco S, Di Salvo ME. Comparison of one-year outcomes of percutaneous coronary intervention versus coronary artery bypass grafting in patients with unprotected left main coronary artery disease and acute coronary syndromes (from the CUSTOMIZE Registry). The American journal of cardiology. 2011 Aug 1;108(3):355-9.
15. Cavalcante R, Sotomi Y, Lee CW, et al. Outcomes after percutaneous coronary intervention or bypass surgery in patients with unprotected left main disease. J Am Coll Cardiol 2016;68:999–1009.
16. Chang K, Koh YS, Jeong SH, et al. Long-term outcomes of percutaneous coronary intervention versus coronary artery bypass grafting for unprotected left main coronary bifurcation disease in the drug-eluting stent era. Heart 2012;98:799–805.
17. Cheng CI, Lee FY, Chang JP, et al. Long-term outcomes of intervention for unprotected left main coronary artery stenosis: coronary stenting vs coronary artery bypass grafting. Circ J 2009;73:705–12.
18. Chieffo A, Meliga E, Latib A, et al. Drug-eluting stent for left main coronary artery disease. The DELTA registry: a multicenter registry evaluating percutaneous coronary intervention versus coronary artery bypass grafting for left main treatment. JACC Cardiovasc Interv 2012;5:718–27.
19. Ghenim R, Roncalli J, Tidjane AM, et al. One-year follow-up of nonrandomized comparison between coronary artery bypass grafting surgery and drug-eluting stent for the treatment of unprotected left main coronary artery disease in elderly patients (aged > or=75 years). J Interv Cardiol

2009;22:520–6
20. Kang SH, Park KH, Choi DJ, et al. Coronary artery bypass grafting versus drug-eluting stent implantation for left main coronary artery disease (from a two-center registry). Am J Cardiol 2010;105:343–51.
21. Kawecki D, Morawiec B, Fudal M, et al. Comparison of coronary artery bypass grafting with percutaneous coronary intervention for unprotected left main coronary artery disease. Yonsei Med J 2012;53:58–67.
22. Kim WJ, Park DW, Yun SC, Lee JY, Lee SW, Kim YH, Lee CW, Park SW, Park SJ. Impact of diabetes mellitus on the treatment effect of percutaneous or surgical revascularization for patients with unprotected left main coronary artery disease: a subgroup analysis of the MAIN-COMPARE study. JACC: Cardiovascular Interventions. 2009 Oct 1;2(10):956-63.
23. Lee MS, Jamal F, Kedia G et al (2007) Comparison of bypass surgery with drug-eluting stents for diabetic patients with multivessel disease. Int J Cardiol 123:34–42
24. Lu TM, Lee WL, Hsu PF, et al. Long-term results of stenting versus coronary artery bypass surgery for left main coronary artery disease—a single-center experience. J Chin Med Assoc 2016;79:356–62.
25. Luo Y, Yu X, Chen F, Du X, He J, Gao Y, Zhang X, Zhang Y, Ren X, Lv S, Ma C. Impact of diabetes mellitus on patients with unprotected left main coronary artery lesion disease treated with either percutaneous coronary intervention or coronary-artery bypass grafting. Coronary artery disease. 2012 Aug 1;23(5):322-9.
26. Mäkikallio TH, Niemelä M, Kervinen K, et al. Coronary angioplasty in drug eluting stent era for the treatment of unprotected left main stenosis compared to coronary artery bypass grafting. Ann Med 2008;40: 437–43.
27. Montalescot G, Brieger D, Eagle KA, Anderson Jr FA, FitzGerald G, Lee MS, Steg PG, Avezum A, Goodman SG, Gore JM, GRACE Investigators. Unprotected left main revascularization in patients with acute coronary syndromes. European heart journal. 2009 Aug 30;30(19):2308-17.
28. Naganuma T, Chieffo A, Meliga E, et al. Long-term clinical outcomes after percutaneous coronary intervention versus coronary artery bypass grafting for ostial/midshaft lesions in unprotected left main coronary artery from the DELTA registry: a multicenter registry evaluating percutaneous coronary intervention versus coronary artery bypass grafting for left main treatment. JACC Cardiovasc Interv 2014;7: 354–61.
29. Palmerini T, Marzocchi A, Marrozzini C. Comparison between coronary angioplasty and coronary artery bypass surgery for the treatment of unprotected left main coronary artery stenosis (the Bologna Registry). Am J Cardiol 2006;98:54–9.
30. Park DW, Kim YH, Yun SC, et al. Long-term outcomes after stenting versus coronary artery bypass grafting for unprotected left main coronary artery disease: 10-year results of bare-metal stents and 5-year results of drug-eluting stents from the ASAN-MAIN (ASAN Medical Center-Left MAIN Revascularization) Registry. J Am Coll Cardiol 2010;56:1366–75
31. Park DW, Seung KB, Kim YH, Lee JY, Kim WJ, Kang SJ, Lee SW, Lee CW, Park SW, Yun SC, Gwon HC. Long-term safety and efficacy of stenting versus coronary artery bypass grafting for unprotected left main coronary artery disease: 5-year results from the MAIN-COMPARE (Revascularization for Unprotected Left Main Coronary Artery Stenosis: Comparison of Percutaneous Coronary Angioplasty Versus Surgical Revascularization) registry. Journal of the American College of Cardiology. 2010 Jul 6;56(2):117-24.
32. Park SJ, Kim YH, Park DW, Yun SC, Ahn JM, Song HG, Lee JY, Kim WJ, Kang SJ, Lee SW, Lee CW. Randomized trial of stents versus bypass surgery for left main coronary artery disease. New England Journal of Medicine. 2011 May 5;364(18):1718-27.
33. Qin Q, Qian J, Wu X, et al. A comparison between coronary artery bypass grafting surgery and percutaneous coronary intervention for the treatment of unprotected left main coronary artery

disease. Clin Cardiol 2013;36:54–60.
34. Rittger H, Rieber J, Kögler K, Sinha A, Schmidt M, Breithardt OA, et al. Clinical outcome and quality of life after interventional treatment of left main disease with drug-eluting-stents in comparison to CABG in elderly and younger patients. Clin Res Cardiol 2011; 100: 439–446.
35. Rodés-Cabau J, Deblois J, Bertrand OF, Mohammadi S, Courtis J, Larose E, Dagenais F, Déry JP, Mathieu P, Rousseau M, Barbeau G, Baillot R, Gleeton O, Perron J, Nguyen CM, Roy L, Doyle D, De Larochellière R, Bogaty P, Voisine P. Nonrandomized comparison of coronary artery bypass surgery and percutaneous coronary intervention for the treatment of unprotected left main coronary artery disease in octogenarians. Circulation 2008;118:2374e2381.
36. Sanmartin M, Baz JA, Claro R, et al. Comparison of drug-eluting stents versus surgery for unprotected left main coronary artery disease. Am J Cardiol 2007;100:970–3.
37. Shimizu T, Ohno T, Ando J, et al. Mid-term results and costs of coronary artery bypass vs drug-eluting stents for unprotected left main coronary artery disease. Circ J 2010;74:449–55.
38. Shiomi H, Morimoto T, Furukawa Y, et al. Comparison of percutaneous coronar intervention with coronary artery bypass grafting in unprotected left main coronary artery disease—5-year outcome from CREDO-Kyoto PCI/CABG Registry Cohort-2. Circ J 2015;79:1282–9.
39. Te Hsu J, Chu CM, Chang ST, Kao CL, Chung CM. Percutaneous coronary intervention versus coronary artery bypass graft surgery for the treatment of unprotected left main coronary artery stenosis. International heart journal. 2008;49(3):355-70.
40. Wei Z, Xie J, Wang K, et al. Comparison of percutaneous coronary intervention versus coronary artery bypass graft in aged patients with unprotected left main artery lesions. Int Heart J 2016;57:682–8.
41. White AJ, Kedia G, Mirocha JM, Lee MS, Forrester JS, Morales WC, Dohad S, Kar S, Czer LS, Fontana GP, Trento A. Comparison of coronary artery bypass surgery and percutaneous drug-eluting stent implantation for treatment of left main coronary artery stenosis. JACC: Cardiovascular Interventions. 2008 Jun 1;1(3):236-45.
42. Wu C, Hannan EL, Walford G, et al. Utilization and outcomes of unprotected left main coronary artery stenting and coronary artery bypass graft surgery. Ann Thorac Surg 2008;86:1153–9.
43. Wu X, Chen Y, Kubo T, et al. Long-term (4-year) outcomes and predictors of adverse cardiac events after sirolimus-eluting stent implantation in unprotected left main coronary artery. Int Heart J 2010;51:377–82.
44. Yi G, Youn YN, Hong S, et al. Midterm outcome of off-pump bypass procedures versus drug-eluting stent for unprotected left main coronary artery disease. Ann Thorac Surg 2012;94:15–22.
45. Yin Y, Xin X, Geng T, et al. Clinical comparison of percutaneous coronary intervention with domestic drug-eluting stents versus off pump coronary artery bypass grafting in unprotected left main coronary artery disease. Int J Clin Exp Med 2015;8:14376–82.
46. Yu XP, Wu CY, Ren XJ, et al. Very long-term outcomes and predictors of percutaneous coronary intervention with drug-eluting stents versus coronary artery bypass grafting for patients with unprotected left main coronary artery disease. Chin Med J (Engl) 2016;129:763–70.
47. Zhao X, Zhou Y, Song H, Guan L, Zheng G, Jin Z, Shi D, Li Y, Guo Y, Shi GP, Cheng XW. Comparison of bypass surgery with drug-eluting stents in diabetic patients with left main coronary stenosis. Yonsei medical journal. 2011 Nov 1;52(6):923-32.
48. Zheng Z, Xu B, Zhang H, et al. Coronary artery bypass graft surgery and percutaneous coronary interventions in patients with unprotected left main coronary artery disease. JACC Cardiovasc Interv 2016;9:1102–11.
49. De Rosa S, Polimeni A, Sabatino J, Indolfi C. Long-term outcomes of coronary artery bypass grafting versus stent-PCI for unprotected left main disease: a meta-analysis. BMC cardiovascular disorders. 2017 Dec;17(1):240.

50. Kodumuri V, Balasubramanian S, Vij A, Siddamsetti S, Sethi A, Khalafallah R, Khosla S. A meta-analysis comparing percutaneous coronary intervention with drug-eluting stents versus coronary artery bypass grafting in unprotected left main disease. The American journal of cardiology. 2018 Apr 15;121(8):924-33.
51. Bittl JA, He Y, Jacobs AK, Yancy CW, Normand SL. Bayesian methods affirm the use of percutaneous coronary intervention to improve survival in patients with unprotected left main coronary artery disease. Circulation. 2013 Jun 4;127(22):2177-85.
52. Bajaj NS, Patel N, Kalra R, Marogil P, Bhardwaj A, Arora G, Arora P. Percutaneous coronary intervention vs. coronary artery bypass grafting for left main revascularization: an updated meta-analysis. European Heart Journal-Quality of Care and Clinical Outcomes. 2017 Jul 1;3(3):173-82.
53. Upadhaya S, Baniya R, Madala S, Subedi SK, Khan J, Velagapudi RK, Bachuwa G. Drug-eluting stent placement versus coronary artery bypass surgery for unprotected left main coronary artery disease: A meta-analysis of randomized controlled trials. Journal of cardiac surgery. 2017 Feb;32(2):70-9
54. Khan SU, Rahman H, Arshad A, Khan MU, Lekkala M, Yang T, Mishra A, Kaluski E. Percutaneous Coronary Intervention Versus Surgery in Left Main Stenosis–A Meta-Analysis and Systematic Review of Randomised Controlled Trials. Heart, Lung and Circulation. 2018 Feb 1;27(2):138-46.
55. Zhang XL, Zhu QQ, Yang JJ, Chen YH, Li Y, Zhu SH, Xie J, Wang L, Kang LN, Xu B. Percutaneous intervention versus coronary artery bypass graft surgery in left main coronary artery stenosis: a systematic review and meta-analysis. BMC medicine. 2017 Dec;15(1):84.
56. Kajimoto K, Miyauchi K, Yamamoto T, Daida H, Amano A. Meta-analysis of Randomized Controlled Trials on the Treatment of Unprotected Left Main Coronary Artery Disease: One-Year Outcomes with Coronary Artery Bypass Grafting Versus Percutaneous Coronary Artery Intervention with Drug-Eluting Stent. Journal of cardiac surgery. 2012 Mar;27(2):152-7.
57. Jang JS, Choi KN, Jin HY, Seo JS, Yang TH, Kim DK, Kim DS, Urm SH, Chun JH, Kang SJ, Park DW. Meta-analysis of three randomized trials and nine observational studies comparing drug-eluting stents versus coronary artery bypass grafting for unprotected left main coronary artery disease. The American journal of cardiology. 2012 Nov 15;110(10):1411-8.
58. Tamburino C, Capodanno D, Di Salvo ME, Caggegi A, Tomasello D, Cincotta G, Miano M, Petralia A, Varone E, Patanè M, Tamburino C. Routine versus selective coronary artery bypass for left main coronary artery revascularization: The appraise a customized strategy for left main revascularization (CUSTOMIZE) study. International journal of cardiology. 2011 Aug 4;150(3):307-14.
59. Alam M, Huang HD, Shahzad SA, Kar B, Virani SS, Rogers PA, Paniagua D, Bozkurt B, Palacios I, Kleiman NS, Jneid H. Percutaneous coronary intervention vs. coronary artery bypass graft surgery for unprotected left main coronary artery disease in the drug-eluting stents era. Circulation journal. 2012:CJ-12.
60. Capodanno D, Stone GW, Morice MC, Bass TA, Tamburino C. Percutaneous coronary intervention versus coronary artery bypass graft surgery in left main coronary artery disease: a meta-analysis of randomized clinical data. Journal of the American College of Cardiology. 2011 Sep 27;58(14):1426-32.
61. Biondi-Zoccai GG, Lotrionte M, Moretti C, Meliga E, Agostoni P, Valgimigli M, Migliorini A, Antoniucci D, Carrié D, Sangiorgi G, Chieffo A. A collaborative systematic review and meta-analysis on 1278 patients undergoing percutaneous drug-eluting stenting for unprotected left main coronary artery disease. American heart journal. 2008 Feb 1;155(2):274-83.
62. Palmerini T, Serruys P, Kappetein AP, Genereux P, Della Riva D, Reggiani LB, Christiansen EH, Holm NR, Thuesen L, Makikallio T, Morice MC. Clinical outcomes with percutaneous coronary revascularization vs coronary artery bypass grafting surgery in patients with unprotected left main

coronary artery disease: a meta-analysis of 6 randomized trials and 4,686 patients. American heart journal. 2017 Aug 1;190:54-63.

63. Ye Y, Yang M, Zhang fS, Zeng Y. Percutaneous coronary intervention versus cardiac bypass surgery for left main coronary artery disease: A trial sequential analysis. Medicine. 2017 Oct;96(41).
64. Ali WE, Vaidya SR, Ejeh SU, Okoroafor KU. Meta-analysis study comparing percutaneous coronary intervention/drug eluting stent versus coronary artery bypass surgery of unprotected left main coronary artery disease: clinical outcomes during short-term versus long-term (> 1 year) follow-up. Medicine. 2018 Feb;97(7).
65. Naik H, White AJ, Chakravarty T, Forrester J, Fontana G, Kar S, Shah PK, Weiss RE, Makkar R. A meta-analysis of 3,773 patients treated with percutaneous coronary intervention or surgery for unprotected left main coronary artery stenosis. JACC: Cardiovascular Interventions. 2009 Aug 1;2(8):739-47.
66. Lee MS, Yang T, Dhoot J, Liao H. Meta-analysis of clinical studies comparing coronary artery bypass grafting with percutaneous coronary intervention and drug-eluting stents in patients with unprotected left main coronary artery narrowings. The American journal of cardiology. 2010 Apr 15;105(8):1070-5.
67. Alam M, Virani SS, Shahzad SA, Siddiqui S, Siddiqui KH, Mumtaz SA, Kleiman NS, Coselli JS, Lakkis NM, Jneid H. Comparison by meta-analysis of percutaneous coronary intervention versus coronary artery bypass grafting in patients with a mean age of≥ 70 years. The American journal of cardiology. 2013 Sep 1;112(5):615-22.
68. Athappan G, Patvardhan E, Tuzcu ME, Ellis S, Whitlow P, Kapadia SR. Left main coronary artery stenosis: a meta-analysis of drug-eluting stents versus coronary artery bypass grafting. JACC: Cardiovascular Interventions. 2013 Dec 1;6(12):1219-30.
69. Sá MP, Soares AM, Lustosa PC, Martins WN, Browne F, Ferraz PE, Vasconcelos FP, Lima RC. Meta-analysis of 5674 patients treated with percutaneous coronary intervention and drug-eluting stents or coronary artery bypass graft surgery for unprotected left main coronary artery stenosis. European Journal of Cardio-Thoracic Surgery. 2012 Apr 19;43(1):73-80.
70. Al Ali J, Franck C, Filion KB, Eisenberg MJ. Coronary artery bypass graft surgery versus percutaneous coronary intervention with first-generation drug-eluting stents: a meta-analysis of randomized controlled trials. JACC: Cardiovascular Interventions. 2014 May 1;7(5):497-506.
71. Lee CW, Ahn JM, Cavalcante R, Sotomi Y, Onuma Y, Suwannasom P, Tenekecioglu E, Yun SC, Park DW, Kang SJ, Lee SW. Coronary artery bypass surgery versus drug-eluting stent implantation for left main or multivessel coronary artery disease: a meta-analysis of individual patient data. JACC: Cardiovascular Interventions. 2016 Dec 19;9(24):2481-9.
72. Khan MR, Kayani WT, Ahmad W, Hira RS, Virani SS, Hamzeh I, Jneid H, Lakkis N, Alam M. Meta-analysis of comparison of 5-year outcomes of percutaneous coronary intervention versus coronary artery bypass grafting in patients with unprotected left main coronary artery in the era of drug-eluting stents. The American journal of cardiology. 2017 Nov 1;120(9):1514-20.
73. Testa L, Latib A, Bollati M, Montone RA, Colombo A, Crea F, Bedogni F. Unprotected left main revascularization: Percutaneous coronary intervention versus coronary artery bypass. An updated systematic review and meta-analysis of randomised controlled trials. PloS one. 2017 Jun 28;12(6):e0179060.
74. Putzu A, Gallo M, Martino EA, Ferrari E, Pedrazzini G, Moccetti T, Cassina T. Coronary artery bypass graft surgery versus percutaneous coronary intervention with drug-eluting stents for left main coronary artery disease: a meta-analysis of randomized trials. International journal of cardiology. 2017 Aug 15;241:142-8.

75. Sharma SP, Dahal K, Khatra J, Rosenfeld A, Lee J. Percutaneous coronary intervention vs coronary artery bypass grafting for left main coronary artery disease? A systematic review and meta-analysis of randomized controlled trials. Cardiovascular therapeutics. 2017 Jun;35(3):e12260.
76. Spinthakis N, Farag M, Gorog DA, Prasad A, Mahmood H, Gue Y, Wellsted D, Nabhan A, Srinivasan M. Percutaneous coronary intervention with drug-eluting stent versus coronary artery bypass grafting: A meta-analysis of patients with left main coronary artery disease. International journal of cardiology. 2017 Dec 15;249:101-6.
77. Moore P, Burrage M, Garrahy P, Lim R, McCann A, Camuglia A. Drug-Eluting Stents Versus Coronary Artery Bypass Grafts for Left Main Coronary Disease: A Meta-Analysis and Review of Randomised Controlled Trials. Heart, Lung and Circulation. 2018 Dec 1;27(12):1437-45.
78. Garg A, Rao SV, Agrawal S, Theodoropoulos K, Mennuni M, Sharma A, Garg L, Ferrante G, Meelu OA, Sargsyan D, Reimers B. Meta-analysis of randomized controlled trials of percutaneous coronary intervention with drug-eluting stents versus coronary artery bypass grafting in left main coronary artery disease. The American journal of cardiology. 2017 Jun 15;119(12):1942-8.
79. Shah R, Morsy MS, Weiman DS, Vetrovec GW. Meta-analysis comparing coronary artery bypass grafting to drug-eluting stents and to medical therapy alone for left main coronary artery disease. The American journal of cardiology. 2017 Jul 1;120(1):63-8.
80. Mahmoud AN, Elgendy IY, Mentias A, Saad M, Ibrahim W, Mojadidi MK, Nairooz R, Eshtehardi P, David Anderson R, Samady H. Percutaneous coronary intervention or coronary artery bypass grafting for unprotected left main coronary artery disease. Catheterization and Cardiovascular Interventions. 2017 Oct 1;90(4):541-52.
81. Khan A. R., Golwala H., Tripathi A., et al. Meta-analysis of percutaneous coronary intervention versus coronary artery bypass grafting in left main coronary artery disease. *American Journal of Cardiology*. 2017;119(12):1949–1956. doi: 10.1016/j.amjcard.2017.03.022
82. Nerlekar N, Ha FJ, Verma KP, Bennett MR, Cameron JD, Meredith IT, Brown AJ. Percutaneous coronary intervention using drug-eluting stents versus coronary artery bypass grafting for unprotected left main coronary artery stenosis: a meta-analysis of randomized trials. Circulation: Cardiovascular Interventions. 2016 Dec;9(12):e004729.
83. Giacoppo D, Colleran R, Cassese S, Frangieh AH, Wiebe J, Joner M, Schunkert H, Kastrati A, Byrne RA. Percutaneous coronary intervention vs coronary artery bypass grafting in patients with left main coronary artery stenosis: a systematic review and meta-analysis. JAMA cardiology. 2017 Oct 1;2(10):1079-88.
84. Palmerini T, Biondi-Zoccai G, Reggiani LB, Sangiorgi D, Alessi L, De Servi S, Branzi A, Stone GW. Risk of stroke with coronary artery bypass graft surgery compared with percutaneous coronary intervention. Journal of the American College of Cardiology. 2012 Aug 28;60(9):798-805.
85. Wang Z, Zhan B, Bao H, Huang X, Wu Y, Liang Q, Zhang W, Jiang L, Cheng X. Percutaneous Coronary Intervention Versus Coronary Artery Bypass Grafting in Unprotected Left Main Coronary Artery Stenosis. The American journal of the medical sciences. 2019 Mar 1;357(3):230-41.
86. Ahmad Y, Howard JP, Arnold AD, et al. Mortality after drug-eluting stents vs. coronary artery bypass grafting for left main coronary artery disease: a meta-analysis of randomized controlled trials Eur Heart J. 2020, doi: 10.1093/eurheartj/ehaa135
